# Design, Synthesis, and Evaluation of Dihydropyranopyrazole Derivatives as Novel PDE2 Inhibitors for the Treatment of Alzheimer’s Disease

**DOI:** 10.3390/molecules26103034

**Published:** 2021-05-19

**Authors:** Yan Zhou, Jinjian Li, Han Yuan, Rui Su, Yue Huang, Yiyou Huang, Zhe Li, Yinuo Wu, Haibin Luo, Chen Zhang, Ling Huang

**Affiliations:** 1 School of Pharmaceutical Sciences, Sun Yat-Sen University, Guangzhou 510006, China; zhouy598@163.com (Y.Z.); lijinj15@163.com (J.L.); yuanhan0806@163.com (H.Y.); surui20@163.com (R.S.); huangy266@mail2.sysu.edu.cn (Y.H.); huangyy287@mail.sysu.edu.cn (Y.H.); lizhe5@mail.sysu.edu.cn (Z.L.); wyinuo3@mail.sysu.edu.cn (Y.W.); luohb77@mail.sysu.edu.cn (H.L.); 2 School of Pharmaceutical Sciences, Hainan University, Haikou 510006, China

**Keywords:** dihydropyranopyrazole derivatives, PDE2 inhibitors, Alzheimer’s disease

## Abstract

Phosphodiesterase 2 (PDE2) has been regarded as a novel target for the treatment of Alzheimer’s disease (AD). In this study, we obtained (***R***)-**LZ77** as a hit compound with moderate PDE2 inhibitory activity (IC_50_ = 261.3 nM) using a high-throughput virtual screening method based on molecular dynamics. Then, we designed and synthesized 28 dihydropyranopyrazole derivatives as PDE2 inhibitors. Among them, compound (***+***)-**11h** was the most potent PDE2 inhibitor, with an IC_50_ value of 41.5 nM. The molecular docking of PDE2-(***+***)-**11h** reveals that the 4-(trifluoromethyl)benzyl)oxyl side chain of the compound enters the H-pocket and forms strong hydrophobic interactions with L770/L809/F862, which improves inhibitory activity. The above results may provide insight for further structural optimization of highly potent PDE2 inhibitors and may lay the foundation for their use in the treatment of AD.

## 1. Introduction

Alzheimer’s disease (AD) is a progressive neurodegenerative disease with clinical manifestations that include memory loss; cognitive dysfunction; disorientation; and loss of language, writing, and logical reasoning skills [1,2]. The pathogenesis of Alzheimer’s disease is complex and involves environmental and genetic factors, which, to date, have not been fully clarified. In the past few decades, researchers have proposed several hypotheses, including the cholinergic hypothesis [3], amyloid cascade hypothesis [4], and inflammation hypothesis [5]. Currently, two types of drugs are approved by the Food and Drug Administration (FDA) for the treatment of AD: cholinesterase inhibitors (ChEI) and *N*-methyl-d-aspartate (NMDA) receptor antagonists, such as donepezil, galantamine, rivastigmine, and memantine, which can improve memory and cognitive function to a certain extent but cannot definitively cure this disease [6]. Therefore, developing safe and effective drugs for the treatment of AD is of great significance.

Phosphodiesterases (PDEs) are a group of enzymes that regulate cell signaling and synaptic transmission by hydrolyzing the phosphodiester bonds of cGMP and cAMP, which act as second messengers [7]. A large number of studies have indicated that PDE inhibitors play an important role in neuroprotection and the enhancement of neuroplasticity [8,9,10]. PDE2, which is a dual-substrate enzyme that hydrolyzes both cAMP and cGMP, has received extensive attention due to its high levels of expression in the forebrain, especially in the hippocampus and cortex, and in other parts of the brain [11]. Recent studies clearly indicate that PDE2 inhibitors can regulate neuronal plasticity, reduce tau phosphorylation and beta amyloid (Aβ) aggregation, promote inflammatory cytokines and apoptosis, increase the levels of Brain-Derived Neurotrophic Factor (BDNF) and cAMP/cGMP, and improve cognitive impairment [11,12,13,14]. Therefore, PDE2 has been regarded as a novel target for the treatment of AD.

**Figure 1:**
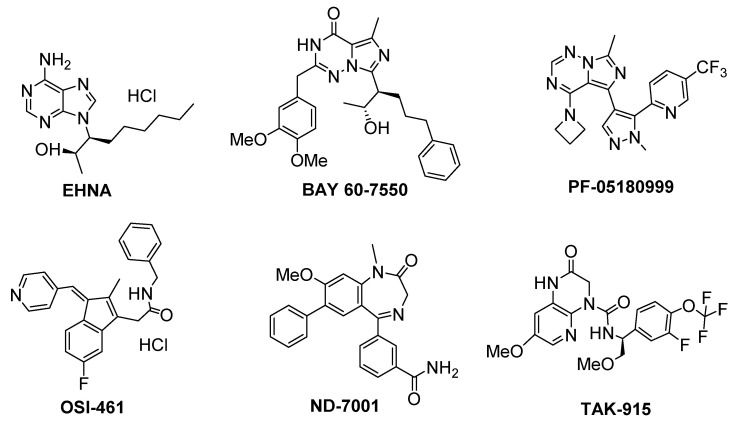
Chemical structures of several reported PDE2 inhibitors.

Although PDE2 was discovered some time ago, research on PDE2 inhibitors only started in the 2000s. The determination of the crystal structure of PDE2 facilitated the development of PDE2 inhibitors (Figure 1) [15,16]. **EHNA** was first discovered as an inhibitor of PDE2 [17]. Its application was restricted to its adenosine deaminase inhibitory activity [11]. Then, studies clearly indicated that **BAY 60-7550**, the first selective PDE2 inhibitor reported by Bayer in 2002, could reverse functional impairments by decreasing hippocampal neurodegeneration and by enhancing hippocampal neuronal plasticity. However, the development of **BAY 60-7550** was terminated due to its shortcomings in solubility and pharmacokinetics [18,19]. **PF-05180999**, another PDE2 inhibitor, was developed by Pfizer and entered the first phase of clinical trials as a treatment for schizophrenia and migraine, but there have been no further reports [7]. Thus, the development of selective PDE2 inhibitors is of great significance.

In previous research, we developed an efficient virtual screening method based on molecular dynamics and successfully applied it to discover highly potent PDE10 inhibitors [20,21]. In this study, we used this method to obtain (***R***)-**LZ77** as a hit compound with moderate PDE2 inhibitory activity (IC_50_ = 261.3 nM) through high-throughput screening. In order to improve its activity and selectivity, we designed and synthesized a series of dihydropyranopyrazole derivatives based on the structure of (***R***)-**LZ77**, aiming to obtain compounds with better inhibitory activity against PDE2. Then, we evaluated the inhibitory activity of target compounds against PDE2 and their selectivity relative to other PDEs. Furthermore, molecular docking studies and decomposition of binding free energies were also performed to explore the binding modes of PDE2 and inhibitors. 

**Figure 2:**
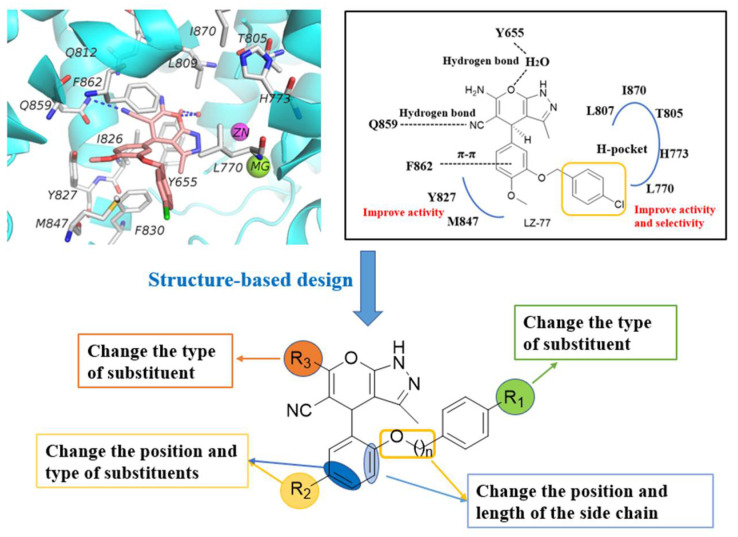
Structure and putative binding mode of (***R***)-**LZ77** with PDE2 according to molecular docking studies and the design strategy for PDE2 inhibitors.

According to the molecular docking of (***R***)-**LZ77** and PDE2 (Figure 2), we found that (1) the hit compound (***R***)-**LZ77** forms π–π stacking interactions with F862 through the benzene ring in the middle and forms a hydrogen bond with Q859 through the cyano group on the pyranopyrazole; (2) the oxygen atom on the (***R***)-**LZ77** pyranopyrazole skeleton forms a hydrogen bond with Y655 through crystallization water; and (3) the benzyl side chain extends outside of the pocket without entering the hydrophobic pocket (H pocket) formed by L770/H773/T805/L809/F862/I870. Therefore, if a side chain can be introduced to (***R***)-**LZ77** and simultaneously occupy the H pocket, it can significantly improve the inhibitory activity against PDE2 and the selectivity relative to other PDEs. 

In this research, in order to improve activity and selectivity, first, the position and type of substituents on the benzene ring were changed. Second, the position and length of the side chains on the benzene ring connected to the pyran ring were changed to bind to the H pocket in the PDE2 catalytic pocket, thereby increasing the activity. A total of 28 compounds were designed and synthesized to evaluate their effects on inhibitory activity against PDE2, aiming to provide insights for the subsequent design of highly potent and highly selective PDE2 inhibitors.

## 2. Results

### 2.1. Chemistry

**Scheme 1:**
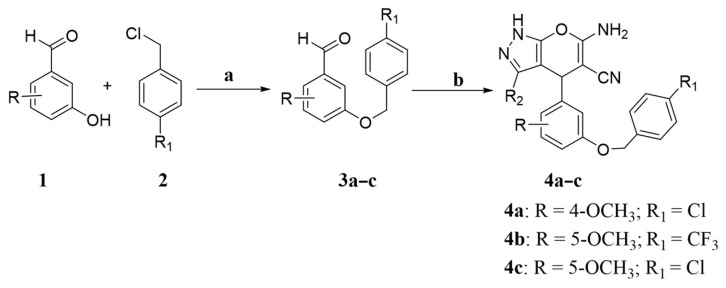
Reagents and conditions: (**a**) K_2_CO_3_, acetonitrile, 80 °C, 5 h; (**b**) malononitrile triethylamine, 3-methyl-5-pyrazolone, ethanol, 80 °C, 15 min.

The synthesis of target compounds **4a**–**c** is outlined in Scheme 1. In the presence of potassium carbonate, 2-hydroxybenzaldehyde with different substituents reacts with benzyl chloride for *O*-alkylation reaction to obtain compounds **3a**–**c**. Then, compounds **4a**–**c** are obtained through the ring-closure reaction of aromatic aldehyde, malononitrile, and pyrazolinone catalyzed by a three-component base, which involves a tandem Michael addition/Thorpe–Ziegler-type reaction to generate the dihydropyrano[2,3-*c*] pyrazole core [22].

**Scheme 2:**
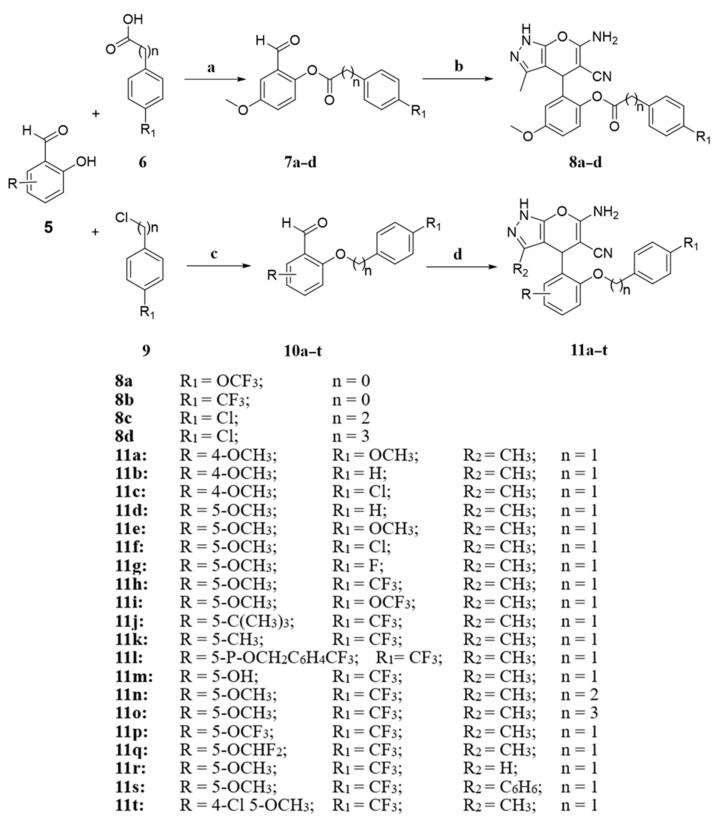
Reagents and conditions: (**a**) HATU, DIPEA, anhydrous CH_2_Cl_2_, room temperature, overnight; (**b**) malononitrile, 3-methyl-5-pyrazolone, *N*-methylmorpholine, ethanol, room temperature, overnight; (**c**) K_2_CO_3,_ acetonitrile, 80 °C, 5 h; (**d**) malononitrile, triethylamine, pyrazolinone, ethanol, 80 °C, 15 min.

The synthesis of target compounds **8a**–**d** and **11a**–**t** is illustrated in Scheme 2. In the presence of HATU and DIPEA, 2-hydroxy-5-methoxybenzaldehyde is condensed with acids that substitute different chain lengths and different substituents at room temperature to obtain a series of intermediates (**7**). Then, the series of compounds **7a**–**d** and malononitrile, *N*-methylmorpholine and 3-methyl-5-pyrazolone are ring-closed in ethanol at room temperature to obtain the target compounds **8a**–**d**. The synthesis of target compounds **11a**–**t** is similar to the synthetic method of Scheme 1, except that the substitution position of the hydroxyl group on the aromatic aldehyde is changed from 3 to 2. 

Considering the effect of chiral structure on the biological activity of compounds, the chiral isomers (**−**)-**11h** (${\left[\alpha \right]}_{D}^{20}$ = −46.7 (c 1.0, THF), t_R_ = 24.847 min, ee value, >99%) and (**+**)-**11h** (${\left[\alpha \right]}_{D}^{20}$ = +46.5 (c 1.0, THF), t_R_ = 33.976 min, ee value, >99%) were prepared by the separation of (**±**)**-11h** with chiral HPLC (HPLC chromatogram of (**±**)-**11h,** (**−**)-**11h** and (**+**)-**11h** are included in the Supplementary Materials).

**Scheme 3:**
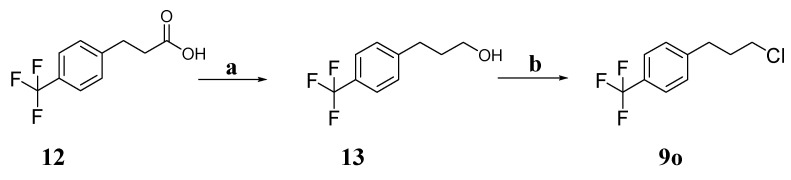
Reagents and conditions: (**a**) LiAlH_4_, THF, 85 °C, 6 h; (**b**) triphosgene, triethylamine, CH_2_Cl_2_, room temperature, 6 h.

The synthesis of intermediate **9o** is shown in Scheme 3. In the presence of tetrahydroaluminum lithium, the carboxyl group in **12** is reduced to a hydroxyl group to obtain **13**, and then, triphosgene is used for chlorination to obtain intermediate **9o**.

**Scheme 4:**
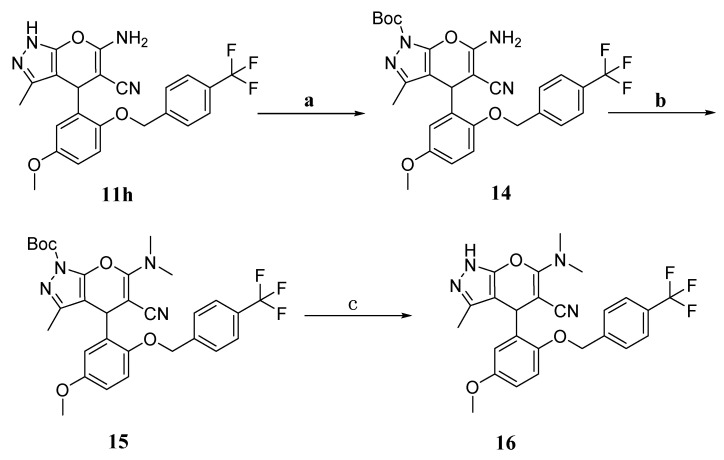
Reagents and conditions: (**a**) DMAP, (Boc)_2_O, THF, room temperature, 8 h; (**b**) CH_3_I, K_2_CO_3_, CH_3_CN, 60 °C, 5 h; (**c**) trifluoroacetic acid, DCM, room temperature, 1 h.

The synthesis of target compound **16** is summarized in Scheme 4. In the presence of DMAP, **11h** reacts with di-tert-butyl methyl dicarbonate to produce **14**. Then, **14** reacts with methyl iodide in the presence of potassium carbonate to obtain **15**, which is converted to **16** in the presence of trifluoroacetic acid.

### 2.2. Inhibitory Activity of PDE2 Inhibitors

**Figure 3:**
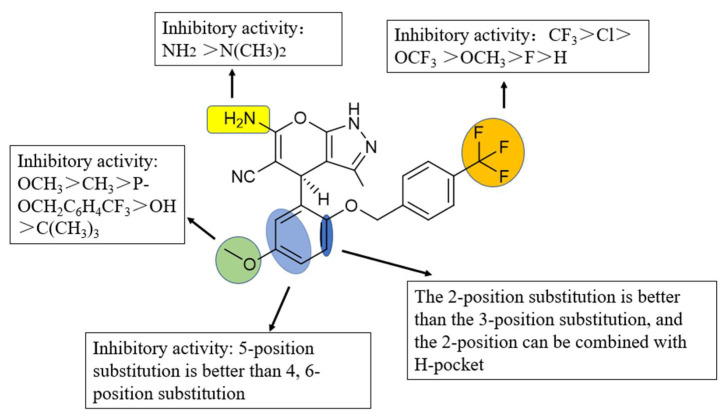
Summarized SARs of synthesized compounds.

In vitro inhibitory potencies against PDE2 of the hit compound and its synthesized derivatives were determined using **EHNA** (IC_50_ = 2460 nM), which was purchased from SIGMA and used as the reference compound. The inhibitory activities of target compounds against PDE2 are summarized in Table 1. Most of the target compounds demonstrated moderate to high inhibitory activity against PDE2. Among them, compound (***+***)**-11h** was the most potent PDE2 inhibitor, with an IC_50_ value of 41.5 nM. We summarize the structure–activity relationship as follows: (1) the activity of the compound with a substituent at the 5-position of the benzene ring connected to the pyran ring (**11f,** 79.2% inhibition at 0.5 μM) is higher than that at the 4-position (**11c,** 29.0% inhibition at 0.5 μM). (2) Among the 5-position substituents, the methoxyl group (**11h**, IC_50_ = 75.5 nM) is more active than methyl (**11k**, IC_50_ = 153.5 nM), trifluoromethoxyl (**11p,** 17.4% inhibition at 0.5 μM), difluoromethoxyl (**11q**, 6.6% inhibition at 0.5 μM), hydroxyl (**11m**, no inhibition at 0.5 μM), and tert-butyl (**11j**, no inhibition at 0.5 μM). (3) The side-chain substitution at position 2 has a better impact on inhibitory activity (**11f**, 79.2% inhibition at 0.5 μM) than that at position 3 (**4c**, 43.4% inhibition at 0.5 μM). (4) The trifluoromethyl substituent (**11h**, IC_50_ = 75.5 nM) on the benzene ring on the side chain is more active than chlorine (**11g**, IC_50_ = 807.5 nM), trifluoromethoxyl (**11i**, 48.8% inhibition at 0.5 μM), fluorine (**11j**, IC_50_ = 807.5 nM), and methoxyl (**11e**, IC_50_ = 458.4 nM). (5) When the amino group on the pyran ring (**11h**, IC_50_ = 75.5 nM) is replaced by a dimethylamino group (**16**, 9.6% inhibition at 0.5 μM), the activity decreases. (6) When the side chain is connected by an ester bond and the length of the ester bond is extended, the activity of these compounds is not improved (Figure 3). 

After these analyses, the best compound **11h** was resolved: (***+***)-**11h** had better activity, while (***−***)-**11h** was almost inactive. Notably, the most potent compound (***+***)-**11h** (IC_50_ = 41.5 nM) was more potent than the hit compound (***R***)-**LZ77** (IC_50_ = 261.3 nM). Thus, (***+***)-**11h** was identified as a potent PDE2 inhibitor for subsequent study. 

### 2.3. Remarkable Selectivity of Compound (**+**)-**11h** against PDE2 Compared with Other PDEs

The selectivity profile of compound (***+***)-**11h** against PDE2 compared with other PDE families was also evaluated, and the results are listed in Table 2. Its inhibition of PDE4D2 and PDE8A1 was very weak (IC_50_ > 10,000 nM). Its IC_50_ value against PDE1C was 289-fold higher than that against PDE2A. In short, (***+***)-**11h** has high selectivity against PDE2 compared with several other PDEs.

### 2.4. Molecular Docking

**Figure 4:**
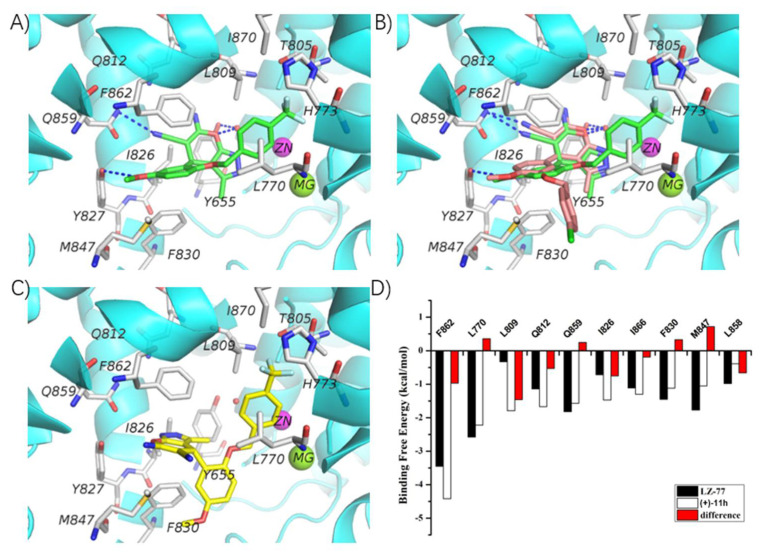
(**A**) Putative binding mode of compound (***+***)-**11h** with PDE2 in molecular docking studies. (**B**) Putative binding mode of compound (***+***)-**11h** and (***R***)-**LZ77** with PDE2 in molecular docking studies. (**C**) Putative binding mode of compound (***−***)-**11h** with PDE2 in molecular docking studies. (**D**) Decomposition of important residue contributions to the total binding free energies for (***R***)-**LZ77** and (***+***)-**11h** with PDE2.

Similar to the results of the above activity analyses, the docking results of (***+***)-**11h** with PDE2 (Figure 4A) show that in compound (***+***)-**11h**, the oxygen atom of the 5-position methoxyl group on the benzene ring connected to the pyran ring can form a hydrogen bond with Y827, which explains its improved activity compared with methyl substitution. However, bulky residues such as Y827 and M847 may cause steric hindrance at this position, so when the methyl group is replaced by tert-butyl and other larger groups, the activity of the compound decreases. The side chain at position 2 on the benzene ring can enter the H pocket and form stronger hydrophobic interactions with L770/H773/T805/L809/F862/I870, thus enhancing the inhibitory activity. Moreover, the size of the H pocket is just large enough to accommodate the benzyl side chain with CF_3_ at position 4. Either an increase or decrease in the size of the side chain length reduces the activity of the compound. The volume of Cl and F is smaller than that of CF_3_ and it does not completely occupy the H pocket, so the activity is not as high as it is with CF_3_. OCF_3_ has an extra oxygen atom, which increases its volume, so its activity also decreases. The superimposed diagram of the docking results of (***R***)-**LZ77** and (***+***)-**11h** (Figure 4B) clearly shows that the side chain at position 2 on the benzene ring connected to the pyran ring enters the H pocket but that the side chain at position 3 does not. In addition, after changing the chirality of the compound, the molecular docking pose (Figure 4C) suggests that the pyranopyrazole skeleton moves away from the pocket and loses its ability to interact with F862 and Q859, so the inhibitory activity of (***−***)-**11h** against PDE2 weakens or disappears.

### 2.5. Decomposition of Binding Free Energies

The decomposition of binding free energies was used to explore the contribution of each residue in the protein to the binding of the inhibitor. Eight-nanosecond (8 ns) molecular dynamics simulations were performed for complexes of ((***R***)-**LZ77**)–PDE2 and ((***+***)-**11h**)–PDE2, and 100 snapshots extracted from trajectories in the last 1 ns were used to calculate the MM-PBSA binding free energies and to perform energy decomposition. The predicted binding free energies were −27.30 and −30.02 kcal/mol for ((***R***)-**LZ77**)–PDE2 and ((***+***)-**11h**)–PDE2, respectively. In general, residues that possess interaction energies lower than -1 kcal/mol are considered important for the recognition of ligands. The energy decomposition results further suggest that such residues mainly contribute to the binding of (***R***)-**LZ77** and (***+***)-**11h** with PDE2: F862 (−3.45 and −4.42 kcal/mol), L770 (−2.58 and −2.22 kcal/mol), Q812 (−1.14 and −1.67 kcal/mol), Q859 (−1.82 and −1.57 kcal/mol), I866 (−1.11 and −1.30 kcal/mol), F830 (−1.45 and −1.22 kcal/mol), and M847 (−1.77 and −1.05 kcal/mol). Among these residues, F862, L770, and Q859 contribute most to the total binding free energies. This is in agreement with the binding patterns, in which both (***R***)-**LZ77** and (***+***)-**11h** form remarkable hydrophobic interactions with F862/L770 and direct hydrogen bonds with Q859 through their dihydropyranopyrazole scaffolds. L809 and F862 had major differences in their energy contributions: their binding free energy contributions to ((***+***)-**11h**)–PDE2 were 1.46 and 0.97 kcal/mol lower than those to ((***R***)-**LZ77**)–PDE2. Since L770, L809, and F862 are involved in the composition of the unique H pocket, the enhanced interaction with L809 and F862 may account for the improved PDE2 inhibition by (***+***)-**11h** relative to (***R***)-**LZ77** (Figure 4D).

## 3. Materials and Methods

### 3.1. Chemistry

Unless otherwise specified, all compounds were purchased from commercial sources (Bide Chemical Co., Ltd., Xiangtan, Hunan, China; Sigma-Aldrich, St. Louis, MO, USA; and J&K Chemical Ltd., Beijing, China), and no further purification was required. ^1^H-NMR and ^13^C-NMR spectra were recorded at room temperature on a Bruker AVANCE 400 or 500 spectrometer (Karlsruhe, Germany) with TMS as an internal reference. Peaks were labeled as single (s), doublet (d), two doublets (dd), doublet of triplets (dt), triplet (t), or multiplet (m). A Shimadzu LCMS-IT-TOF high-resolution mass spectrometer (Kyoto, Japan) was used for molecular weight confirmation. The purity of compounds was confirmed to be over 95% and was determined by reversed-phase high performance liquid chromatography (HPLC) with a TC-C18 column (250 mm × 4.6 mm, 5 µm) using acetonitrile/water (70%, *v*/*v*) as the mobile phase (1.0 mL/min) at 25 °C.

#### 3.1.1. General Procedure for Synthesis of Intermediate Compounds **3a**–**c**

Potassium carbonate (1.5 mmol) was added to a solution of aldehyde (1.0 mmol) with different substituents and benzyl chloride (1.0 mmol) with different substituents or 1-(2-chloroethyl)-4-trifluoromethylbenzene (1.0 mmol) in acetonitrile (15 mL). The mixture was heated to 80 °C for 5 h. After the reaction was complete and the solvent was spin-dried under reduced pressure, the mixture was diluted with water (20 mL) and extracted with ethyl acetate (60 mL), and the organic layer was separated and dried over anhydrous sodium sulfate. After the evaporation of solvent under reduced pressure, the crude product was purified on a silica gel column to produce compounds **3a**–**c** as a white solid.

*3-((4-Chlorobenzyl)oxy)-4-methoxybenzaldehyde* (**3a**). White solid. Yield, 80%. ^1^H-NMR (400 MHz, CDCl_3_) δ 9.82 (s, 1H), 7.48 (dd, *J* = 8.2, 1.9 Hz, 1H), 7.43 (d, *J* = 1.8 Hz, 1H), 7.40 (d, *J* = 8.6 Hz, 2H), 7.37–7.33 (m, 2H), 7.00 (d, *J* = 8.2 Hz, 1H), 5.15 (s, 2H), 3.97 (s, 3H).

*3-Methoxy-5-((4-(trifluoromethyl)benzyl)oxy)benzaldehyde* (**3b**). White solid. Yield, 84%. ^1^H-NMR (400 MHz, CDCl_3_) δ 9.93 (s, 1H), 7.68 (d, *J* = 8.1 Hz, 2H), 7.58 (d, *J* = 8.0 Hz, 2H), 7.12–7.06 (m, 2H), 6.80 (t, *J* = 2.3 Hz, 1H), 5.19 (s, 2H), 3.88 (s, 3H).

*3-((4-Chlorobenzyl)oxy)-5-methoxybenzaldehyde* (**3c**). White solid. Yield, 77%. ^1^H-NMR (400 MHz, CDCl_3_) δ 9.93 (s, 1H), 7.39 (s, 4H), 7.12–7.03 (m, 2H), 6.79 (t, *J* = 2.3 Hz, 1H), 5.09 (s, 2H), 3.87 (s, 3H).

#### 3.1.2. General Procedure for Synthesis of Compounds **4a**–**c**

Aldehyde (1.0 mmol), malononitrile (1.0 mmol), and triethylamine (1.0 mmol) were added to ethanol (10 ml) and reacted at 85 °C for 5 min, and then, pyrazolone was added and reacted at the same temperature for 15 min. After completion, the solid was filtered out, washed with ethanol, and dried in a vacuum drying oven to obtain target compounds **4a**–**c**.

*6-Amino-4-(3-((4-chlorobenzyl)oxy)-4-methoxyphenyl)-3-methyl-1,4-dihydropyrano[2,3-c]pyrazole-5-carbonitrile* (**4a**). White solid. Yield, 61%. ^1^H-NMR (400 MHz, DMSO-*d6*) δ 12.03 (s, 1H), 7.42 (s, 4H), 6.92 (d, *J* = 8.3 Hz, 1H), 6.81 (s, 1H), 6.80 (s, 2H), 6.72 (dd, *J* = 8.3, 2.0 Hz, 1H), 5.06–4.98 (m, 2H), 4.50 (s, 1H), 3.75 (s, 3H), 1.69 (s, 3H). ^13^C-NMR (126 MHz, DMSO-*d6*) δ 161.23, 155.15, 148.51, 147.58, 137.30, 136.61, 136.04, 132.85, 130.13, 128.81, 121.32, 120.70, 113.94, 112.56, 98.12, 69.51, 57.82, 56.03, 36.25, and 10.13. HRMS (ESI) calculated for C_22_H_19_ClN_4_O_3_ [M + H]^+^: 423.1218, found: 423.1222. Purity: 99.66% (by HPLC).

*6-Amino-4-(3-methoxy-5-((4-(trifluoromethyl)benzyl)oxy)phenyl)-3-methyl-1,4-dihydropyrano[2,3-c]pyrazole-5-carbonitrile* (**4b**). White solid. Yield, 42%. ^1^H-NMR (400 MHz, DMSO-*d6*) δ 12.08 (s, 1H), 7.75 (d, *J* = 8.2 Hz, 2H), 7.66 (d, *J* = 8.1 Hz, 2H), 6.86 (s, 2H), 6.50 (t, *J* = 2.1 Hz, 1H), 6.40 (s, 1H), 6.35 (s, 1H), 5.18 (s, 2H), 4.53 (s, 1H), 3.71 (s, 3H), 1.78 (s, 3H). ^13^C-NMR (126 MHz, DMSO-*d6*) δ 161.47, 160.91, 159.65, 155.17, 147.54, 142.36, 136.08, 128.74 (q, *J* = 31.5 Hz), 128.55, 125.75(q, *J* = 4.2 Hz), 124.72 (q, *J* = 272.6 Hz), 121.24, 106.88, 106.70, 99.54, 97.72, 68.75, 57.23, 55.62, 36.79 and 10.21. HRMS (ESI) calculated for C_23_H_19_F_3_N_4_O_3_ [M + H]^+^: 457.1482, found: 457.1501. Purity: 99.86% (by HPLC).

*6-Amino-4-(3-((4-chlorobenzyl)oxy)-5-methoxyphenyl)-3-methyl-1,4-dihydropyrano[2,3-c]pyrazole-5-carbonitrile* (**4c**). White solid. Yield, 52%. ^1^H-NMR (400 MHz, DMSO-*d6*) δ 12.08 (s, 1H), 7.48–7.41 (m, 4H), 6.86 (s, 2H), 6.47 (t, *J* = 2.1 Hz, 1H), 6.36 (d, *J* = 10.9 Hz, 2H), 5.05 (s, 2H), 4.53 (s, 1H), 3.71 (s, 3H), 1.80 (s, 3H). ^13^C-NMR (126 MHz, DMSO-*d6*) δ 161.47, 160.88, 159.78, 155.18, 147.48, 136.49, 136.09, 132.85, 130.06, 128.86, 121.25, 106.87, 106.61, 99.49, 97.73, 68.82, 57.25, 55.61, 36.78 and 10.26. HRMS (ESI) calculated for C_22_H_19_ClN_4_O_3_ [M + H]^+^: 423.1225, found: 423.1218. Purity: 99.79% (by HPLC).

#### 3.1.3. General Procedure for Synthesis of Intermediate Compounds **7a**–**d**

The acid (1.0 mmol), HATU (1.0 mmol), and DIPEA (2.0 mmol) were added to anhydrous methylene chloride (10 mL) to react for 1 h, and then, aldehyde (1.1 mmol) was added to react overnight at room temperature. After the reaction was complete, water (30 mL) was added for extraction, and the organic layer was separated and dried with anhydrous sodium sulfate. After evaporation of the solvent under reduced pressure, the crude product was purified on a silica gel column to produce compounds **7a**–**d** as a white solid.

*2-Formyl-4-methoxyphenyl-4-(trifluoromethoxy)benzoate* (**7a**). White solid. Yield, 60%. ^1^H-NMR (400 MHz, CDCl_3_) δ 10.14 (s, 1H), 8.30–8.25 (m, 2H), 7.42 (t, *J* = 1.6 Hz, 1H), 7.37 (d, *J* = 8.2 Hz, 2H), 7.22 (d, *J* = 1.7 Hz, 2H), 3.89 (s, 3H).

*2-Formyl-4-methoxyphenyl-4-(trifluoromethyl)benzoate* (**7b**). White solid. Yield, 61%. ^1^H-NMR (400 MHz, CDCl_3_) δ 10.15 (s, 1H), 8.37 (d, *J* = 8.1 Hz, 2H), 7.83 (d, *J* = 8.3 Hz, 2H), 7.46–7.43 (m, 1H), 7.27–7.24 (m, 2H), 3.92 (s, 3H).

*2-Formyl-4-methoxyphenyl-3-(4-chlorophenyl)propanoate* (**7c**). White solid. Yield, 50%. ^1^H-NMR (400 MHz, CDCl_3_) δ 9.93 (s, 1H), 7.33 (d, *J* = 3.1 Hz, 1H), 7.32–7.27 (m, 2H), 7.20 (d, *J* = 8.4 Hz, 2H), 7.14 (dd, *J* = 8.9, 3.2 Hz, 1H), 7.00 (d, *J* = 8.9 Hz, 1H), 3.85 (s, 3H), 3.07 (t, *J* = 7.4 Hz, 2H), 2.95 (t, *J* = 7.4 Hz, 2H).

*2-Formyl-4-methoxyphenyl-4-(4-chlorophenyl)butanoate* (**7d**). White solid. Yield, 47%. ^1^H-NMR (400 MHz, CDCl_3_) δ 10.05 (s, 1H), 7.35 (d, *J* = 3.1 Hz, 1H), 7.28 (d, *J* = 8.4 Hz, 2H), 7.15 (dt, *J* = 6.9, 3.6 Hz, 3H), 7.05 (d, *J* = 8.9 Hz, 1H), 3.85 (s, 3H), 2.73 (t, *J* = 7.6 Hz, 2H), 2.65 (t, *J* = 7.4 Hz, 2H), 2.13–2.02 (m, 2H).

#### 3.1.4. General Procedure for Synthesis of Compounds **8a**–**d**

Aldehyde (1.0 mmol), malononitrile (1.0 mmol), and *N*-methylmorpholine (1.0 mmol) were added to ethanol (10 mL) and reacted at room temperature for 5 min, and then, pyrazolone was added and reacted at the same temperature overnight. After completion, the solvent was spin-dried under reduced pressure; then, the mixture was diluted with water (20 mL) and extracted with dichloromethane (60 mL), and the organic layer was separated and dried over anhydrous sodium sulfate. After evaporation of the solvent under reduced pressure, the crude product was purified on a silica gel column to yield compounds **8a**–**d.**

*2-(6-Amino-5-cyano-3-methyl-1,4-dihydropyrano[2,3-c]pyrazol-4-yl)-4-methoxyphenyl-4-(trifluoromethoxy)benzoate* (**8a**). White solid. Yield, 47%. ^1^H-NMR (400 MHz, DMSO-*d6*) δ 11.98 (s, 1H), 8.09 (d, *J* = 8.0 Hz, 2H), 7.53 (d, *J* = 8.4 Hz, 2H), 7.13 (d, *J* = 8.8 Hz, 1H), 6.93 (dd, *J* = 8.9, 3.0 Hz, 1H), 6.79 (s, 3H), 4.69 (s, 1H), 3.76 (s, 3H), 1.78 (s, 3H). ^13^C-NMR (126 MHz, DMSO-*d6*) δ 163.69, 161.25, 157.41, 155.13, 152.47, 142.35, 136.54, 136.13, 132.53, 128.20, 124.93, 121.12, 121.01, 120.39 (q, *J* = 258.3 Hz), 113.15, 96.36, 56.30, 55.89 and 10.09. HRMS (ESI) calculated for C_23_H_17_F_3_N_4_O_5_ [M + H]^+^: 487.1224, found: 487.1240. Purity: 99.38% (by HPLC).

*2-(6-Amino-5-cyano-3-methyl-1,4-dihydropyrano[2,3-c]pyrazol-4-yl)-4-methoxyphenyl-4-(trifluoromethyl)benzoate* (**8b**). White solid. Yield, 47%. ^1^H-NMR (400 MHz, DMSO-*d6*) δ 11.97 (s, 1H), 8.13 (d, *J* = 7.3 Hz, 2H), 7.92 (d, *J* = 8.2 Hz, 2H), 7.15 (d, *J* = 9.0 Hz, 1H), 6.94 (dd, *J* = 8.8, 2.9 Hz, 1H), 6.87 (s, 1H), 6.80 (s, 2H), 4.69 (s, 1H), 3.77 (s, 3H), 1.78 (s, 3H). ^13^C-NMR (126 MHz, DMSO-*d6*) δ 163.68, 161.22, 157.41, 155.02, 142.38, 136.19, 133.54 (q, *J* = 31.9 Hz), 132.92, 130.85, 126.11 (q, *J* = 2.5 Hz), 125.03, 124.24 (q, *J* = 273.0 Hz), 121.03, 115.92, 113.18, 96.27, 56.21, 55.90 and 10.09. HRMS (ESI) calculated for C_23_H_17_F_3_N_4_O_4_[M + H]^+^: 471.1275, found: 471.1267. Purity: 96.99% (by HPLC).

*2-(6-Amino-5-cyano-3-methyl-1,4-dihydropyrano[2,3-c]pyrazol-4-yl)-4-methoxyphenyl-3-(4-chlorophenyl)propanoate* (**8c**). White solid. Yield, 31%. ^1^H-NMR (400 MHz, DMSO-*d6*) δ 12.04 (s, 1H), 7.32 (q, *J* = 8.5 Hz, 4H), 6.87–6.85 (m, 2H), 6.85 (s, 2H), 6.78 (s, 1H), 4.51 (s, 1H), 3.73 (s, 3H), 2.87–2.64 (m, 4H), 1.73 (s, 3H). ^13^C-NMR (126 MHz, DMSO-*d6*) δ 171.16, 161.33, 157.07, 155.33, 142.33, 139.79, 136.36, 136.27, 131.28, 130.69, 128.75, 124.83, 120.95, 115.71, 112.90, 96.66, 56.40, 55.83, 34.90, 29.83 and 10.04. HRMS (ESI) calculated for C_24_H_21_ClN_4_O_4_[M + H]^+^: 465.1324, found: 465.1328. Purity: 99.55% (by HPLC).

*2-(6-Amino-5-cyano-3-methyl-1,4-dihydropyrano[2,3-c]pyrazol-4-yl)-4-methoxyphenyl-4-(4-chlorophenyl)butanoate* (**8d**). White solid. Yield, 33%. ^1^H-NMR (400 MHz, DMSO-*d6*) δ 12.03 (s, 1H), 7.34 (d, *J* = 8.3 Hz, 2H), 7.25 (d, *J* = 8.3 Hz, 2H), 6.94 (d, *J* = 8.8 Hz, 1H), 6.88–6.83 (m, 3H), 6.78 (s, 1H), 4.62 (s, 1H), 3.73 (s, 3H), 2.62 (t, *J* = 7.8 Hz, 2H), 2.46–2.30 (m, 2H), 1.87–1.77 (m, 2H), 1.75 (s, 3H).^13^C-NMR (101 MHz, DMSO-*d6*) δ 171.74, 161.37, 157.05, 155.30, 142.37, 140.95, 136.39, 136.22, 130.95, 130.68, 128.72, 124.92, 120.97, 115.64, 112.89, 96.68, 56.31, 55.82, 34.08, 32.92, 26.24 and 10.05. HRMS (ESI) calculated for C_25_H_23_ClN_4_O_4_ [M + H]^+^: 479.1481, found: 479.1472. Purity: 99.09% (by HPLC).

#### 3.1.5. General Procedure for Synthesis of Intermediate Compounds **10a**–**q** and **10t**

The synthesis method was the same as that used for **3a**–**c**.

*4-Methoxy-2-((4-methoxybenzyl)oxy)benzaldehyde* (**10a**). White solid. Yield, 82%. ^1^H-NMR (400 MHz, DMSO) δ 10.20 (s, 1H), 7.67 (d, *J* = 8.7 Hz, 1H), 7.44 (d, *J* = 8.6 Hz, 2H), 6.99–6.95 (m, 2H), 6.81 (d, *J* = 2.2 Hz, 1H), 6.65 (dd, *J* = 8.7, 1.8 Hz, 1H), 5.20 (s, 2H), 3.86 (s, 3H), 3.76 (s, 3H).

*2-(Benzyloxy)-4-methoxybenzaldehyde* (**10b**). White solid. Yield, 80%. ^1^H-NMR (400 MHz, CDCl_3_) δ 10.41 (s, 1H), 7.87 (d, *J* = 8.7 Hz, 1H), 7.48–7.37 (m, 5H), 6.59 (dd, *J* = 8.7, 1.8 Hz, 1H), 6.54 (d, *J* = 2.2 Hz, 1H), 5.19 (s, 2H), 3.88 (s, 3H).

*2-((4-Chlorobenzyl)oxy)-4-methoxybenzaldehyde* (**10c**). White solid. Yield, 83%. ^1^H-NMR (400 MHz, CDCl_3_) δ 10.38 (s, 1H), 7.87 (d, *J* = 8.7 Hz, 1H), 7.40 (s, 4H), 6.60 (dd, *J* = 8.7, 1.7 Hz, 1H), 6.50 (d, *J* = 2.2 Hz, 1H), 5.15 (s, 2H), 3.88 (s, 3H).

*2-(Benzyloxy)-5-methoxybenzaldehyde* (**10d**). White solid. Yield, 86%. ^1^H-NMR (400 MHz, CDCl_3_) δ 10.53 (s, 1H), 7.47–7.34 (m, 6H), 7.14 (dd, *J* = 9.0, 3.3 Hz, 1H), 7.03 (d, *J* = 9.1 Hz, 1H), 5.18 (s, 2H), 3.83 (s, 3H).

*5-Methoxy-2-((4-methoxybenzyl)oxy)benzaldehyde* (**10e**). White solid. Yield, 79%.^1^H-NMR (400 MHz, DMSO) δ 10.33 (s, 1H), 7.41 (d, *J* = 8.6 Hz, 2H), 7.30 (d, *J* = 9.1 Hz, 1H), 7.25 (dd, *J* = 9.1, 3.2 Hz, 1H), 7.17 (d, *J* = 3.1 Hz, 1H), 6.97–6.93 (m, 2H), 5.16 (s, 2H), 3.76 (s, 3H), 3.75 (s, 3H).

*2-((4-Chlorobenzyl)oxy)-5-methoxybenzaldehyde* (**10f**). White solid. Yield, 81%. ^1^H-NMR (400 MHz, CDCl_3_) δ 10.51 (s, 1H), 7.42–7.38 (m, 4H), 7.37 (d, *J* = 3.2 Hz, 1H), 7.14 (dd, *J* = 9.0, 3.2 Hz, 1H), 6.99 (d, *J* = 9.1 Hz, 1H), 5.14 (s, 2H), 3.83 (s, 3H).

*2-((4-Fluorobenzyl)oxy)-5-methoxybenzaldehyde* (**10g**). White solid. Yield, 83%. ^1^H-NMR (400 MHz, CDCl_3_) δ 10.50 (s, 1H), 7.45–7.39 (m, 2H), 7.37 (d, *J* = 3.2 Hz, 1H), 7.14 (dd, *J* = 8.3, 2.5 Hz, 1H), 7.12–7.08 (m, 2H), 7.01 (d, *J* = 9.1 Hz, 1H), 5.13 (s, 2H), 3.83 (s, 3H).

*5-Methoxy-2-((4-(trifluoromethyl)benzyl)oxy)benzaldehyde* (**10h**). White solid. Yield, 82%. ^1^H-NMR (400 MHz, CDCl_3_) δ 10.55 (s, 1H), 7.69 (d, *J* = 8.1 Hz, 2H), 7.58 (d, *J* = 8.0 Hz, 2H), 7.39 (d, *J* = 3.2 Hz, 1H), 7.14 (dd, *J* = 9.0, 3.2 Hz, 1H), 6.99 (d, *J* = 9.0 Hz, 1H), 5.24 (s, 2H), 3.84 (s, 3H).

*5-Methoxy-2-((4-(trifluoromethoxy)benzyl)oxy)benzaldehyde* (**10i**). White solid. Yield, 76%. ^1^H-NMR (400 MHz, CDCl_3_) δ 10.50 (s, 1H), 7.46 (d, *J* = 8.7 Hz, 2H), 7.35 (d, *J* = 3.2 Hz, 1H), 7.25 (d, *J* = 7.4 Hz, 2H), 7.12 (dd, *J* = 9.0, 3.3 Hz, 1H), 6.98 (d, *J* = 9.1 Hz, 1H), 5.15 (s, 2H), 3.81 (s, 3H).

*5-(Tert-butyl)-2-((4-(trifluoromethyl)benzyl)oxy)benzaldehyde* (**10j**). White solid. Yield, 87%. ^1^H-NMR (400 MHz, CDCl_3_) δ 10.59 (s, 1H), 7.92 (d, *J* = 2.6 Hz, 1H), 7.69 (d, *J* = 8.1 Hz, 2H), 7.62–7.56 (m, 3H), 6.97 (d, *J* = 8.7 Hz, 1H), 5.27 (s, 2H), 1.34 (s, 9H).

*5-Methyl-2-((4-(trifluoromethyl)benzyl)oxy)benzaldehyde* (**10k**). White solid. Yield, 89%. ^1^H-NMR (400 MHz, CDCl_3_) δ 10.56 (s, 1H), 7.69 (d, *J* = 7.8 Hz, 3H), 7.58 (d, *J* = 8.1 Hz, 2H), 7.36 (dd, *J* = 8.5, 2.1 Hz, 1H), 6.93 (d, *J* = 8.5 Hz, 1H), 5.25 (s, 2H), 2.35 (s, 3H).

*2,5-Bis((4-(trifluoromethyl)benzyl)oxy)benzaldehyde* (**10l**). White solid. Yield, 79%. ^1^H-NMR (400 MHz, CDCl_3_) δ 10.54 (s, 1H), 7.72–7.64 (m, 4H), 7.60–7.55 (m, 4H), 7.46 (d, *J* = 3.2 Hz, 1H), 7.21 (dd, *J* = 9.0, 3.3 Hz, 1H), 7.01 (d, *J* = 9.0 Hz, 1H), 5.24 (s, 2H), 5.14 (s, 2H).

*5-Hydroxy-2-((4-(trifluoromethyl)benzyl)oxy)benzaldehyde* (**10m**). White solid. Yield, 56%. ^1^H-NMR (400 MHz, CDCl_3_) δ 10.51 (s, 1H), 7.69 (d, *J* = 8.2 Hz, 2H), 7.57 (d, *J* = 8.1 Hz, 2H), 7.37 (d, *J* = 3.2 Hz, 1H), 7.11 (dd, *J* = 8.9, 3.2 Hz, 1H), 6.96 (d, *J* = 8.9 Hz, 1H), 5.23 (s, 2H).

*5-Methoxy-2-(4-(trifluoromethyl)phenethoxy)benzaldehyde* (**10n**). White solid. Yield, 69%. ^1^H-NMR (400 MHz, CDCl_3_) δ 10.40 (s, 1H), 7.61 (d, *J* = 8.0 Hz, 2H), 7.42 (d, *J* = 8.0 Hz, 2H), 7.34 (d, *J* = 3.3 Hz, 1H), 7.13 (dd, *J* = 9.0, 3.3 Hz, 1H), 6.93 (d, *J* = 9.1 Hz, 1H), 4.30 (t, *J* = 6.5 Hz, 2H), 3.82 (s, 3H), 3.22 (t, *J* = 6.5 Hz, 2H).

*5-Methoxy-2-(3-(4-(trifluoromethyl)phenyl)propoxy)benzaldehyde* (**10o**). White solid. Yield, 78%. ^1^H-NMR (400 MHz, CDCl_3_) δ 10.51 (s, 1H), 7.58 (d, *J* = 8.1 Hz, 2H), 7.36 (d, *J* = 3.3 Hz, 1H), 7.34 (d, *J* = 8.0 Hz, 2H), 7.13 (dd, *J* = 9.0, 3.3 Hz, 1H), 6.92 (d, *J* = 9.1 Hz, 1H), 4.07 (t, *J* = 6.1 Hz, 2H), 3.83 (s, 3H), 2.92 (t, *J* = 7.6 Hz, 2H), 2.25–2.14 (m, 2H).

*5-(Trifluoromethoxy)-2-((4-(trifluoromethyl)benzyl)oxy)benzaldehyde* (**10p**). White solid. Yield, 87%. ^1^H-NMR (400 MHz, CDCl_3_) δ 10.54 (s, 1H), 7.76 (d, *J* = 2.4 Hz, 1H), 7.71 (d, *J* = 8.2 Hz, 2H), 7.59 (d, *J* = 8.0 Hz, 2H), 7.42 (dd, *J* = 9.1, 2.6 Hz, 1H), 7.06 (d, *J* = 9.1 Hz, 1H), 5.29 (s, 2H).

*5-(Difluoromethoxy)-2-((4-(trifluoromethyl)benzyl)oxy)benzaldehyde* (**10q**). Diethyl bromo difluoromethylphosphonate (2 mmol) was added in one portion to a solution of 14 m (1 mmol) and KOH (20 mmol) in CH_3_CN–H_2_O (10 mL, 1:1) at 0 °C, and the reaction mixture was allowed to warm to room tempeature. After 20 min, the reaction mixture was diluted with ethyl acetate (10 mL), and the organic phase was separated. Then, the water phase was washed with another 10 mL of ethyl acetate, and the combined organic fractions were dried over anhydrous Na_2_SO_4_. Evaporation of the solvent yielded a crude product that was purified on a silica gel column to obtain compound **10q**. White solid. Yield, 82%. ^1^H-NMR (400 MHz, CDCl_3_) δ 10.36 (s, 1H), 7.68 (d, *J* = 8.2 Hz, 2H), 7.57 (d, *J* = 8.1 Hz, 2H), 7.49 (d, *J* = 1.3 Hz, 1H), 7.27–7.23 (m, 2H), 6.62 (t, *J* = 72.9 Hz, 1H), 5.18 (s, 2H). ^19^F-NMR (376 MHz, CDCl_3_) δ −62.65 (s), −81.41 (d, *J* = 72.9 Hz).

*4-Chloro-5-methoxy-2-((4-(trifluoromethyl)benzyl)oxy)benzaldehyde* (**10t**). The synthesis method was the same as that used for **3a**–**c**. White solid. Yield, 67%. ^1^H-NMR (400 MHz, CDCl_3_) δ 10.48 (s, 1H), 7.71 (d, *J* = 8.1 Hz, 2H), 7.58 (d, *J* = 8.0 Hz, 2H), 7.44 (s, 1H), 7.14 (s, 1H), 5.22 (s, 2H), 3.94 (s, 3H).

#### 3.1.6. General Procedure for Synthesis of Intermediate Compounds **11a**–**t**

The synthesis method was the same as that used for **4a**–**c**.

*6-Amino-4-(4-methoxy-2-((4-methoxybenzyl)oxy)phenyl)-3-methyl-1,4-dihydropyrano[2,3-c]pyrazole-5-carbonitrile* (**11a**). White solid. Yield, 41%. ^1^H-NMR (500 MHz, DMSO-*d6*) δ 11.98 (s, 1H), 7.33 (d, *J* = 7.3 Hz, 2H), 6.93 (d, *J* = 7.6 Hz, 2H), 6.89 (d, *J* = 8.0 Hz, 1H), 6.72 (s, 2H), 6.63 (s, 1H), 6.48 (d, *J* = 8.3 Hz, 1H), 5.03 (q, *J* = 11.8 Hz, 2H), 4.89 (s, 1H), 3.75 (s, 3H), 3.72 (s, 3H), 1.74 (s, 3H). ^13^C-NMR (126 MHz, DMSO-*d6*) δ 161.59, 159.45, 159.33, 156.75, 155.56, 135.49, 130.07, 129.66, 129.41, 124.97, 121.47, 114.26, 105.98, 100.07, 98.41, 69.75, 57.35, 55.55, 55.51 and 10.05. HRMS (ESI) calculated for C_23_H_22_N_4_O_4_ [M + H]^+^: 419.1714, found: 419.1708. Purity: 99.77% (by HPLC).

*6-Amino-4-(2-(benzyloxy)-4-methoxyphenyl)-3-methyl-1,4-dihydropyrano [2,3-c] pyrazole-5-carbonitrile* (**11b**). White solid. Yield, 36%. ^1^H-NMR (500 MHz, DMSO-*d6*) δ 11.99 (s, 1H), 7.44–7.35 (m, 4H), 7.31 (t, *J* = 6.8 Hz, 1H), 6.91 (d, *J* = 8.3 Hz, 1H), 6.74 (s, 2H), 6.62 (s, 1H), 6.49 (d, *J* = 8.4 Hz, 1H), 5.12 (q, *J* = 12.6 Hz, 2H), 4.94 (s, 1H), 3.72 (s, 3H), 1.75 (s, 3H). ^13^C-NMR (126 MHz, DMSO-*d6*) δ 161.59, 159.48, 156.68, 155.57, 137.60, 135.51, 130.15, 128.89, 128.15, 127.83, 124.97, 121.46, 106.07, 100.04, 98.40, 69.93, 57.36, 55.56 and 10.05. HRMS (ESI) calculated for C_22_H_20_N_4_O_3_ [M + H]^+^: 389.1608, found: 389.1598. Purity: 99.01% (by HPLC).

*6-Amino-4-(2-((4-chlorobenzyl)oxy)-4-methoxyphenyl)-3-methyl-1,4-dihydropyrano[2,3-c]pyrazole-5-carbonitrile* (**11c**). white solid. Yield, 52%. ^1^H-NMR (400 MHz, DMSO-*d6*) δ 11.97 (s, 1H), 7.44 (m, *J* = 8.2 Hz, 4H), 6.93 (d, *J* = 8.3 Hz, 1H), 6.72 (s, 2H), 6.62 (s, 1H), 6.50 (d, *J* = 8.1 Hz, 1H), 5.11 (q, *J* = 12.6 Hz, 2H), 4.90 (s, 1H), 3.73 (s, 3H), 1.75 (s, 3H). ^13^C-NMR (126 MHz, DMSO-*d6*) δ 161.54, 159.52, 156.53, 155.57, 136.60, 135.49, 132.68, 130.27, 129.68, 128.84, 124.83, 121.47, 106.04, 100.12, 98.29, 69.02, 57.28, 55.57 and 10.05. HRMS (ESI) calculated for C_22_H_19_ClN_4_O_3_ [M + H]^+^: 423.1218, found: 423.1219. Purity: 99.91% (by HPLC).

*6-Amino-4-(2-(benzyloxy)-5-methoxyphenyl)-3-methyl-1,4-dihydropyrano [2,3-c] pyrazole-5-carbonitrile* (**11d**). White solid. Yield, 44%. ^1^H-NMR (500 MHz, DMSO-*d6*) δ 12.03 (s, 1H), 7.43–7.34 (m, 4H), 7.31 (t, *J* = 6.9 Hz, 1H), 7.01 (d, *J* = 8.8 Hz, 1H), 6.81 (s, 2H), 6.76 (d, *J* = 8.9 Hz, 1H), 6.53 (s, 1H), 5.05 (q, *J* = 12.3 Hz, 2H), 4.99 (s, 1H), 3.64 (s, 3H), 1.77 (s, 3H). ^13^C-NMR (126 MHz, DMSO-*d6*) δ 161.82, 155.52, 153.96, 150.05, 137.86, 135.62, 134.07, 128.85, 128.09, 127.84, 121.37, 115.61, 114.39, 112.34, 98.00, 70.69, 56.81, 55.69 and 10.04. HRMS (ESI) calculated for C_22_H_20_N_4_O_3_ [M + H]^+^: 389.1608, found: 389.1601. Purity: 99.93% (by HPLC).

*6-Amino-4-(5-methoxy-2-((4-methoxybenzyl)oxy)phenyl)-3-methyl-1,4-dihydropyrano[2,3-c]pyrazole-5-carbonitrile* (**11e**). White solid. Yield, 48%. ^1^H-NMR (400 MHz, DMSO-*d6*) δ 12.01 (s, 1H), 7.33 (d, *J* = 8.5 Hz, 2H), 7.01 (d, *J* = 9.0 Hz, 1H), 6.93 (d, *J* = 8.7 Hz, 2H), 6.79 (s, 2H), 6.76 (dd, *J* = 8.9, 3.1 Hz, 1H), 6.52 (d, *J* = 2.9 Hz, 1H), 4.97 (m, 3H), 3.76 (s, 3H), 3.65 (s, 3H), 1.76 (s, 3H). ^13^C-NMR (126 MHz, DMSO-*d6*) δ 161.81, 159.30, 155.51, 153.89, 150.12, 135.61, 134.09, 129.69, 126.66, 121.37, 115.55, 114.53, 114.23, 112.32, 98.01, 70.54, 56.82, 55.68, 55.52 and 10.04. HRMS (ESI) calculated for C_23_H_22_N_4_O_4_ [M + H]^+^: 419.1714, found: 419.1723. Purity: 99.35% (by HPLC).

*6-Amino-4-(2-((4-chlorobenzyl)oxy)-5-methoxyphenyl)-3-methyl-1,4-dihydropyrano[2,3-c]pyrazole-5-carbonitrile* (**11f**). White solid. Yield, 48%. ^1^H-NMR (400 MHz, DMSO-*d6*) δ 12.01 (s, 1H), 7.47–7.37 (m, 4H), 7.00 (d, *J* = 9.0 Hz, 1H), 6.80 (s, 2H), 6.77 (dd, *J* = 9.0, 3.1 Hz, 1H), 6.56 (d, *J* = 2.6 Hz, 1H), 5.04 (q, *J* = 12.4 Hz, 2H), 4.96 (s, 1H), 3.65 (s, 3H), 1.77 (s, 3H). ^13^C-NMR (126 MHz, DMSO-*d6*) δ 161.78, 155.53, 153.97, 149.89, 136.86, 135.63, 133.95, 132.63, 129.69, 128.82, 121.39, 115.76, 114.42, 112.36, 97.91, 69.75, 56.76, 55.70 and 10.04. HRMS (ESI) calculated for C_22_H_19_ClN_4_O_3_ [M + H]^+^: 423.1218, found: 423.1229. Purity: 99.91% (by HPLC).

*6-Amino-4-(2-((4-fluorobenzyl)oxy)-5-methoxyphenyl)-3-methyl-1,4-dihydropyrano[2,3-c]pyrazole-5-carbonitrile* (**11g**). White solid. Yield, 54%. ^1^H-NMR (400 MHz, DMSO-*d6*) δ 12.02 (s, 1H), 7.47–7.40 (m, 2H), 7.19 (t, *J* = 8.8 Hz, 2H), 7.02 (d, *J* = 8.9 Hz, 1H), 6.80–6.75 (m, 3H), 6.55 (d, *J* = 2.8 Hz, 1H), 5.02 (q, *J* = 12.0 Hz, 2H), 4.96 (s, 1H), 3.65 (s, 3H), 1.76 (s, 3H). ^13^C-NMR (101 MHz, DMSO-*d6*) δ 162.10 (d, *J* = 244.4 Hz), 161.78, 155.53, 153.96, 149.97, 135.62, 134.02, 134.00, 130.13, 130.05, 121.40, 115.70, 115.63 (d, *J* = 21.4 Hz), 114.46, 112.34, 97.93, 69.94, 56.78, 55.68 and 10.04. HRMS (ESI) calculated for C_22_H_19_FN_4_O_3_ [M + H]^+^: 407.1514, found: 407.1521. Purity: 99.85% (by HPLC).

*6-Amino-4-(5-methoxy-2-((4-(trifluoromethyl)benzyl)oxy)phenyl)-3-methyl-1,4-dihydropyrano[2,3-c]pyrazole-5-carbonitrile* (**11h**). White solid. Yield, 50%. ^1^H-NMR (400 MHz, DMSO-*d6*) δ 12.02 (s, 1H), 7.74 (d, *J* = 8.1 Hz, 2H), 7.60 (d, *J* = 7.8 Hz, 2H), 7.01 (d, *J* = 9.0 Hz, 1H), 6.81 (s, 2H), 6.78 (dd, *J* = 8.9, 3.1 Hz, 1H), 6.59 (s, 1H), 5.16 (q, *J* = 13.2 Hz, 2H), 4.99 (s, 1H), 3.66 (s, 3H), 1.77 (s, 3H). ^13^C-NMR (126 MHz, DMSO-*d6*) δ 161.78, 155.55, 154.03, 149.78, 142.74, 135.65, 133.91, 128.57 (q, *J* = 39.9 Hz), 128.18, 125.70 (q, *J* = 3.8 Hz), 124.75 (q, *J* = 253.7 Hz), 121.39, 115.87, 114.34, 112.39, 97.88, 69.62, 56.74, 55.70 and 10.04. HRMS (ESI) calculated for C_23_H_19_F_3_N_4_O_3_ [M + H]^+^: 457.1482, found: 457.1496. Purity: 99.96% (by HPLC).

*6-Amino-4-(5-methoxy-2-((4-(trifluoromethoxy)benzyl)oxy)phenyl)-3-methyl-1,4-dihydropyrano[2,3-c]pyrazole-5-carbonitrile* (**11i**). White solid. Yield, 47%. ^1^H-NMR (400 MHz, DMSO-*d6*) δ 12.02 (s, 1H), 7.52 (d, *J* = 8.2 Hz, 2H), 7.37 (d, *J* = 8.2 Hz, 2H), 7.02 (d, *J* = 8.9 Hz, 1H), 6.80 (s, 2H), 6.78 (dd, *J* = 9.0, 3.2 Hz, 1H), 6.56 (d, *J* = 2.2 Hz, 1H), 5.09 (q, *J* = 12.6 Hz, 2H), 4.98 (s, 1H), 3.65 (s, 3H), 1.76 (s, 3H). ^13^C-NMR (126 MHz, DMSO-*d6*) δ 161.77, 155.55, 154.01, 149.85, 148.17, 137.35, 135.64, 133.98, 129.66, 121.42, 121.39, 120.56 (q, *J* = 256.6 Hz), 115.75, 114.39, 112.37, 97.90, 69.63, 56.78, 55.69 and 10.02. HRMS (ESI) calculated for C_23_H_19_F_3_N_4_O_4_ [M + H]^+^: 473.1431, found: 473.1444. Purity: 99.82% (by HPLC).

*6-Amino-4-(5-(tert-butyl)-2-((4-(trifluoromethyl)benzyl)oxy)phenyl)-3-methyl-1,4-dihydropyrano[2,3-c]pyrazole-5-carbonitrile* (**11j**). White solid. Yield, 36%. ^1^H-NMR (400 MHz, DMSO-*d6*) δ 11.99 (s, 1H), 7.73 (d, *J* = 8.1 Hz, 2H), 7.57 (d, *J* = 7.9 Hz, 2H), 7.19 (dd, *J* = 8.5, 2.4 Hz, 1H), 7.11 (s, 1H), 6.95 (d, *J* = 8.6 Hz, 1H), 6.77 (s, 2H), 5.18 (q, *J* = 13.4 Hz, 2H), 4.97 (s, 1H), 1.74 (s, 3H), 1.21 (s, 9H). ^13^C-NMR (126 MHz, DMSO-*d6*) δ 161.78, 155.69, 153.66, 143.39, 142.73, 135.55, 131.42, 128.54 (q, *J* = 31.5 Hz), 128.07, 126.54, 125.69 (q, *J* = 3.8 Hz), 124.92, 124.35 (q, *J* = 252.4 Hz), 121.51, 112.54, 98.01, 68.87, 56.90, 34.18, 31.71 and 10.02. HRMS (ESI) calculated for C26H25F3N4O2 [M + H]^+^: 483.2002, found: 483.1984. Purity: 99.84% (by HPLC).

*6-Amino-3-methyl-4-(5-methyl-2-((4-(trifluoromethyl)benzyl)oxy)phenyl)-1,4-dihydropyrano[2,3-c]pyrazole-5-carbonitrile* (**11k**). Yellow solid. Yield, 40%. ^1^H-NMR (400 MHz, DMSO-*d6*) δ 12.01 (s, 1H), 7.74 (d, *J* = 8.2 Hz, 2H), 7.61 (d, *J* = 7.8 Hz, 2H), 7.02–6.93 (m, 2H), 6.84 (s, 1H), 6.79 (s, 2H), 5.20 (q, *J* = 13.3 Hz, 2H), 5.01 (s, 1H), 2.19 (s, 3H), 1.76 (s, 3H). ^13^C-NMR (101 MHz, DMSO-*d6*) δ 161.72, 155.58, 153.50, 142.67, 135.61, 132.36, 130.29, 130.05, 128.81, 128.58 (q, *J* = 31.6 Hz), 128.16, 125.72 (q, *J* = 3.7 Hz), 124.24 (q, *J* = 249.1 Hz), 121.47, 113.05, 98.12, 69.08, 56.99, 20.67 and 10.04. HRMS (ESI) calculated for C_23_H_19_F_3_N_4_O_2_ [M + H]^+^: 441.1533, found: 441.1538. Purity: 99.80% (by HPLC).

*6-Amino-4-(2,5-bis((4-(trifluoromethyl)benzyl)oxy)phenyl)-3-methyl-1,4-dihydropyrano[2,3-c]pyrazole-5-carbonitrile* (**11l**). White solid. Yield, 31%. ^1^H-NMR (400 MHz, DMSO-*d6*) δ 12.03 (s, 1H), 7.76–7.71 (m, 4H), 7.63 (d, *J* = 8.1 Hz, 2H), 7.60 (d, *J* = 7.4 Hz, 2H), 7.02 (d, *J* = 8.9 Hz, 1H), 6.87 (dd, *J* = 9.0, 3.0 Hz, 1H), 6.82 (s, 2H), 6.70 (s, 1H), 5.23–5.13 (m, 2H), 5.11 (s, 2H), 4.99 (s, 1H), 1.71 (s, 3H). ^13^C-NMR (126 MHz, DMSO-*d6*) δ 161.78, 155.57, 152.74, 150.10, 142.67, 142.51, 135.64, 128.70 (q, *J* = 32.0 Hz), 128.59 (q, *J* = 30.7 Hz), 128.55, 128.20, 125.70 (q, *J* = 3.8 Hz), 124.74 (q, *J* = 272.2 Hz), 124.71 (q, *J* = 272.6 Hz), 121.40, 116.82, 114.76, 114.33, 113.66, 97.78, 69.58, 69.05, 56.70 and 9.97. HRMS (ESI) calculated for C_30_H_22_F_6_N_4_O_3_ [M + H]^+^: 601.1669, found: 601.1690. Purity: 99.77% (by HPLC).

*6-Amino-4-(5-hydroxy-2-((4-(trifluoromethyl)benzyl)oxy)phenyl)-3-methyl-1,4-dihydropyrano[2,3-c]pyrazole-5-carbonitrile* (**11m**). Yellow solid. Yield, 43%. ^1^H-NMR (400 MHz, DMSO-*d6*) δ 12.03 (s, 1H), 8.92 (s, 1H), 7.75 (d, *J* = 8.2 Hz, 2H), 7.64 (d, *J* = 7.7 Hz, 2H), 6.90 (d, *J* = 8.8 Hz, 1H), 6.81 (s, 2H), 6.56 (dd, *J* = 8.8, 2.9 Hz, 1H), 6.45 (d, *J* = 2.7 Hz, 1H), 5.18–5.08 (m, 2H), 4.99 (s, 1H), 1.78 (s, 3H). ^13^C-NMR (126 MHz, DMSO-*d6*) δ 161.70, 155.50, 152.15, 148.48, 142.94, 135.66, 133.94, 128.56 (q, *J* = 38.2 Hz), 128.20, 125.73 (q, *J* = 3.4 Hz), 124.76 (q, *J* = 248.2 Hz), 121.43, 116.07, 114.73, 114.66, 98.21, 69.95, 57.06 and 10.02. HRMS (ESI) calculated for C_22_H_17_F_3_N_4_O_3_ [M + H]^+^: 443.1326, found: 443.1319. Purity: 99.40% (by HPLC).

*6-Amino-4-(5-methoxy-2-(4-(trifluoromethyl)phenethoxy)phenyl)-3-methyl-1,4-dihydropyrano[2,3-c]pyrazole-5-carbonitrile* (**11n**). White solid. Yield, 37%. ^1^H-NMR (400 MHz, DMSO-*d6*) δ 11.96 (s, 1H), 7.65 (d, *J* = 8.1 Hz, 2H), 7.54 (d, *J* = 8.1 Hz, 2H), 6.95 (d, *J* = 8.9 Hz, 1H), 6.81 (s, 2H), 6.75 (dd, *J* = 8.9, 3.1 Hz, 1H), 6.54 (s, 1H), 4.80 (s, 1H), 4.18–4.06 (m, 2H), 3.64 (s, 3H), 3.09 (t, *J* = 6.4 Hz, 2H), 1.63 (s, 3H). ^13^C-NMR (126 MHz, DMSO-*d6*) δ 161.86, 155.41, 153.91, 150.28, 144.23, 135.55, 134.09, 130.31, 127.50 (q, *J* = 31.9 Hz), 125.54 (q, *J* = 3.8 Hz), 124.90 (q, *J* = 272.2 Hz), 121.35, 115.58, 114.40, 112.36, 98.06, 69.65, 56.76, 55.68, 35.41 and 9.69. HRMS (ESI) calculated for C_24_H_21_F_3_N_4_O_3_ [M + H]^+^: 471.1639, found: 471.1631. Purity: 98.11% (by HPLC).

*6-Amino-4-(5-methoxy-2-(3-(4-(trifluoromethyl)phenyl)propoxy)phenyl)-3-methyl-1,4-dihydropyrano[2,3-c]pyrazole-5-carbonitrile* (**11o**). White solid. Yield, 48%. ^1^H-NMR (400 MHz, DMSO-*d6*) δ 11.97 (s, 1H), 7.63 (d, *J* = 8.1 Hz, 2H), 7.47 (d, *J* = 8.0 Hz, 2H), 6.87 (d, *J* = 8.9 Hz, 1H), 6.77 (s, 2H), 6.76–6.73 (m, 1H), 6.63 (s, 1H), 4.81 (s, 1H), 3.95–3.73 (m, 2H), 3.66 (s, 3H), 2.77 (t, *J* = 7.5 Hz, 2H), 2.02–1.92 (m, 2H), 1.80 (s, 3H). ^13^C-NMR (126 MHz, DMSO-*d6*) δ 161.82, 155.58, 153.51, 150.74, 147.17, 135.50, 133.40, 129.73, 127.06 (q, *J* = 34.4 Hz), 125.55 (q, *J* = 3.8 Hz), 123.86 (q, *J* = 260.4 Hz), 121.48, 115.95, 113.67, 112.30, 98.01, 67.93, 56.50, 55.69, 31.97, 30.71, 19.01 and 10.01. HRMS (ESI) calculated for C_25_H_23_F_3_N_4_O_3_ [M + H]^+^: 485.1495, found: 485.1788. Purity: 98.23% (by HPLC).

*6-Amino-3-methyl-4-(5-(trifluoromethoxy)-2-((4-(trifluoromethyl) benzyl) oxy) phenyl)-1,4-dihydropyrano[2,3-c]pyrazole-5-carbonitrile* (**11p**)**.** White solid. Yield, 55%. ^1^H-NMR (400 MHz, DMSO-*d6*) δ 12.08 (s, 1H), 7.74 (d, *J* = 8.1 Hz, 2H), 7.58 (d, *J* = 7.7 Hz, 2H), 7.23 (dd, *J* = 8.9, 2.2 Hz, 1H), 7.17 (d, *J* = 9.0 Hz, 1H), 7.05 (s, 1H), 6.89 (s, 2H), 5.26 (q, *J* = 13.2 Hz, 2H), 5.04 (s, 1H), 1.76 (s, 3H). ^13^C-NMR (126 MHz, DMSO-*d6*) δ 161.91, 155.60, 154.64, 142.41, 142.00, 135.71, 134.25, 128.74 (q, *J* = 35.7 Hz), 128.24, 125.78 (q, *J* = 3.4 Hz), 123.08 (q, *J* = 235.6 Hz), 121.60 (q, *J* = 265.0 Hz), 121.24, 121.18, 114.36, 97.15, 69.39, 55.95 and 9.96. HRMS (ESI) calculated for C_23_H_16_F_6_N_4_O_3_ [M + H]^+^: 511.1199, found: 511.1196. Purity: 99.87% (by HPLC).

*6-Amino-4-(5-(difluoromethoxy)-2-((4-(trifluoromethyl)benzyl)oxy)phenyl)-3-methyl-1,4-dihydropyrano[2,3-c]pyrazole-5-carbonitrile* (**11q**)**.** Yellow solid. Yield, 47%. ^1^H-NMR (400 MHz, DMSO-*d6*) δ 12.09 (s, 1H), 7.73 (d, *J* = 8.2 Hz, 2H), 7.64 (d, *J* = 8.2 Hz, 2H), 7.14 (d, *J* = 8.8 Hz, 1H), 6.98 (dd, *J* = 9.0, 3.1 Hz, 1H), 6.88 (s, 2H), 6.75 (s, 1H), 5.16 (s, 2H), 4.87 (s, 1H), 1.73 (s, 3H). ^13^C-NMR (126 MHz, DMSO-*d6*) δ 161.80, 155.84, 155.44, 142.88, 142.09, 137.02, 135.76, 128.80 (q, *J* = 31.9 Hz), 128.63, 125.75 (q, *J* = 3.78 Hz), 124.70 (q, *J* = 273.0 Hz), 120.99, 120.66, 117.30 (t, *J* = 25.8 Hz), 116.60, 114.37, 97.15, 69.05, 56.21 and 9.87. HRMS (ESI) calculated for C_23_H_17_F_5_N_4_O_3_ [M + H]^+^: 493.1294, found: 493.1279. Purity: 95.59% (by HPLC).

*6-Amino-4-(5-methoxy-2-((4-(trifluoromethyl)benzyl)oxy)phenyl)-1,4-dihydropyrano[2,3-c]pyrazole-5-carbonitrile* (**11r**). White solid. Yield, 57%. ^1^H-NMR (400 MHz, DMSO-*d6*) δ 12.30 (s, 1H), 7.76 (d, *J* = 8.2 Hz, 2H), 7.66 (d, *J* = 8.1 Hz, 2H), 7.29 (s, 1H), 7.02 (d, *J* = 8.9 Hz, 1H), 6.94 (s, 2H), 6.78 (dd, *J* = 8.9, 3.1 Hz, 1H), 6.64 (d, *J* = 3.1 Hz, 1H), 5.27–5.17 (m, 2H), 5.07 (s, 1H), 3.67 (s, 3H). ^13^C-NMR (126 MHz, DMSO-*d6*) δ 162.39, 155.21, 153.95, 149.70, 142.76, 134.77, 128.62 (q, *J* = 32.8 Hz), 128.21, 126.58, 125.76 (q, *J* = 3.8 Hz), 124.75 (q, *J* = 272.6 Hz), 121.32, 115.01, 114.04, 112.06, 100.61, 69.52, 55.74, 55.16 and 31.13. HRMS (ESI) calculated for C_22_H_17_F_3_N_4_O_3_ [M + H]^+^: 443.1326, found: 443.1323. Purity: 99.17% (by HPLC).

*6-Amino-4-(5-methoxy-2-((4-(trifluoromethyl)benzyl)oxy)phenyl)-3-phenyl-1,4-dihydropyrano[2,3-c]pyrazole-5-carbonitrile* (**11s**). Yellow solid. Yield, 42%. ^1^H-NMR (400 MHz, DMSO-*d6*) δ 12.81 (s, 1H), 7.73 (d, *J* = 8.1 Hz, 2H), 7.59 (d, *J* = 7.8 Hz, 2H), 7.45–7.41 (m, 2H), 7.30–7.21 (m, 3H), 6.89 (s, 1H), 6.87 (s, 2H), 6.66 (dd, *J* = 8.9, 3.1 Hz, 1H), 6.53 (s, 1H), 5.32 (s, 1H), 5.19–5.09 (m, 2H), 3.58 (s, 3H). ^13^C-NMR (126 MHz, DMSO-*d6*) δ 161.06, 156.89, 153.77, 149.66, 142.88, 137.87, 134.06, 129.20, 128.97, 128.72, 128.54 (q, *J* = 31.9 Hz), 127.94, 126.39, 125.68 (q, *J* = 3.4 Hz), 124.74 (q, *J* = 261.7 Hz), 121.16, 115.99, 113.98, 112.51, 97.79, 69.55, 57.68, and 55.65. HRMS (ESI) calculated for C_28_H_21_F_3_N_4_O_3_ [M + H]^+^: 519.1639, found: 519.1627. Purity: 99.66% (by HPLC).

*6-Amino-4-(4-chloro-5-methoxy-2-((4-(trifluoromethyl)benzyl)oxy)phenyl)-3-methyl-1,4-dihydropyrano[2,3-c]pyrazole-5-carbonitrile* (**11t**)**.** White solid. Yield, 44%. ^1^H-NMR (500 MHz, DMSO-*d6*) δ 12.05 (s, 1H), 7.74 (d, *J* = 8.1 Hz, 2H), 7.55 (d, *J* = 6.5 Hz, 2H), 7.23 (s, 1H), 6.87 (s, 3H), 5.30–5.09 (m, 2H), 5.00 (s, 1H), 3.73 (s, 3H), 1.79 (s, 3H). ^13^C-NMR (126 MHz, DMSO-*d6*) δ161.81, 155.56, 149.91, 149.41, 142.20, 135.76, 132.29, 128.67 (q, *J* = 28.1 Hz), 128.24, 125.72 (q, *J* = 3.8 Hz), 124.73 (q, *J* = 272.6 Hz), 121.30, 120.18, 115.64, 114.21, 97.26, 69.79, 56.95, 56.25, and 10.08. HRMS (ESI) calculated for C_23_H_18_ClF_3_N_4_O_3_ [M + H]^+^: 491.1092, found: 491.1089. Purity: 98.50% (by HPLC).

*3-(4-(Trifluoromethyl)phenyl)propan-1-ol* (**13**). Lithium aluminum (6.9 mmol) was slowly added to a solution of **12** (4.6 mmol) in tetrahydrofuran at 0 °C. Then, the mixture was refluxed at 85 °C for 6 h. After the addition was complete, water/10% sodium hydroxide/water = 1:1:3 (mL:mL:mL) was added. Then, the solution was filtered and the filtrate was collected. After evaporation of the solvent under reduced pressure, the crude product was purified on a silica gel column to obtain compound **13**. White solid. Yield, 56%. ^1^H-NMR (400 MHz, CDCl_3_) δ 7.56 (d, *J* = 8.1 Hz, 2H), 7.34 (d, *J* = 8.3 Hz, 2H), 3.71 (dd, *J* = 10.3, 5.9 Hz, 2H), 2.83–2.75 (m, 2H), 1.97–1.87 (m, 2H).

*1-(3-Chloropropyl)-4-(trifluoromethyl)benzene* (**9o**). Triethylamine (2.5 mmol) was added to a solution of **13** (1.0 mmol) in anhydrous dichloromethane (10 mL) at 0 °C, followed by triphosgene (0.5 mmol) in one portion. The mixture was stirred at 0 °C for 5 min and then allowed to warm to room temperature. After the reaction was complete, the reaction mixture was poured into an aqueous solution containing saturated sodium bicarbonate. After separation of the layers, the aqueous layer was extracted again with dichloromethane (2 × 30 mL). Then, the organic layers were combined, dried with MgSO_4_, filtered, and concentrated in vacuo, and the crude product was purified on a silica gel column to obtain compound **9o**. White solid. Yield, 71%. ^1^H-NMR (400 MHz, CDCl_3_) δ 7.58 (d, *J* = 8.0 Hz, 2H), 7.34 (d, *J* = 7.9 Hz, 2H), 3.55 (t, *J* = 6.4 Hz, 2H), 2.88 (t, *J* = 7.5 Hz, 2H), 2.17–2.06 (m, 2H).

*Tert-butyl-6-amino-5-cyano-4-(5-methoxy-2-((4-(trifluoromethyl)benzyl)oxy)phenyl)-3-methylpyrano[2,3-c]pyrazole-1(4H)-carboxylate* (**14**). (Boc)_2_O (1.2 mmol) was added to a solution of 11h (1 mmol) and DMAP (0.2 mmol) in tetrahydrofuran (15 mL) at 0 °C, and then, the mixture was stirred at room temperature for 8 h. After completion, the mixture was extracted with water (30 mL) and the organic layer was separated. After evaporation of the organic layer under reduced pressure, the crude product was purified on a silica gel column to obtain compound 14. White solid. Yield, 60%. ^1^H-NMR (400 MHz, DMSO-*d6*) δ 7.68 (d, *J* = 7.9 Hz, 2H), 7.46 (d, *J* = 6.7 Hz, 2H), 7.07 (s, 2H), 6.97 (d, *J* = 8.8 Hz, 1H), 6.78 (d, *J* = 8.7 Hz, 2H), 5.19–5.09 (m, 2H), 4.89 (s, 1H), 3.68 (s, 3H), 2.08 (s, 3H), 1.52 (s, 9H). ^13^C-NMR (101 MHz, DMSO-*d6*) δ 161.03, 156.82, 153.78, 149.63, 148.24, 142.52, 140.27, 132.29, 128.73, 128.38(q, *J* = 33.3 Hz), 128.14, 125.59 (q, *J* = 3.7 Hz), 124.70 (q, *J* = 245.8 Hz), 120.72, 116.47, 114.32, 112.78, 105.48, 85.13, 69.23, 56.45, 55.74, 27.87, 27.79 and 13.19. LC/MS (ESI): 557.20 [M + H]^+^.

*Tert-butyl-5-cyano-6-(dimethylamino)-4-(5-methoxy-2-((4-(trifluoromethyl)benzyl) oxy)phenyl)-3-methylpyrano[2,3-c]pyrazole-1(4H)-carboxylate* (**15**)**.** CH_3_I (2 mmol) was added to a solution of K_2_CO_3_ (2.1 mmol) and 14 (1 mmol) in acetonitrile (20 mL), and the mixture was stirred at 60 °C for 5 h. After the reaction was complete, the solvent was spin-dried under reduced pressure. The mixture was diluted with water (20 mL) and extracted with ethyl acetate (60 mL), and then, the organic layer was separated. After evaporation of the solvent under reduced pressure, the crude product was purified on a silica gel column to obtain compound 15. White solid. Yield, 53%. ^1^H-NMR (400 MHz, DMSO-*d6*) δ 7.75 (d, *J* = 7.9 Hz, 2H), 7.63 (d, *J* = 8.0 Hz, 2H), 7.35 (d, *J* = 1.9 Hz, 1H), 7.08 (d, *J* = 8.8 Hz, 1H), 6.91 (dd, *J* = 9.1, 2.2 Hz, 1H), 5.19 (s, 2H), 4.93 (s, 1H), 3.84 (s, 3H), 3.73 (s, 3H), 2.20 (s, 3H), 1.87 (s, 3H), 1.52 (s, 9H). ^13^C-NMR (126 MHz, DMSO-*d6*) δ 162.03, 153.33, 149.52, 148.14, 144.10, 142.20, 128.85 (q, *J* = 31.9 Hz), 128.66, 125.95, 125.70 (q, *J* = 3.8 Hz), 124.69 (q, *J* = 291.9 Hz), 117.01, 116.99, 115.75, 114.14, 113.98, 106.59, 84.88, 69.82, 56.23, 55.75, 35.83, 27.90, 24.80 and 13.24. LC/MS (ESI): 585.19 [M + H] ^+^.

*6-(Dimethylamino)-4-(5-methoxy-2-((4-(trifluoromethyl)benzyl)oxy)phenyl)-3-methyl-1,4-dihydropyrano[2,3-c]pyrazole-5-carbonitrile* (**16**). Compound 15 (1 mmol) was added to the solution of trifluoroacetic acid/water = 1:3 (mL:mL), and the mixture was stirred at room temperature for 1 h. After the reaction was complete, the mixture was diluted with saturated sodium carbonate, and then, the organic layer was separated and the solvent was spin-dried under reduced pressure. The crude product was purified on a silica gel column to obtain compound 16. White solid. Yield, 77%. ^1^H-NMR (400 MHz, DMSO-*d6*) δ 11.74 (s, 1H), 7.75 (d, *J* = 8.1 Hz, 2H), 7.60 (d, *J* = 8.1 Hz, 2H), 7.49 (d, *J* = 3.0 Hz, 1H), 7.05 (d, *J* = 9.1 Hz, 1H), 6.88 (dd, *J* = 9.0, 3.0 Hz, 1H), 5.17 (s, 2H), 4.83 (s, 1H), 3.77 (s, 3H), 3.71 (s, 3H), 1.95 (s, 3H), 1.81 (s, 3H). ^13^C-NMR (101 MHz, DMSO-*d6*) δ 161.57, 153.34, 149.44, 142.44, 139.50, 128.72 (q, *J* = 34.4 Hz), 128.29, 127.35, 125.72 (q, *J* = 4.2 Hz), 124.72 (q, *J* = 252.4 Hz), 117.46, 117.27, 115.68, 114.04, 113.68, 97.81, 69.78, 55.91, 55.72, 36.51, 24.78 and 10.53. HRMS (ESI) calculated for C_25_H_23_F_3_N_4_O_3_[M + H]^+^: 485.1795, found: 485.1783. Purity: 96.81% (by HPLC).

### 3.2. Chiral Resolution of Compound ***11h***

The chiral resolution of compound **11h** was performed using Gilson instruments, and the chromatographic column was a CHIRALPAK^®^IF column (250 mm × 4.6 mm, 5 µm). The compound was filtered with a microporous membrane with an injection volume of 5 µL, and the mobile phase was Hex/EtOH = 80: 20. The eluent was ultrasonically degassed for 30 min before use. Flow rate: 1 mL/min; Cycle time: 40 min. (**−**)-**11h**: ${\left[\alpha \right]}_{D}^{20}$ = −46.7 (c 1.0, THF), t_R_ = 24.847 min, ee value, >99%; (**+**)-**11h**: ${\left[\alpha \right]}_{D}^{20}$ = +46.5 (c 1.0, THF), t_R_ = 33.976 min, ee value, >99%.

### 3.3. In Vitro Assay for PDE2 Inhibitors

#### 3.3.1. Protein Expression and Purification

Purification and expression of the recombinant pET15b-PDE2A plasmid (catalytic domain, 580–919) was performed following our previously reported method. The plasmid pET15b-PDE2A (catalytic domain, 580–919) was transferred to thawed competent *E. coli* BL21 cells (CodonPlus, Agilent Technologies, Santa Clara, CA, USA), which were grown in 2XYT medium with 100 μg/mL ampicillin and 20 μg/mL chloramphenicol at 37 °C until OD600 = 0.6–0.8. Then, 0.1 mM isopropylthiogalactoside was added for induction, and protein expression was induced by continuing to grow the cells at 16 °C for 24 h. After that, bacteria were collected by centrifugation and frozen overnight at −20 °C. The bacteria containing the recombinant plasmid of the target protein were subjected to cell disruption and passed through a Ni-NTA agarose affinity column, Q-Sepharose ion-exchange column and other steps. The purity can be 95% or more, and a typical purification batch produces 10–20 mg of PDE2A protein from 1.0 L of cell culture.

#### 3.3.2. Enzymatic Assay

Using the appropriate concentration of ^3^H-cGMP as the substrate, different compound solutions were added to the test group, DMSO was added to the negative control and blank control groups, and EHNA was added to the positive control group, followed by protein solution (the proteins were all diluted with Assay Buffer); Assay Buffer was added to the blank group. The enzymatic reaction was performed at room temperature (25 °C) for 15 min, and then, 0.2 M ZnSO_4_ was added to inactivate PDE2 and to stop the reaction. Then, 0.2 N Ba(OH)_2_ solution was added to precipitate the reaction product, and unreacted ^3^H-cGMP remained in the supernatant. After mixing the system, it was centrifuged. The radioactivity of the supernatant was measured with a liquid scintillation counter, and the inhibitory activity of the compound against PDE2 was calculated. The above test was repeated three times (the IC_50_ test generally used 9 concentrations, and the single-point test was directly diluted with DMSO to the required concentration).

### 3.4. Molecular Docking and Dynamic Simulation

#### 3.4.1. Molecular Docking

The Accelrys Discovery Studio 2.5.5 software (Accelrys, San Diego, CA, USA) was used for molecular docking research through the CDOCKER method, and the CHARMM force field and Momany–Rone partial charge method were used to add hydrogen atoms and charges to the crystal structure of PDE2A (PDB ID: 4HTX, resolution: 1.90 Å). All ionizable amino acid residues in the system were set to their protonated state at neutral pH, and the charge of zinc and magnesium ions were +2. (***R***)-**LZ77** was used to define the active site of PDE2A and to test the docking. The radius of the input ball was set to 10 Å from the center of the binding site, and the default values were used for the rest of the docking parameters. Fifty random images were generated and optimized for each ligand. The output pose of the docking was evaluated by docking scoring and visual inspection.

#### 3.4.2. Molecular Dynamics (MD) Simulations

MD simulations were conducted using Amber16 (University of California, San Francisco, CA, USA) for a more precise prediction of binding patterns between each ligand and PDE2. The partial atomic charges of each ligand were calculated using the Hartree–Fock method at the 6–31G* level in Gaussian 03 (Gaussian, Inc., Wallingford, CT, USA). Then, Antechamber (Amber16) was applied to fit the restricted electrostatic potential (RESP) and to assign the general amber force field (GAFF) parameters. For the PDE2 protein, the Amber14SB force field was assigned. For the force field parameters of Zn^2+^ and Mg^2+^, the “nonbond model” method was applied for the assignment. The protonation states of amino acid residues were set according to pH = 7.0. Each simulation system was solvated with an 8 Å TIP3P water box in the form of a truncated octahedron. Na^+^ ions were added for neutralization of the whole system.

Each system was investigated through MD simulations. After energy minimization, the system was gradually heated in the NPT ensemble from 10 to 300 K over 25 ps. Then, eight-nanosecond MD simulations were conducted in the NPT ensemble with a constant pressure and temperature of 1 atm and 300 K, respectively. Periodic boundary conditions were utilized. An 8 Å cutoff for long-range electrostatic interactions with the partial mesh Ewald (PME) method was simultaneously employed. The SHAKE algorithm was adopted to restrict bonds involving hydrogen atoms, and thus, the time step was set to 2 fs. An Intel(R) Xeon(R) Bronze 3106 CPU and a GeForce RTX 2080Ti GPU were employed to accelerate the process of MD simulations for each system.

#### 3.4.3. Binding Free Energy Calculations

MM-PBSA binding free energy calculations were performed by extracting 100 snapshots of trajectories in the last 1 ns with default parameters assigned. According to the MM-PBSA method, the binding free energies (Δ*G*_bind_) were calculated using Equation (1), in which *G*_complex_, *G*_rec_ and *G*_lig_ represent the free energies of the complex, receptor, and ligand, respectively. Each free energy value is calculated as the sum of the MM energy *E*_MM_, the solvation free energy *G*_solv_, and the entropy contribution *S*, respectively, leading to Equation (2). Δ*E*_MM_ represents the gas-phase interaction energy. It is decomposed into *E*_MM, complex_, *E*_MM, rec_, and *E*_MM, lig_. The solvation free energy represents the sum of the electrostatic solvation free energy and nonpolar solvation free energy. The entropy contribution for each system was omitted, since this computational process is extremely time-consuming for large protein–ligand systems.

Δ*G*_bind_ = *G*_complex_ − *G*_rec_ − *G*_lig_ (1)

Δ*G*_bind_ = Δ*E*_MM_ + Δ*G*_solv_ − *T*Δ*S* (2)

## 4. Conclusions

In summary, a series of dihydropyranopyrazole derivatives were designed, synthesized, and evaluated as PDE2 inhibitors with the assistance of molecular docking. The optimal compound is (***+***)-**11h**, in which the side chain on the benzene ring connected to the pyran ring is shifted from the 3-position to the 2-position, the chlorine on the side chain is replaced by a trifluoromethoxy group, and the methoxy group is shifted from the 4-position to the 5-position. (***+***)-**11h** exhibits potent inhibitory activity against PDE2, with an inhibition rate of 93.6% at 0.5 μM and an IC_50_ value of 41.5 nM. In addition, (***+***)-**11h** has high selectivity against PDE2 compared with several other PDEs. Furthermore, both the molecular docking of PDE2-(***+***)-**11h** and energy decomposition reveal that the side chain at position 2 of the compound enters the H-pocket and forms strong hydrophobic interactions with L770/L809/F862, which may contribute to its improved inhibitory activity. The above results may provide insight for further structural optimization of highly potent PDE2 inhibitors and lay the foundation for the use of PDE2 inhibitors in the treatment of AD.

## Figures and Tables

**Table 1:**
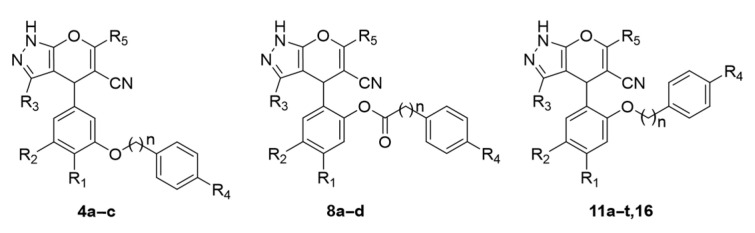
Inhibitory activities of synthesized compounds against PDE2.

Comp.	n	R_1_	R_2_	R_3_	R_4_	R_5_	Inhibition Ratio (%)		IC_50_ (nM) ^a^
							0.5 μM	0.1 μM	
***(R*)-LZ77**	1	OCH_3_	H	CH_3_	Cl	NH_2_	67.5	30.4	261.3 ± 11.2
**4a (LZ77)**	1	OCH_3_	H	CH_3_	Cl	NH_2_	57.2	18.2	407.2 ± 37.7
**4b**	1	H	OCH_3_	CH_3_	CF_3_	NH_2_	14.1	-	-
**4c**	1	H	OCH_3_	CH_3_	Cl	NH_2_	43.4	-	-
**8a**	0	H	OCH_3_	CH_3_	OCF_3_	NH_2_	13.4	-	-
**8b**	0	H	OCH_3_	CH_3_	CF_3_	NH_2_	39.1	-	-
**8c**	2	H	OCH_3_	CH_3_	Cl	NH_2_	4.12	-	-
**8d**	3	H	OCH_3_	CH_3_	Cl	NH_2_	6.12	-	-
**11a**	1	OCH_3_	H	CH_3_	OCH_3_	NH_2_	27.0	-	-
**11b**	1	OCH_3_	H	CH_3_	H	NH_2_	14.3	-	-
**11c**	1	OCH_3_	H	CH_3_	Cl	NH_2_	29.0	-	-
**11d**	1	H	OCH_3_	CH_3_	H	NH_2_	17.7	-	-
**11e**	1	H	OCH_3_	CH_3_	OCH_3_	NH_2_	48.0	17.5	458.4 ± 16.2
**11f**	1	H	OCH_3_	CH_3_	Cl	NH_2_	79.2	48.1	115.4 ± 1.3
**11g**	1	H	OCH_3_	CH_3_	F	NH_2_	44.4	15.1	807.5 ± 15.3
**11h**	1	H	OCH_3_	CH_3_	CF_3_	NH_2_	87.3	63.3	75.5 ± 2.0
**(*−*)-11h**	1	H	OCH_3_	CH_3_	CF_3_	NH_2_	87.3	-	-
**(*+*)-11h**	1	H	OCH_3_	CH_3_	CF_3_	NH_2_	93.6	75.9	41.5 ± 3.4
**11i**	1	H	OCH_3_	CH_3_	OCF_3_	NH_2_	48.8	-	-
**11j**	1	H	C(CH_3_)_3_	CH_3_	CF_3_	NH_2_	0	-	-
**11k**	1	H	CH_3_	CH_3_	CF_3_	NH_2_	72.0	38.3	153.5 ± 3.3
**11l**	1	H	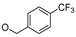	CH_3_	CF_3_	NH_2_	13.4	-	-
**11m**	1	H	OH	CH_3_	CF_3_	NH_2_	0	-	-
**11n**	2	H	OCH_3_	CH_3_	CF_3_	NH_2_	20.7	-	-
**11o**	3	H	OCH_3_	CH_3_	CF_3_	NH_2_	8.2	-	-
**11p**	1	H	OCF_3_	CH_3_	CF_3_	NH_2_	17.4	-	-
**11q**	1	H	OCHF_2_	CH_3_	CF_3_	NH_2_	6.6	-	-
**11r**	1	H	OCH_3_	H	CF_3_	NH_2_	60.5	22.7	445.3 ± 3.4
**11s**	1	H	OCH_3_	C_6_H_5_	CF_3_	NH_2_	75.3	46.3	153.7 ± 6.2
**11t**	1	Cl	OCH_3_	CH_3_	CF_3_	NH_2_	10.3	-	-
**16**	1	H	OCH_3_	CH_3_	CF_3_	N(CH_3_)_2_	9.6	-	-

^a^ Values are presented as the means ± SD of three experiments.

**Table 2:** Selectivity index of (***+***)-**11h** against PDE2 compared with several other PDE Families.

PDEs (Catalytic Domain)	IC_50_ (nM)	Selectivity Index
PDE2A (580–919)	30.31 ± 3.4	-
PDE1C (2–634)	8764 ± 667.5	289
PDE4D2 (86–413)	>10,000	>1149
PDE8A1(480–820)	>10,000	>1149

## Data Availability

The data presented in this study are available within the article and Supplementary Materials.

## Supplementary Materials

The spectra of ^1^H-NMR and ^13^C-NMR for the target compounds are available online.

## Sample Availability

Sample of the compounds are not available from authors.
