# Supplementary material for: Design, Synthesis, and Evaluation of Dihydropyranopyrazole Derivatives as Novel PDE2 Inhibitors for the Treatment of Alzheimer’s Disease
Source: Molecules. 2021 May 19;26(10):3034. doi: 10.3390/molecules26103034 (PMC8160813; doi:10.3390/molecules26103034)
Supplement: Supplementary file 1 [file molecules-26-03034-s001.zip › molecules-1214847-supplementary.pdf]

Design, Synthesis and Activity Evaluation of Dihydropyranopyrazole PDE2 inhibitors

Yan Zhou[1], Jinjian Li[1], Han Yuan[1], Rui Su[1], Yue Huang[1], Yiyou Huang[1], Zhe Li[1], Yinuo Wu[1], Hai-Bin Luo[1,2], Chen Zhang[1*], Ling Huang[1,2*]

[1] *School of Pharmaceutical Sciences, Sun Yat-Sen University, Guangzhou 510006, PR China*

[2] *School of Pharmaceutical Sciences, Hainan University, Haikou, 510006, PR China*

Corresponding author. E-mail address: zhangch328@mail.sysu.edu.cn (C. Zhang), huangl72@mail.sysu.edu.cn (L. Huang).

Table of Contents:

1. ^{1}HNMR and ^{13}CNMR spectrums of tested compounds..S2

2. HRMS spectra of target compounds..S32

3. Chiral Resolution of Compound **11h**...S60

1. ^{1}HNMR and ^{13}CNMR spectrums of the targeted compounds.

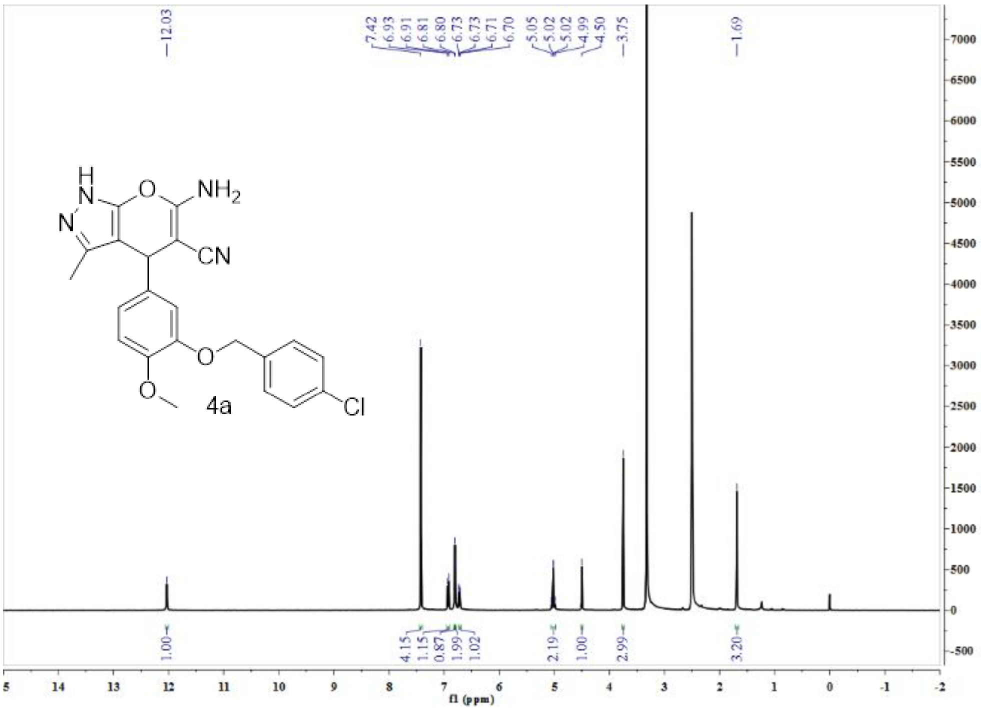

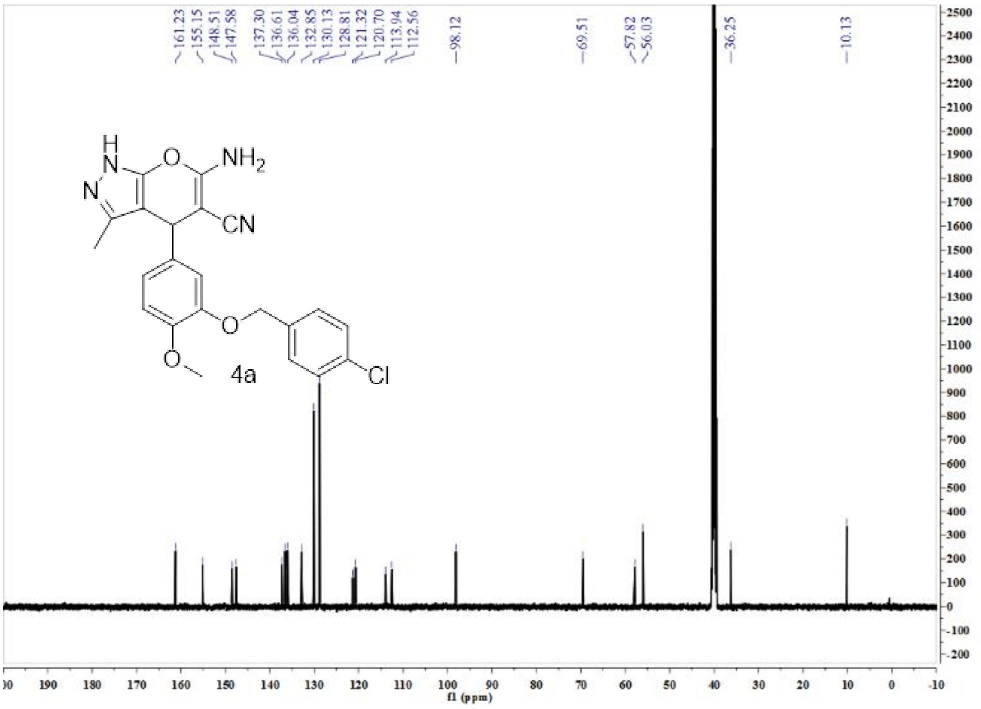

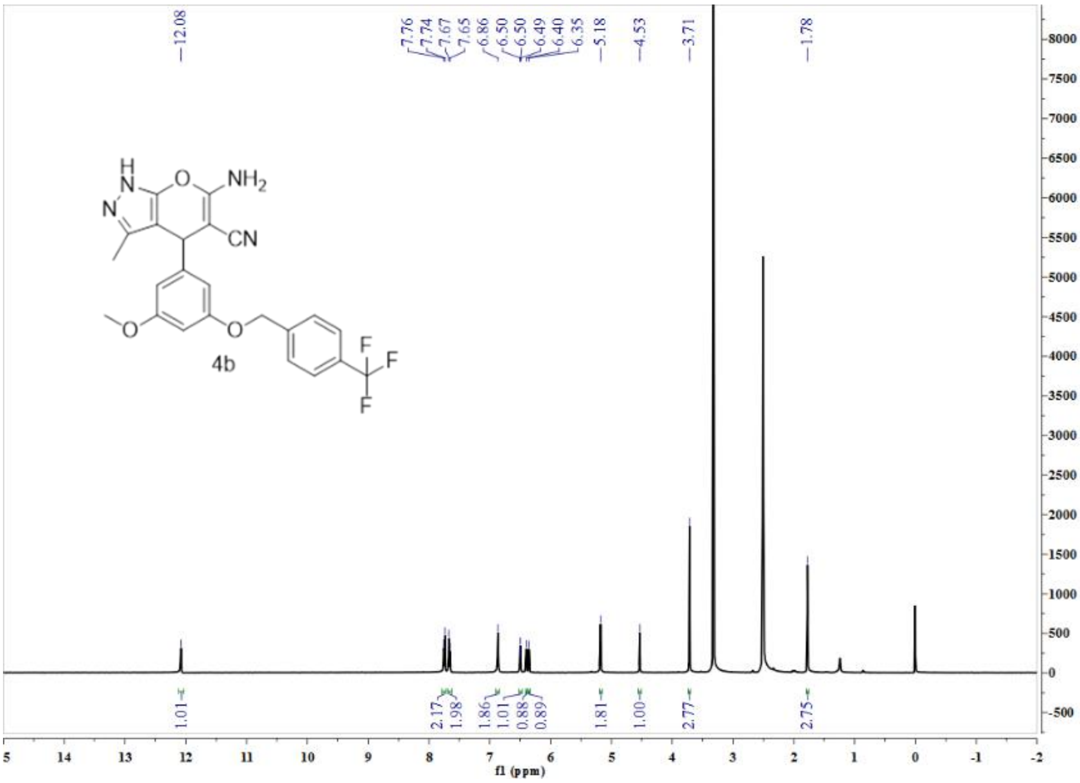
12.08
7.76
7.74
7.67
7.65
6.86
6.50
6.50
6.49
6.40
6.35
5.18
4.53
3.71
1.78
1.01
2.17
1.98
1.86
1.01
0.88
0.89
1.81
1.00
2.77
2.75
H
N
N
O
NH2
CN
O
O
4b
F
F
F
f1 (ppm)

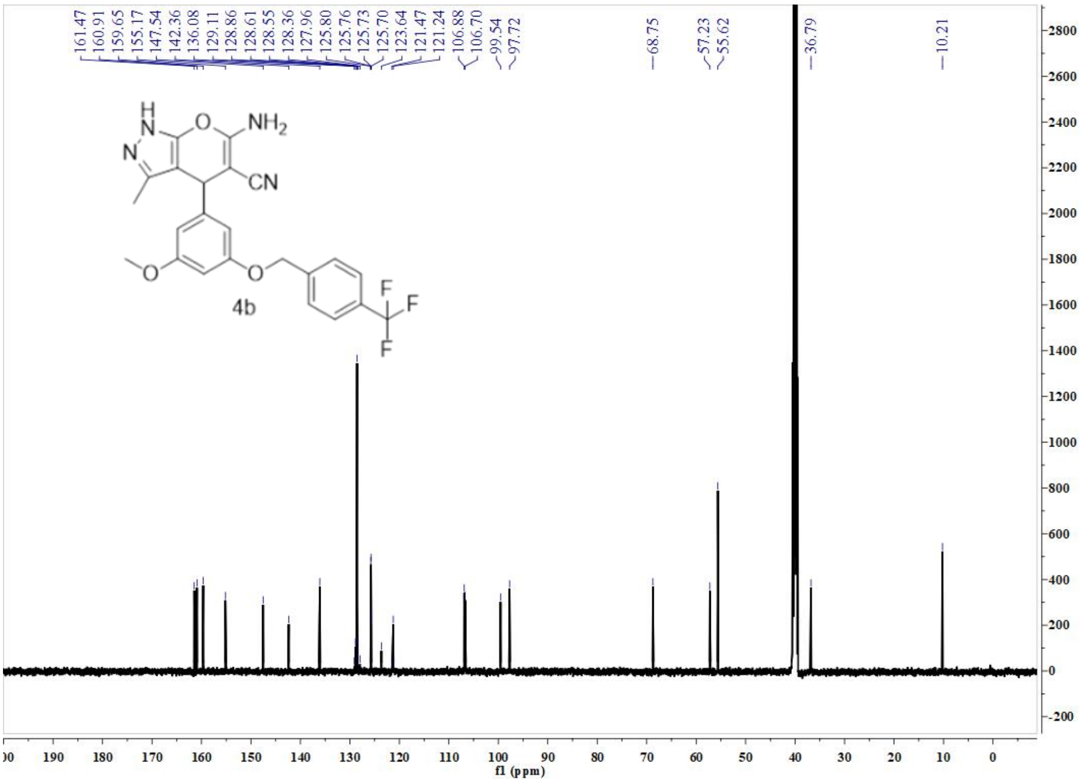
161.47
160.91
159.65
155.17
147.54
142.36
136.08
129.11
128.86
128.61
128.55
128.36
127.96
125.80
125.76
125.73
125.70
123.64
121.47
121.24
106.88
106.70
99.54
97.72
68.75
57.23
55.62
36.79
10.21
H
N
N
O
NH2
CN
O
O
4b
F
F
F
f1 (ppm)

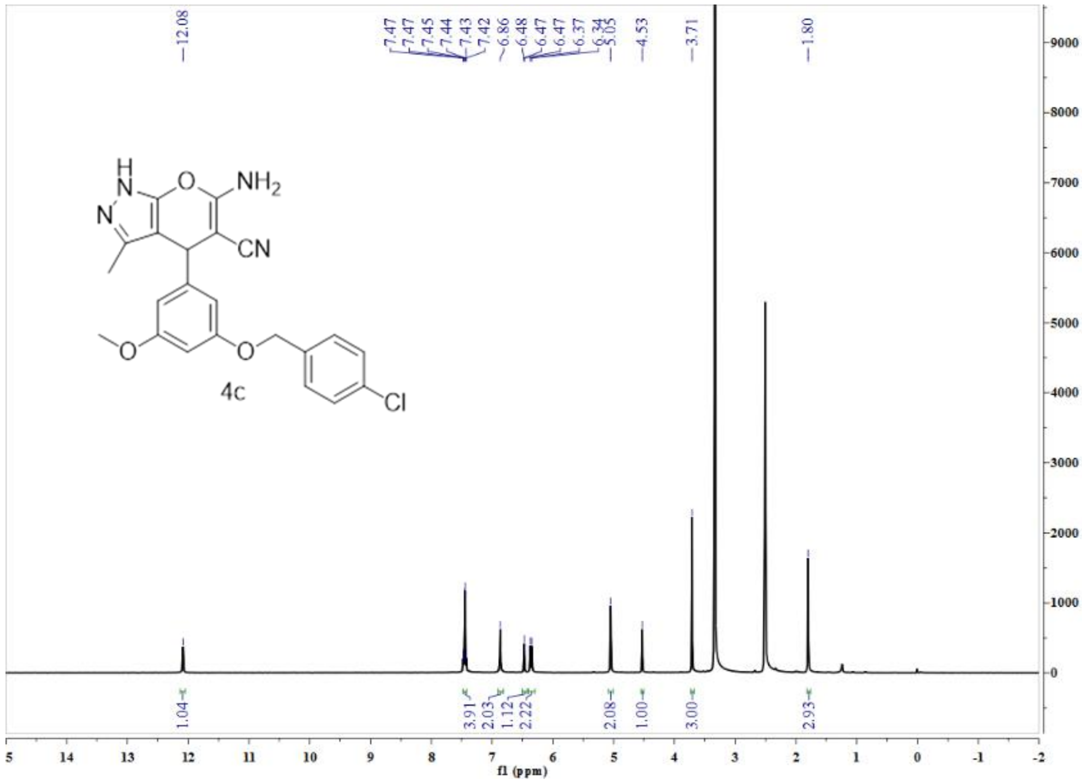

12.08
7.47
7.47
7.45
7.44
7.43
7.42
6.86
6.48
6.47
6.47
6.37
6.34
5.05
4.53
3.71
1.80
NH2
CN
N
H
O
Cl
4c
1.04
3.91
2.03
1.12
2.22
2.08
1.00
3.00
2.93
f1 (ppm)

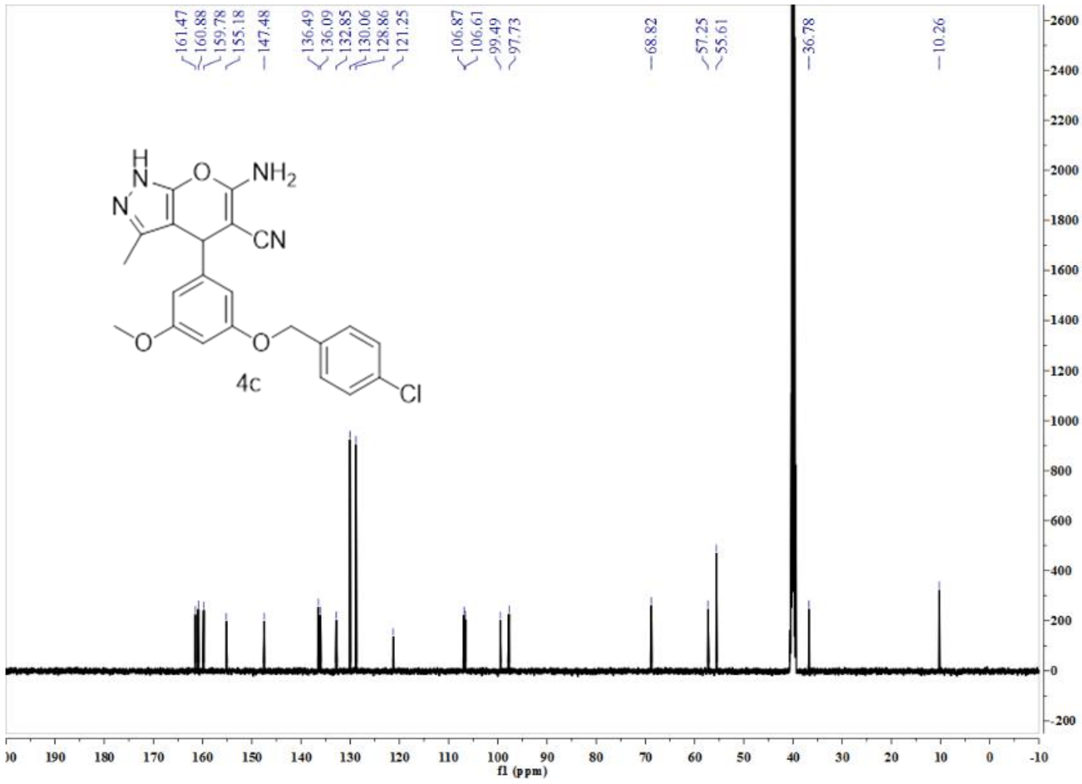

161.47
160.88
159.78
155.18
147.48
136.49
136.09
132.85
130.06
128.86
121.25
106.87
106.61
99.49
97.73
68.82
57.25
55.61
36.78
10.26
NH2
CN
N
H
O
Cl
4c
f1 (ppm)

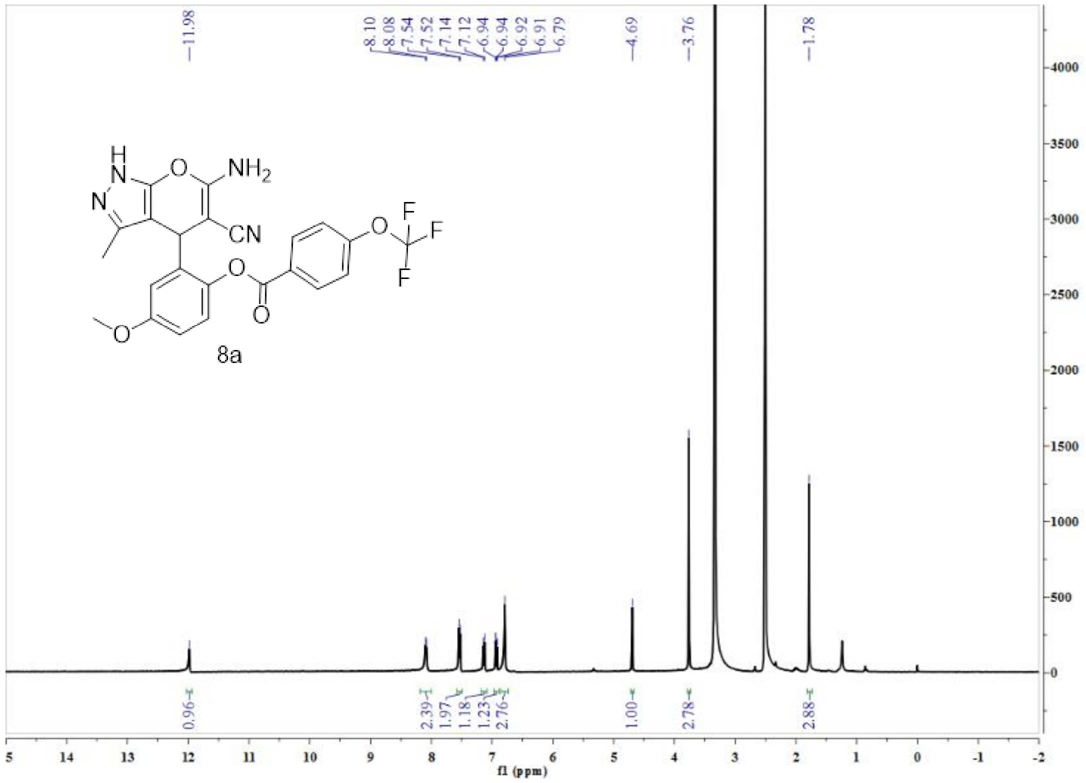
8a
f1 (ppm)

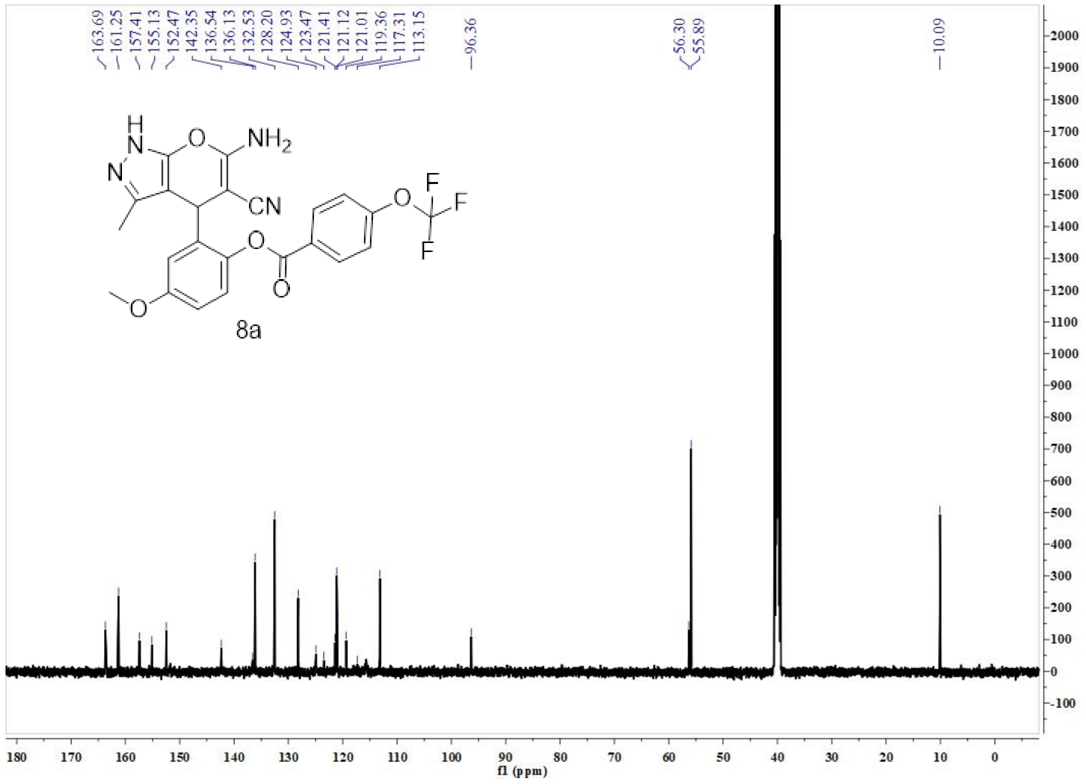
8a
f1 (ppm)

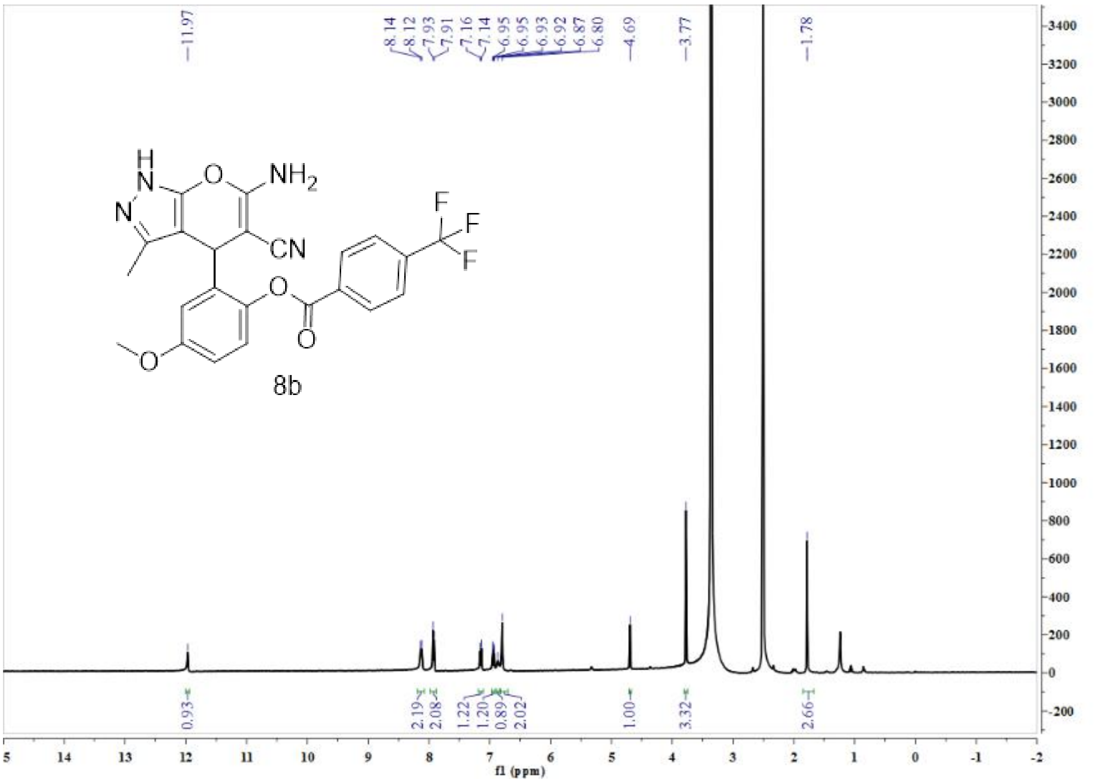
11.97
8.14
8.12
7.93
7.91
7.16
7.14
6.95
6.95
6.93
6.92
6.87
6.80
4.69
3.77
1.78
NH2
CN
F
F
F
N
N
H
O
O
O
O
8b
0.93
2.19
2.08
1.22
1.20
0.89
2.02
1.00
3.32
2.66
f1 (ppm)

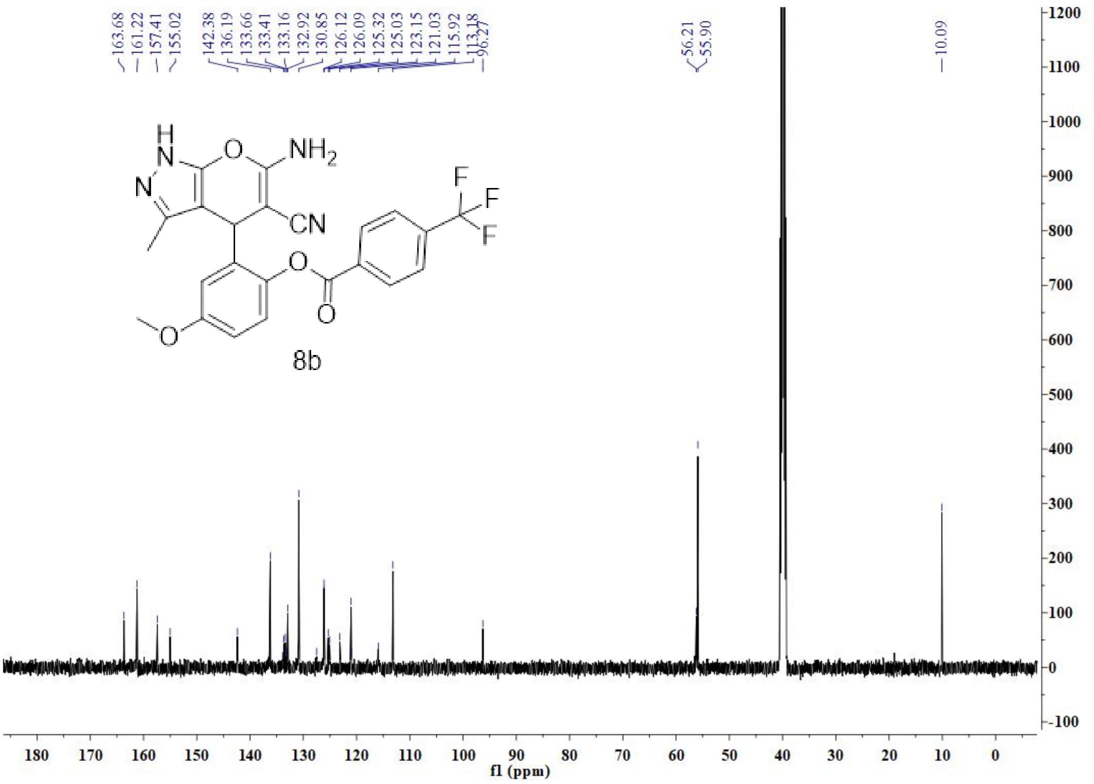
163.68
161.22
157.41
155.02
142.38
136.19
133.66
133.41
133.16
132.92
130.85
126.12
126.09
125.32
125.03
123.15
121.03
115.92
113.18
96.27
56.21
55.90
10.09
8b
f1 (ppm)

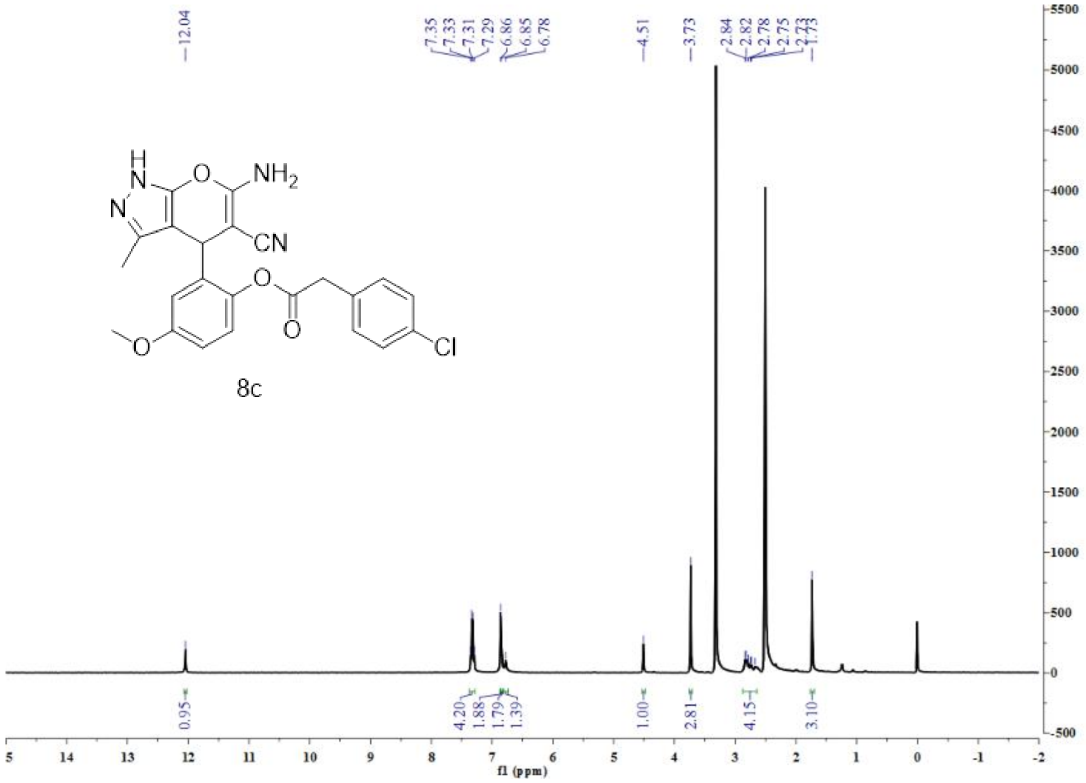
12.04
7.35
7.33
7.31
7.29
6.86
6.85
6.78
4.51
3.73
2.84
2.82
2.78
2.75
2.73
1.73
H
N
N
O
NH2
CN
O
O
O
Cl
8c
0.95
4.20
1.88
1.79
1.39
1.00
2.81
4.15
3.10
f1 (ppm)

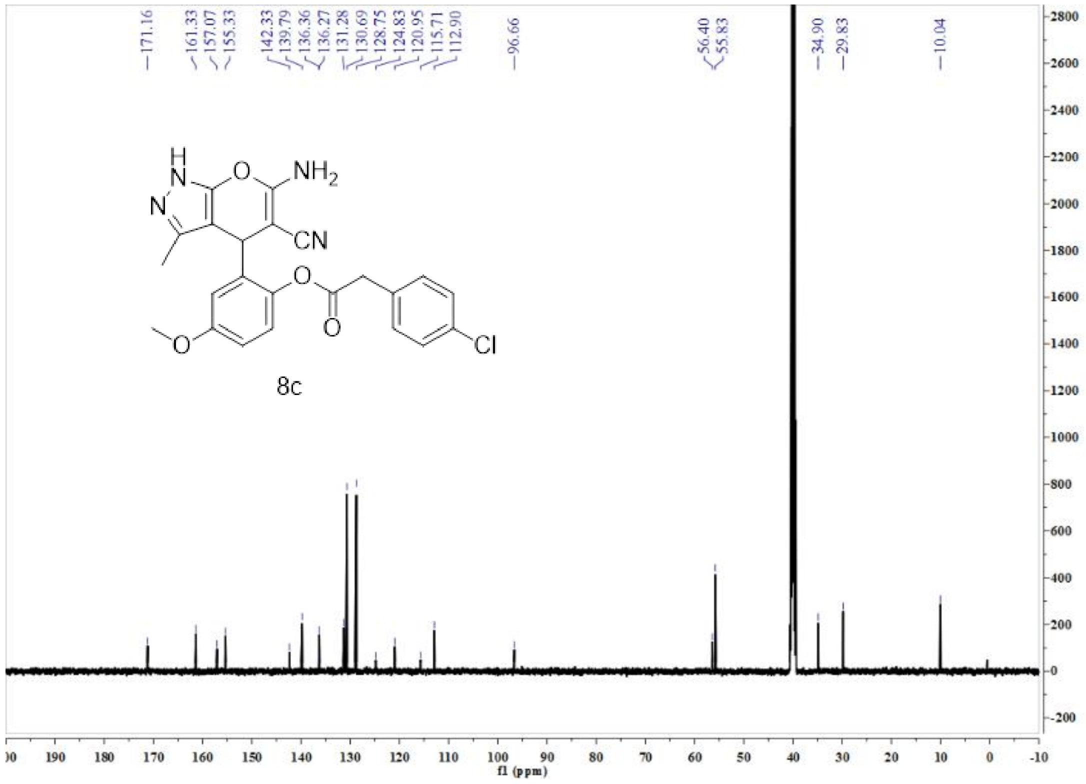
171.16
161.33
157.07
155.33
142.33
139.79
136.36
136.27
131.28
130.69
128.75
124.83
120.95
115.71
112.90
96.66
56.40
55.83
34.90
29.83
10.04
H
N
N
O
NH2
CN
O
O
O
Cl
8c
f1 (ppm)

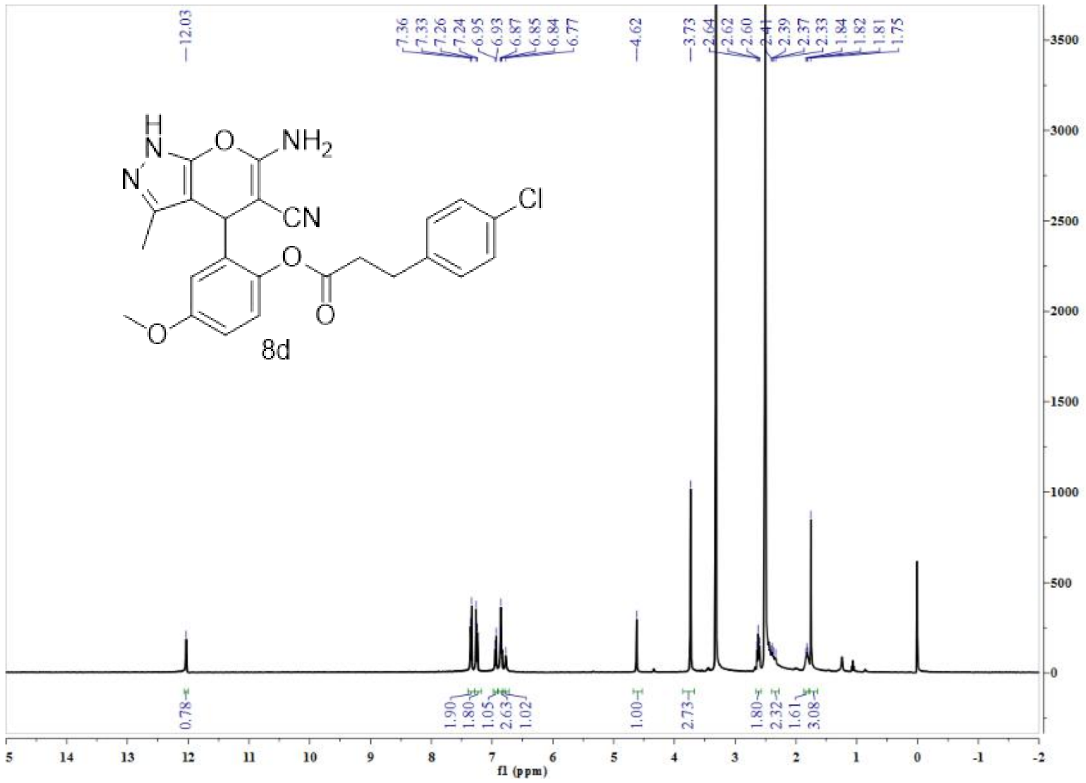

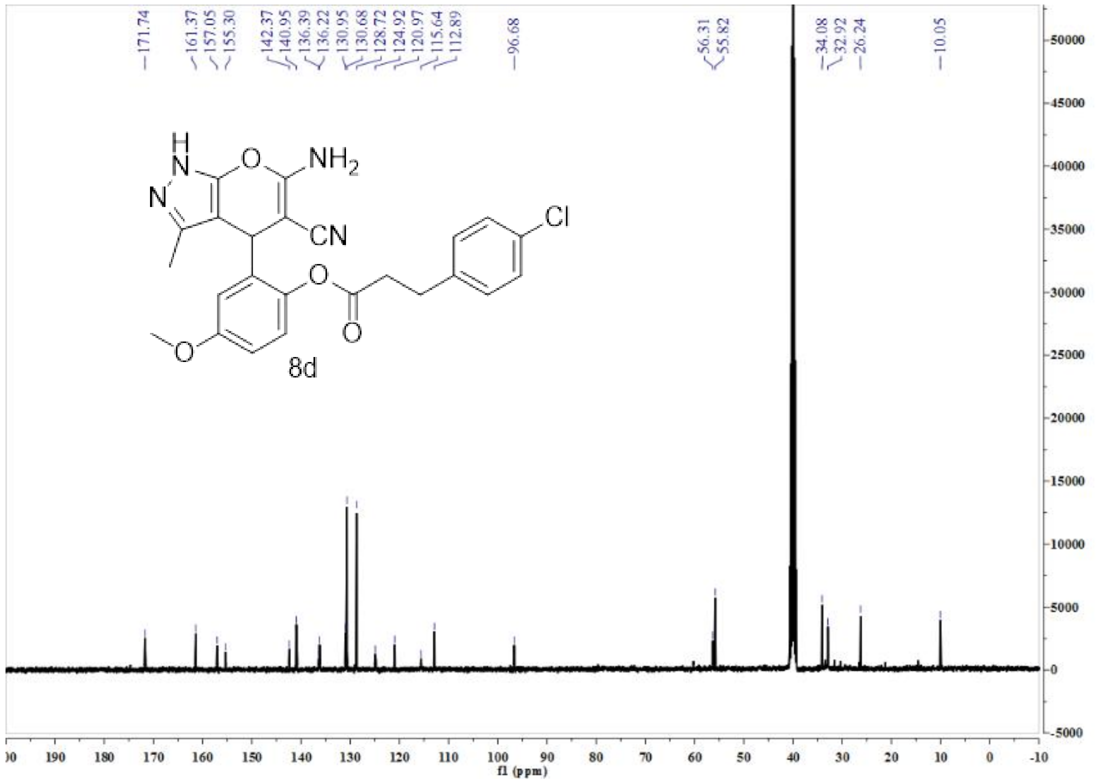

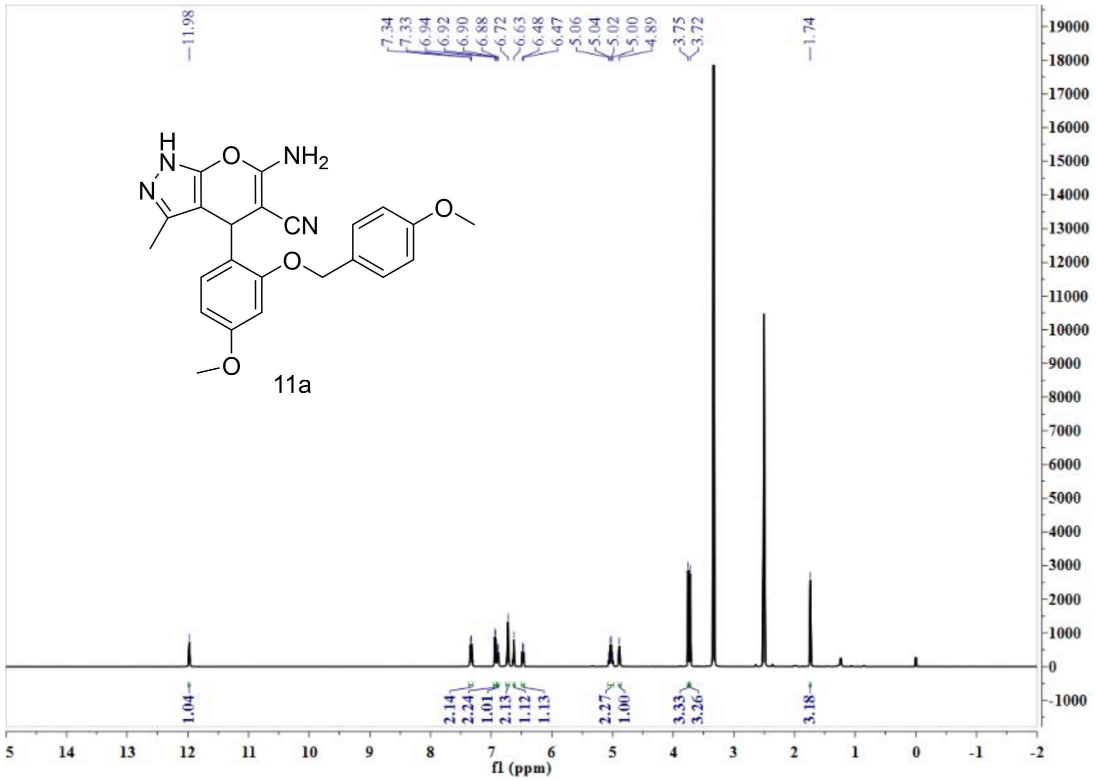

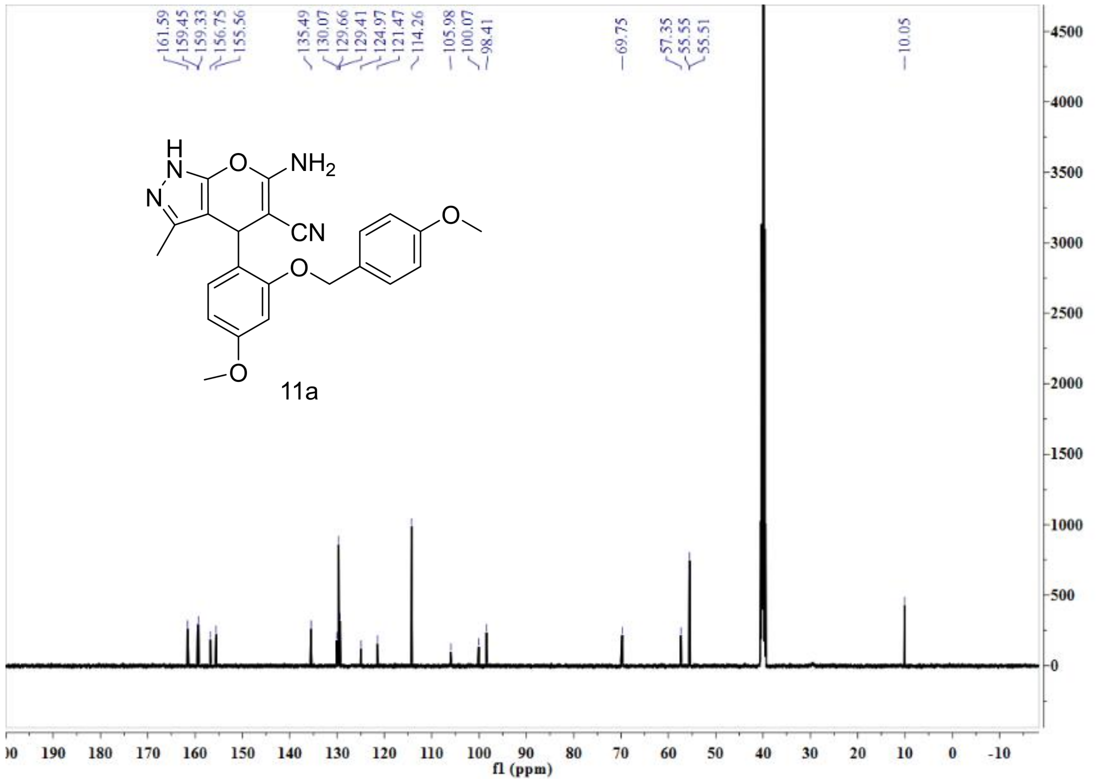

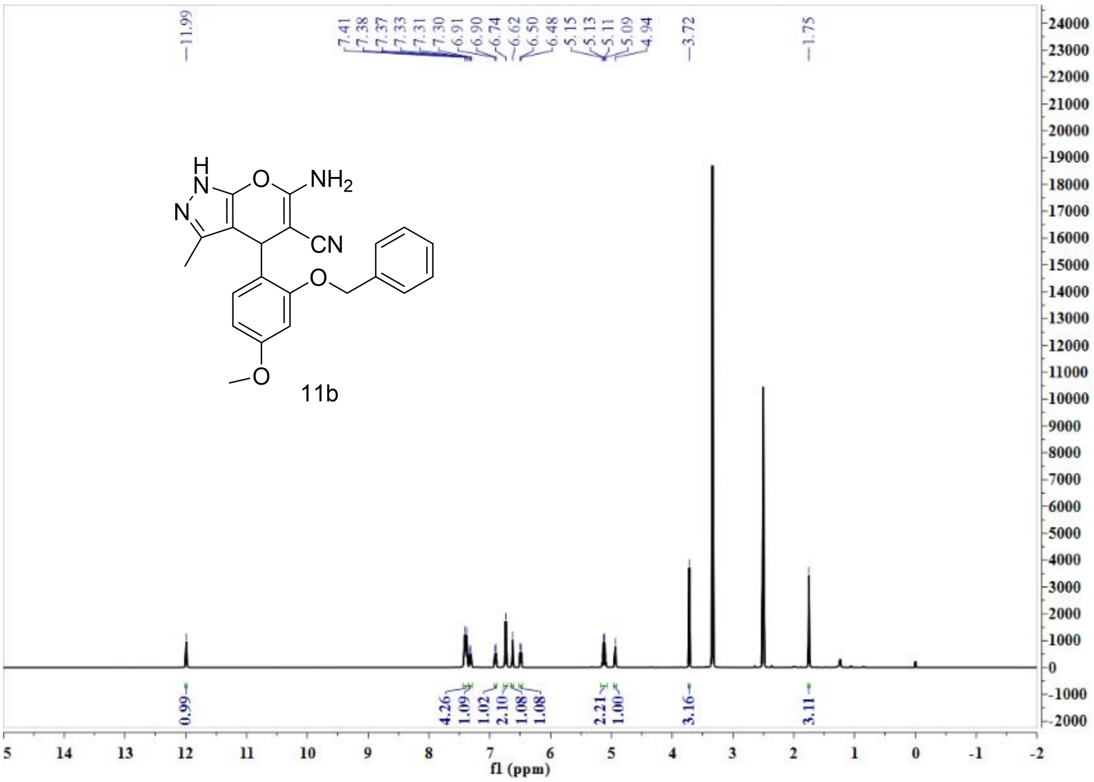
11.99
7.41 7.38 7.37 7.33 7.31 7.30 6.91 6.90 6.74 6.62 6.50 6.48 5.15 5.13 5.11 5.09 4.94
3.72
1.75
NH2
CN
11b
0.99
4.26 1.09 1.02 2.10 1.08 1.08
2.21 1.00
3.16
3.11
f1 (ppm)

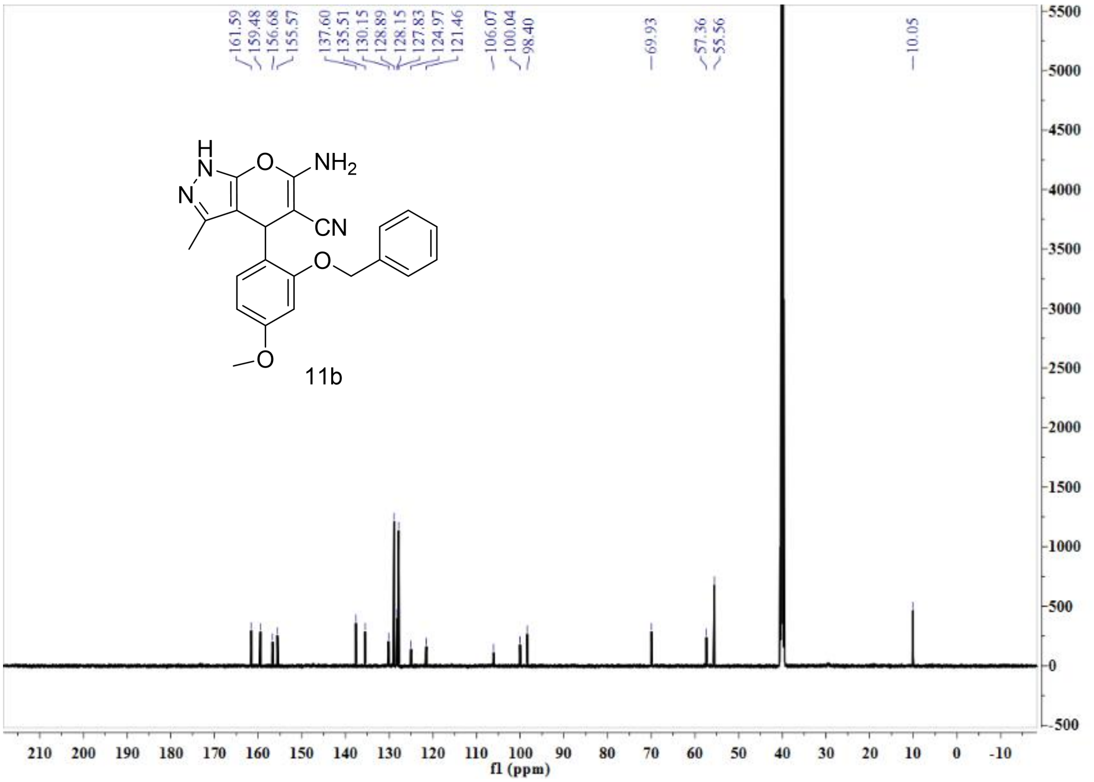
161.59 159.48 156.68 155.57
137.60 135.51 130.15 128.89 128.15 127.83 124.97 121.46
106.07 100.04 98.40
69.93
57.36 55.56
10.05
NH2
CN
11b
f1 (ppm)

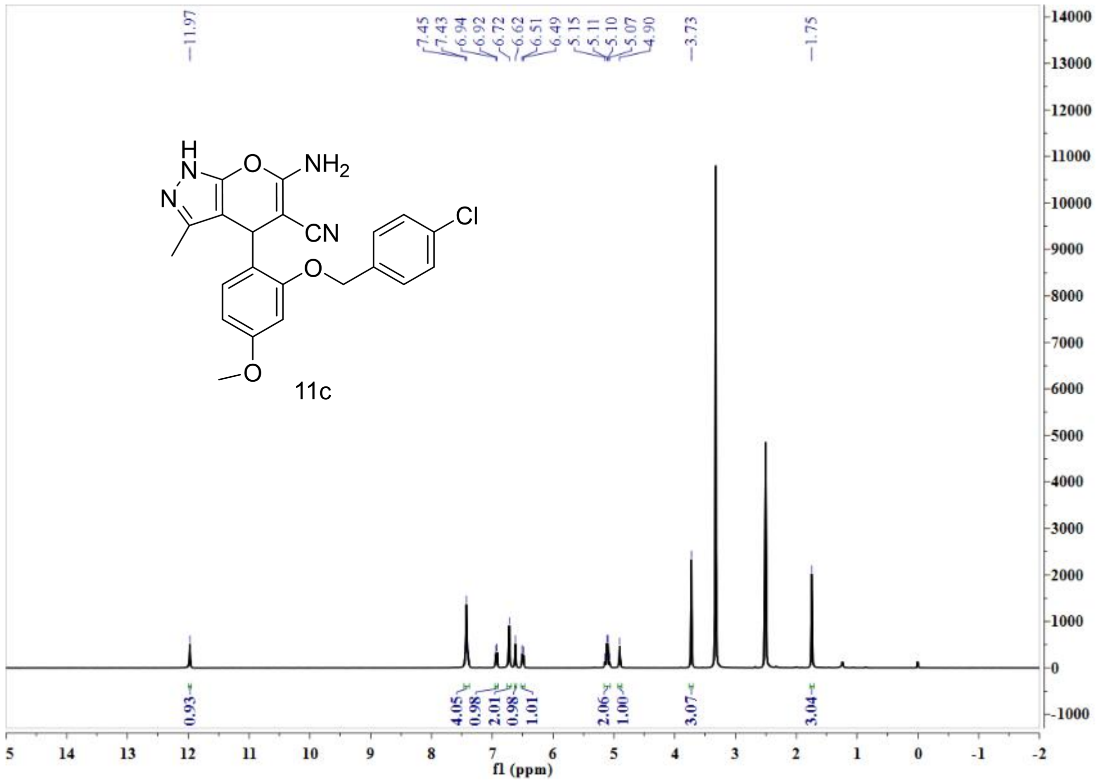
11.97
7.45
7.43
6.94
6.92
6.72
6.62
6.51
6.49
5.15
5.11
5.10
5.07
4.90
3.73
1.75
NH2
CN
Cl
O
N
H
11c
0.93
4.05
0.98
2.01
0.98
1.01
2.06
1.00
3.07
3.04
f1 (ppm)

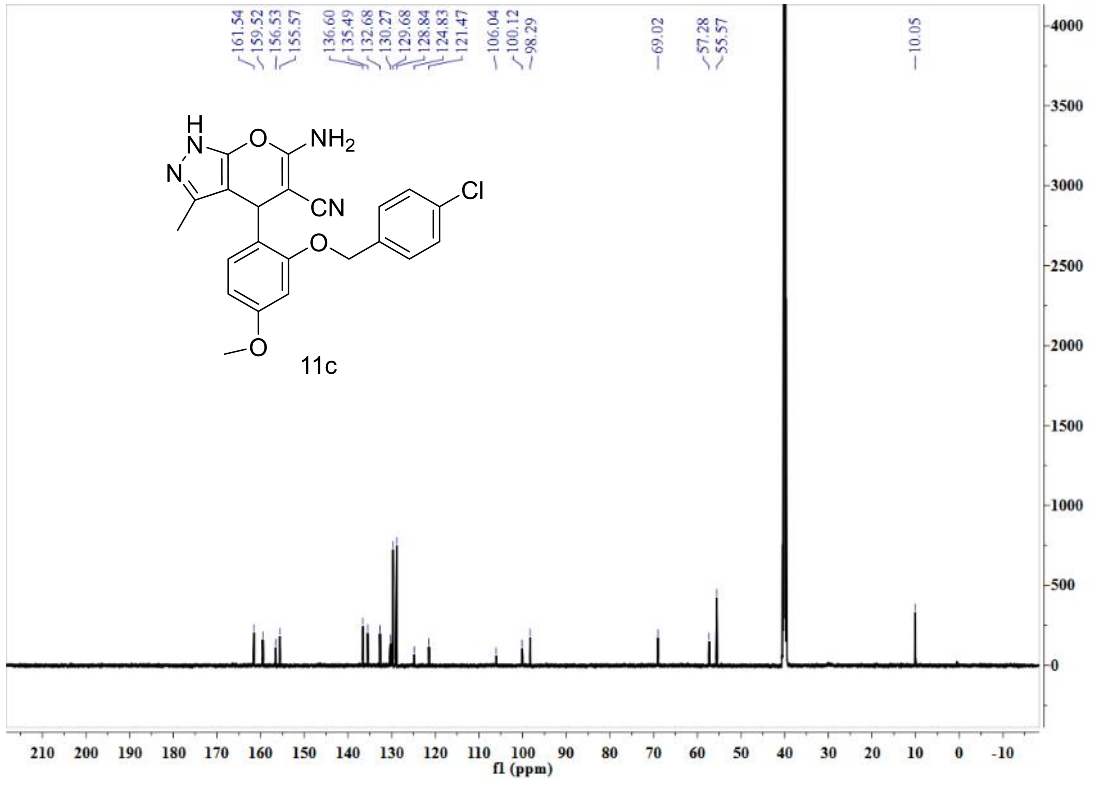
161.54
159.52
156.53
155.57
136.60
135.49
132.68
130.27
129.68
128.84
124.83
121.47
106.04
100.12
98.29
69.02
57.28
55.57
10.05
NH2
CN
Cl
11c
f1 (ppm)

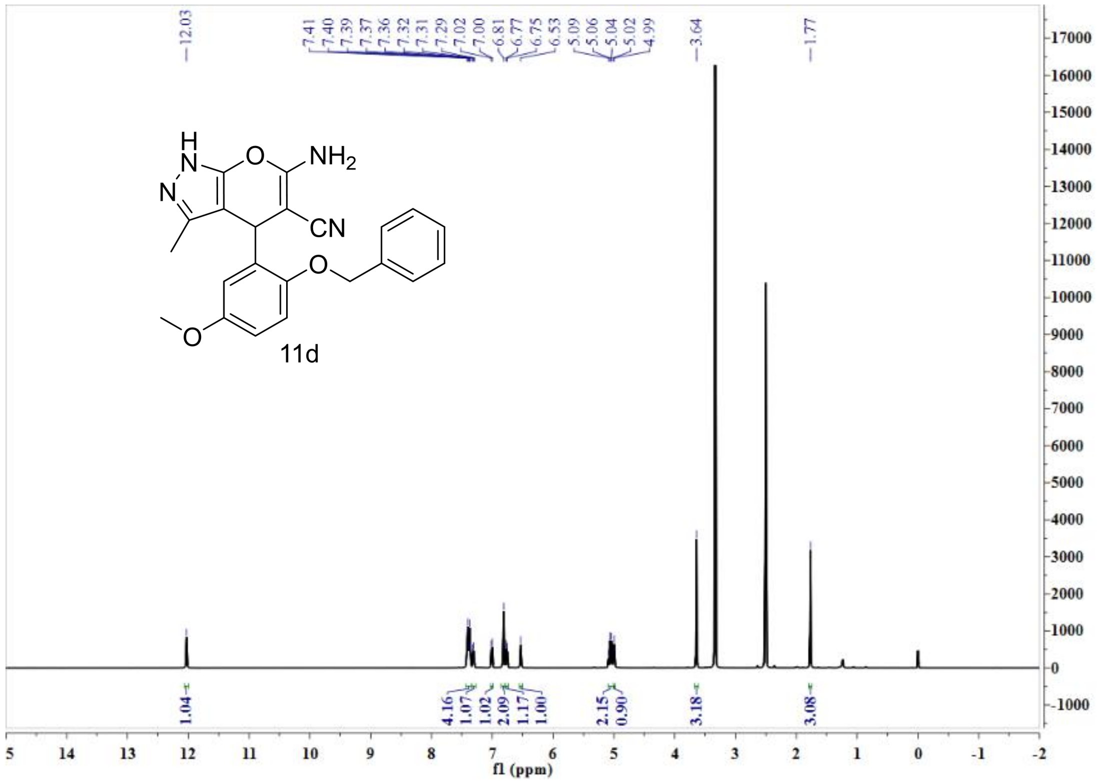
12.03
7.41
7.40
7.39
7.37
7.36
7.32
7.31
7.29
7.02
7.00
6.81
6.77
6.75
6.53
5.09
5.06
5.04
5.02
4.99
3.64
1.77
NH2
CN
N
O
H
11d
1.04
4.16
1.07
1.02
2.09
1.17
1.00
2.15
0.90
3.18
3.08
f1 (ppm)

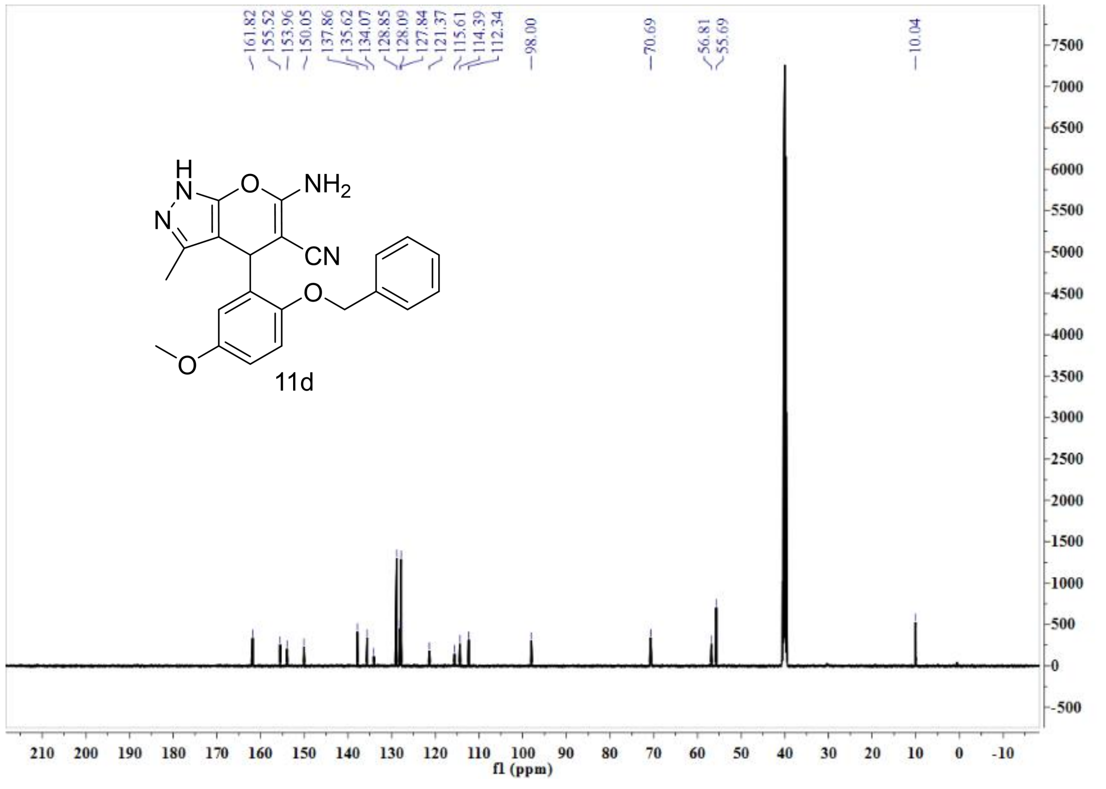
161.82
155.52
153.96
150.05
137.86
135.62
134.07
128.85
128.09
127.84
121.37
115.61
114.39
112.34
98.00
70.69
56.81
55.69
10.04
NH2
CN
11d
f1 (ppm)

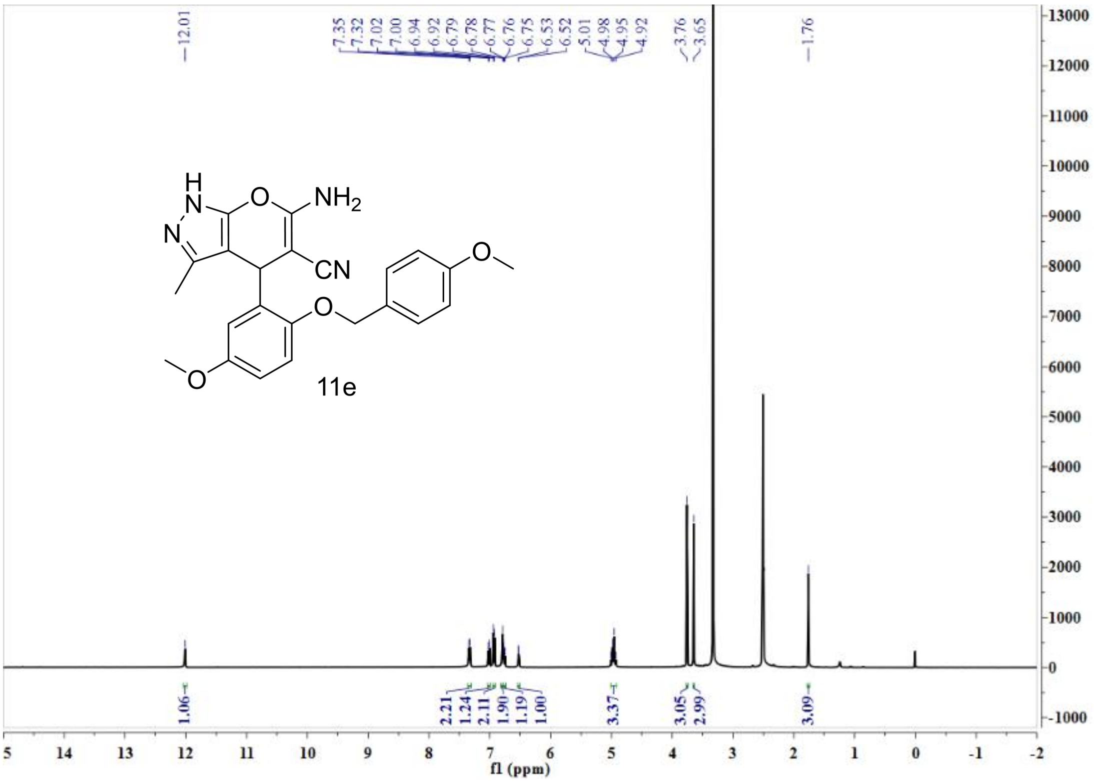
12.01
7.35 7.32 7.02 7.00 6.94 6.92 6.79 6.78 6.77 6.76 6.75 6.53 6.52 5.01 4.98 4.95 4.92
3.76 3.65
1.76
NH2
CN
11e
1.06 2.21 1.24 2.11 1.90 1.19 1.00 3.37 3.05 2.99 3.09
f1 (ppm)

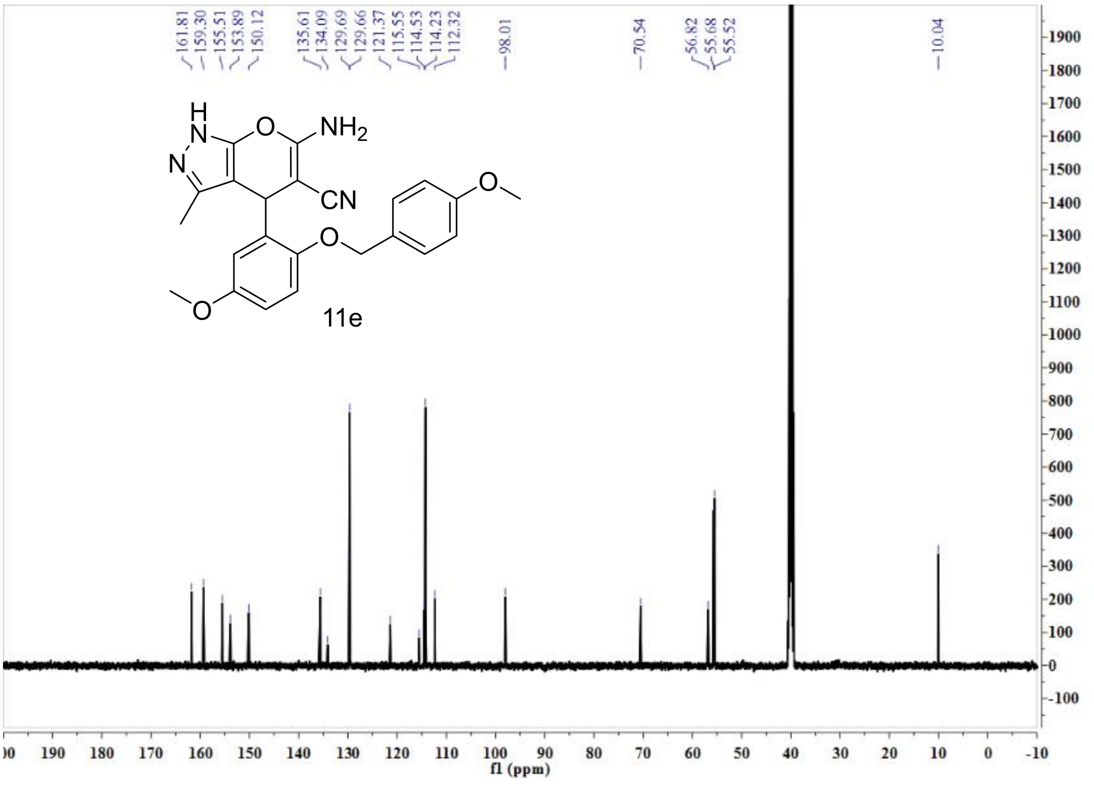
161.81 159.30 155.51 153.89 150.12
135.61 134.09 129.69 129.66 121.37 115.55 114.53 114.23 112.32
98.01
70.54
56.82 55.68 55.52
10.04
NH2
CN
11e
f1 (ppm)

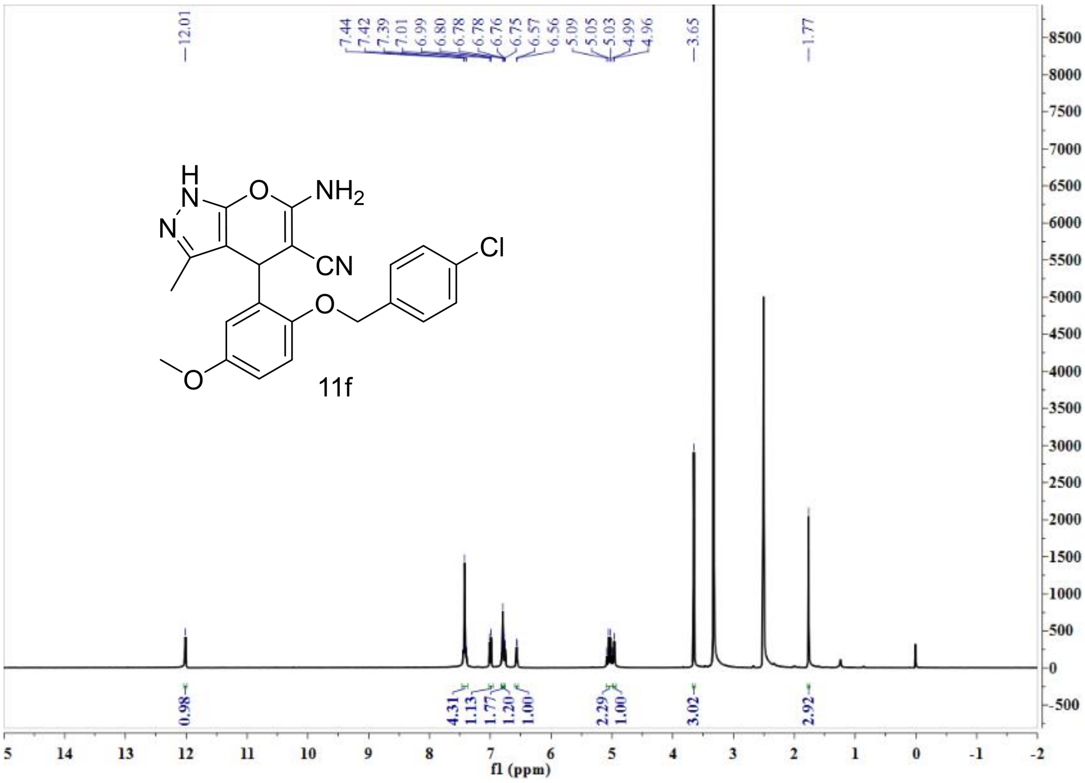
12.01
7.44
7.42
7.39
7.01
6.99
6.80
6.78
6.78
6.76
6.75
6.57
6.56
5.09
5.05
5.03
4.99
4.96
3.65
1.77
0.98
4.31
1.13
1.77
1.20
1.00
2.29
1.00
3.02
2.92
11f
f1 (ppm)

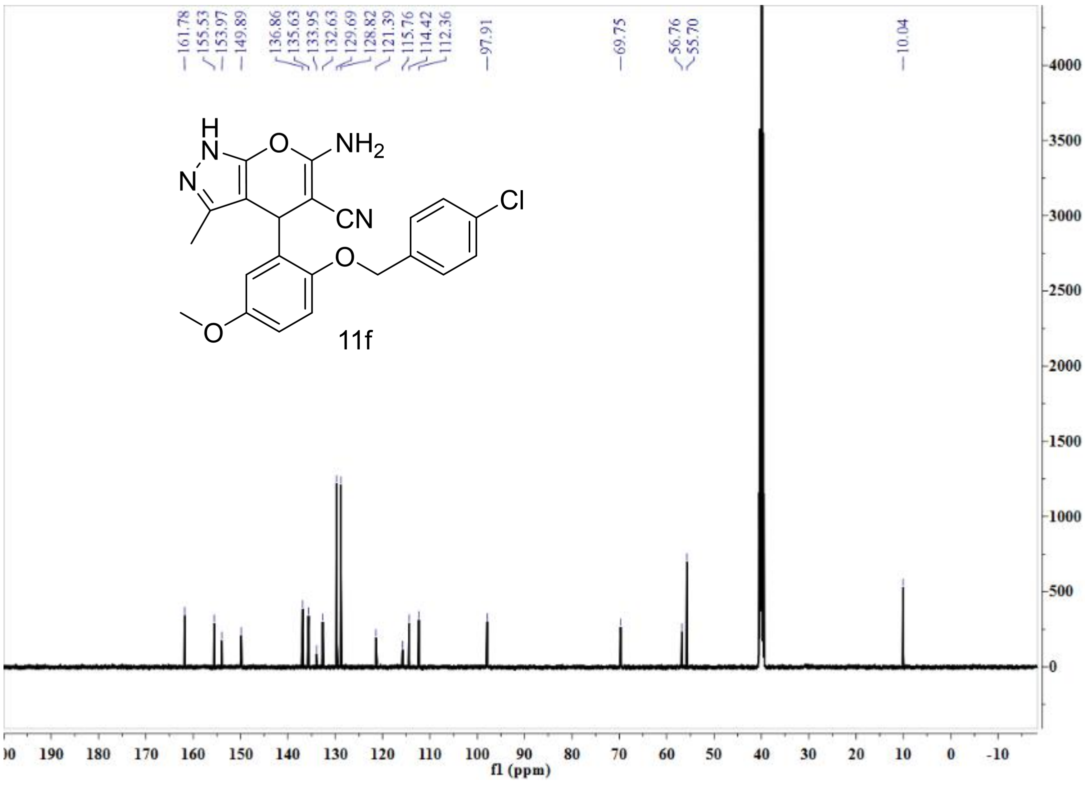
161.78
155.53
153.97
149.89
136.86
135.63
133.95
132.63
129.69
128.82
121.39
115.76
114.42
112.36
97.91
69.75
56.76
55.70
10.04
11f
f1 (ppm)

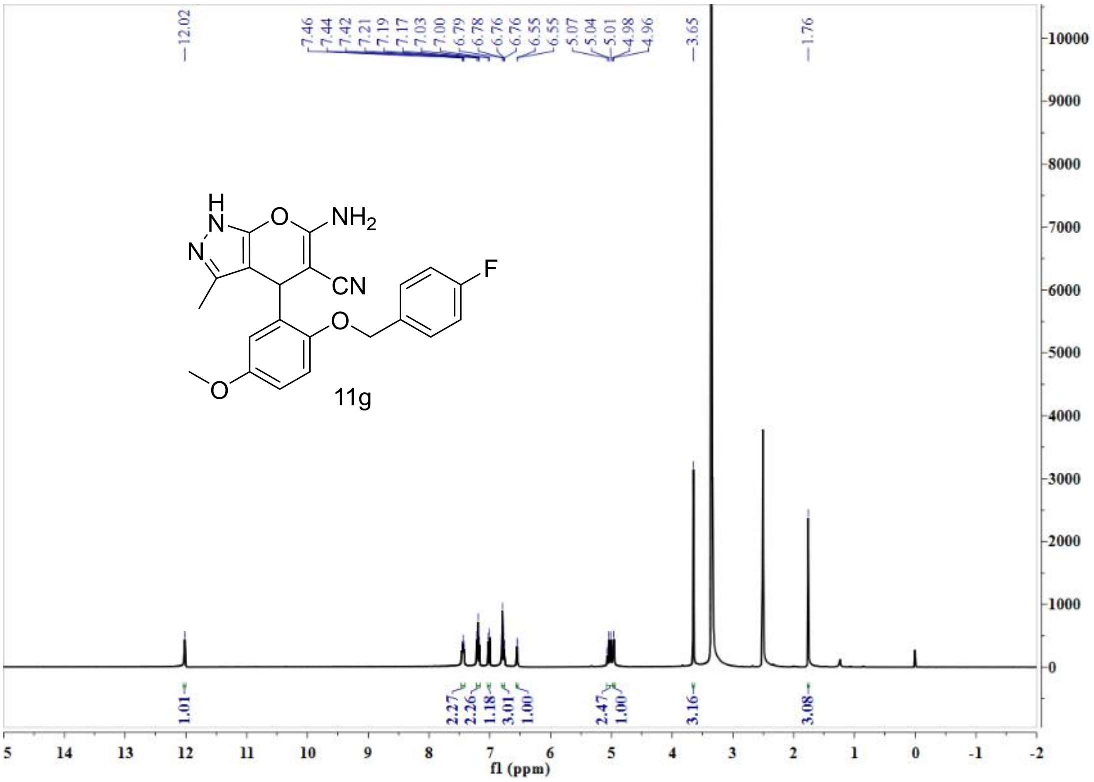
12.02
7.46
7.44
7.42
7.21
7.19
7.17
7.03
7.00
6.79
6.78
6.76
6.76
6.55
6.55
5.07
5.04
5.01
4.98
4.96
3.65
1.76
NH2
CN
F
11g
1.01
2.27
2.26
1.18
3.01
1.00
2.47
1.00
3.16
3.08
f1 (ppm)

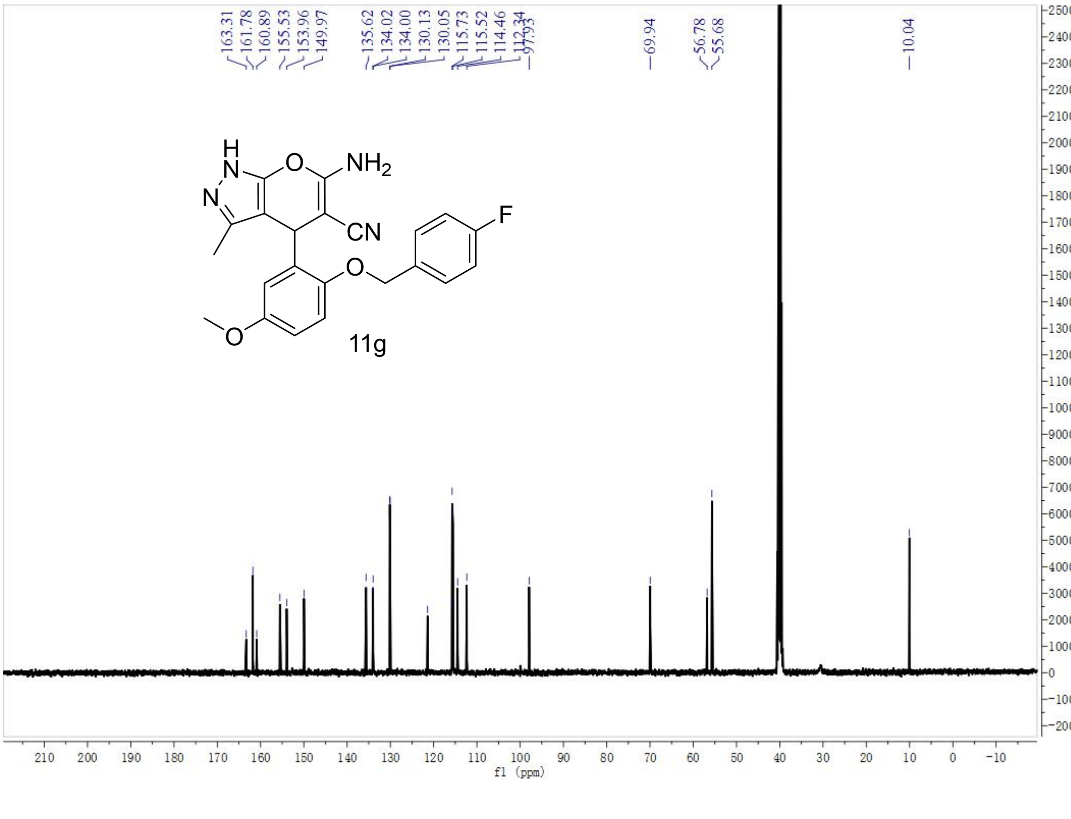
163.31
161.78
160.89
155.53
153.96
149.97
135.62
134.02
134.00
130.13
130.05
115.73
115.52
114.46
112.34
97.93
69.94
56.78
55.68
10.04
NH2
CN
F
11g
f1 (ppm)

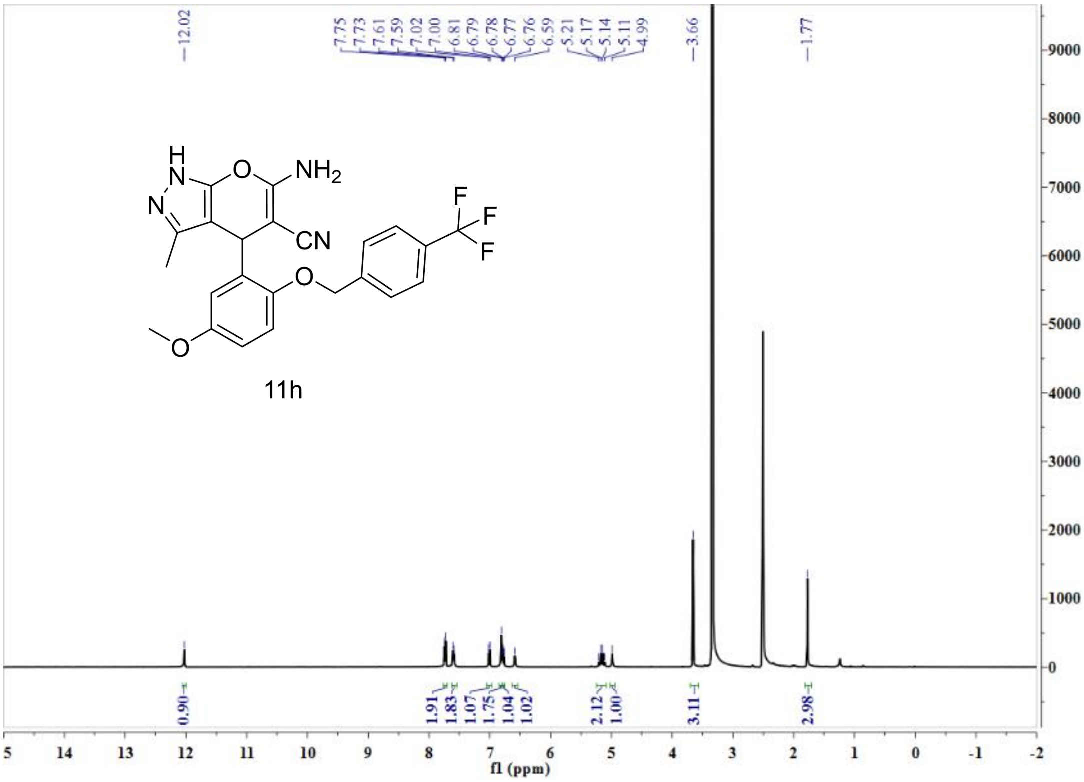
12.02
7.75 7.73 7.61 7.59 7.02 7.00 6.81 6.79 6.78 6.77 6.76 6.59 5.21 5.17 5.14 5.11 4.99
3.66
1.77
0.90 1.91 1.83 1.07 1.75 1.04 1.02 2.12 1.00 3.11 2.98
NH2
CN
11h
f1 (ppm)

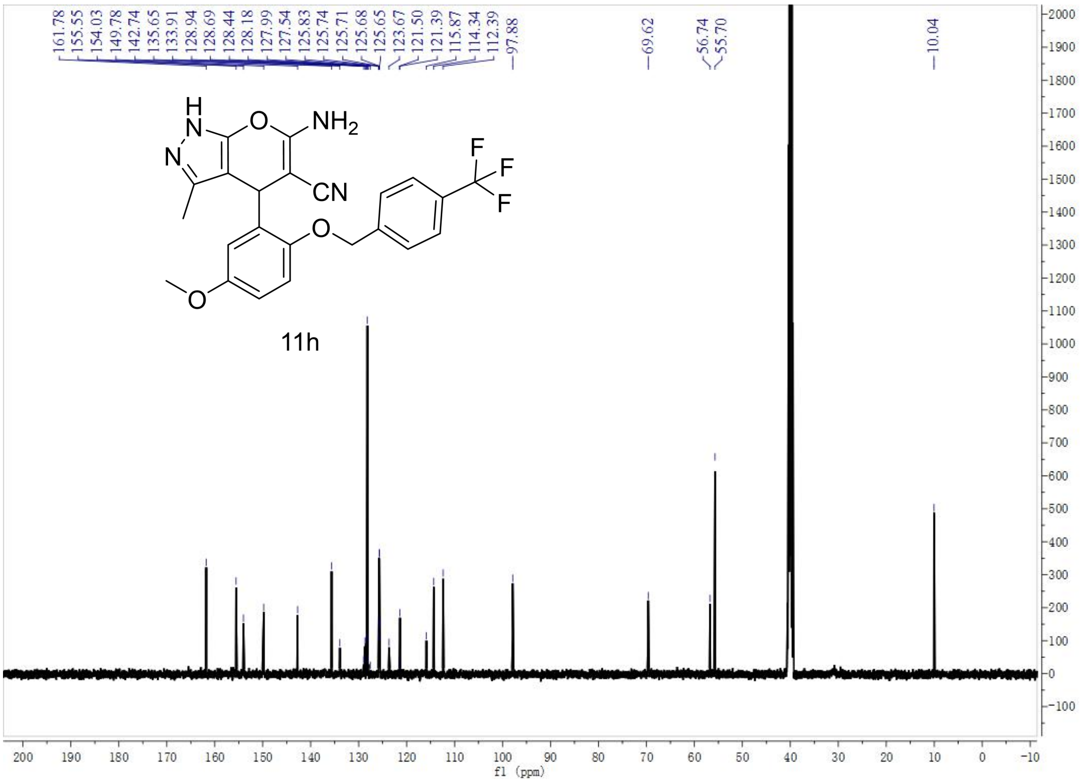
161.78 155.55 154.03 149.78 142.74 135.65 133.91 128.94 128.69 128.44 128.18 127.99 127.54 125.83 125.74 125.71 125.68 125.65 123.67 121.50 121.39 115.87 114.34 112.39 97.88
69.62
56.74 55.70
10.04
NH2
CN
11h
f1 (ppm)

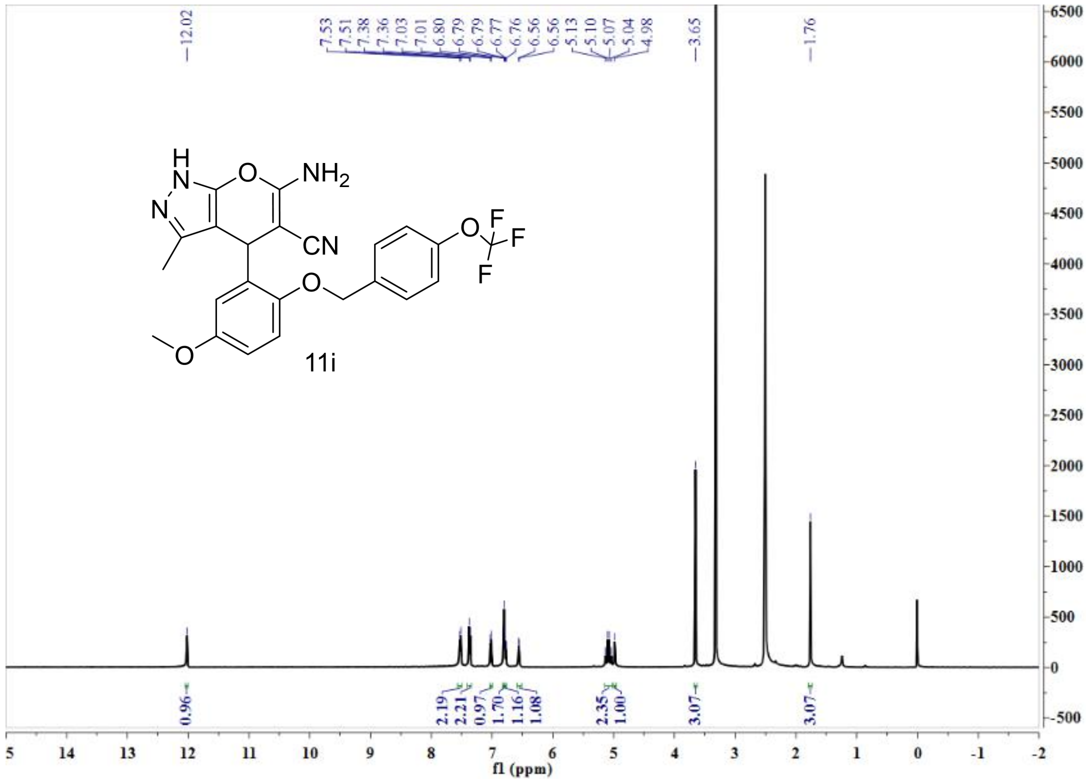
12.02
7.53 7.51 7.38 7.36 7.03 7.01 6.80 6.79 6.79 6.77 6.76 6.56 6.56 5.13 5.10 5.07 5.04 4.98
3.65
1.76
NH2
CN
O
F
11i
0.96 2.19 2.21 0.97 1.70 1.16 1.08 2.35 1.00 3.07 3.07
f1 (ppm)

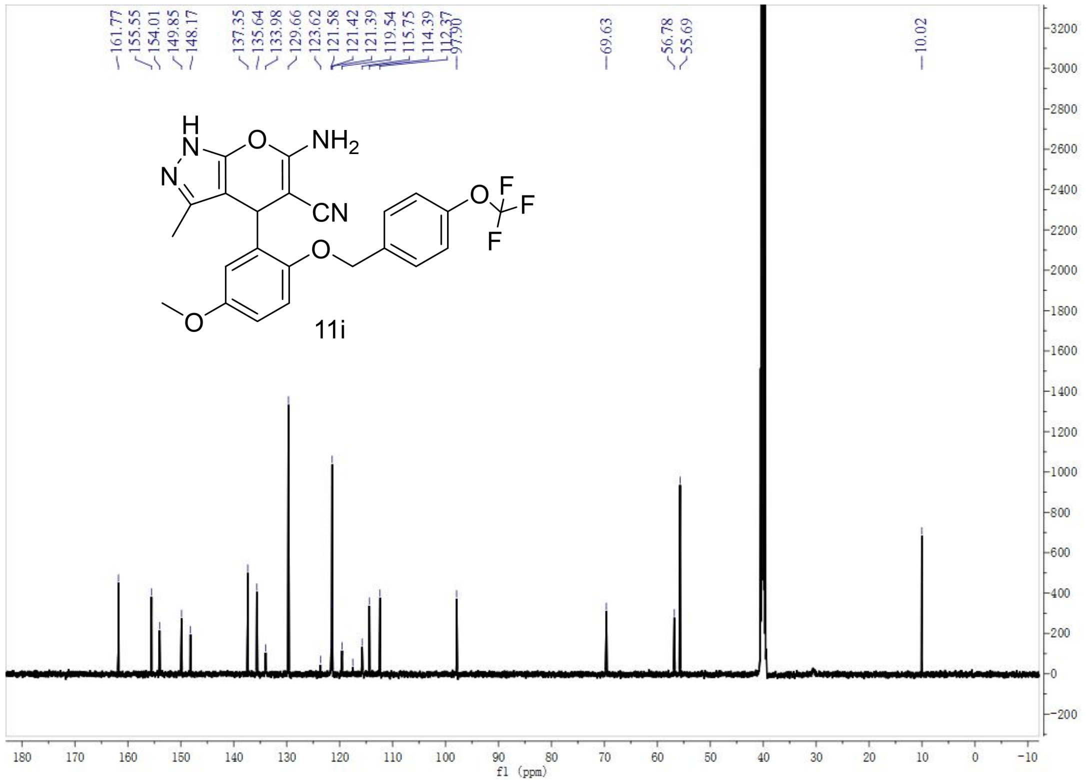
161.77 155.55 154.01 149.85 148.17 137.35 135.64 133.98 129.66 123.62 121.58 121.42 121.39 119.54 115.75 114.39 112.37 97.90
69.63
56.78 55.69
10.02
NH2
CN
11i
f1 (ppm)

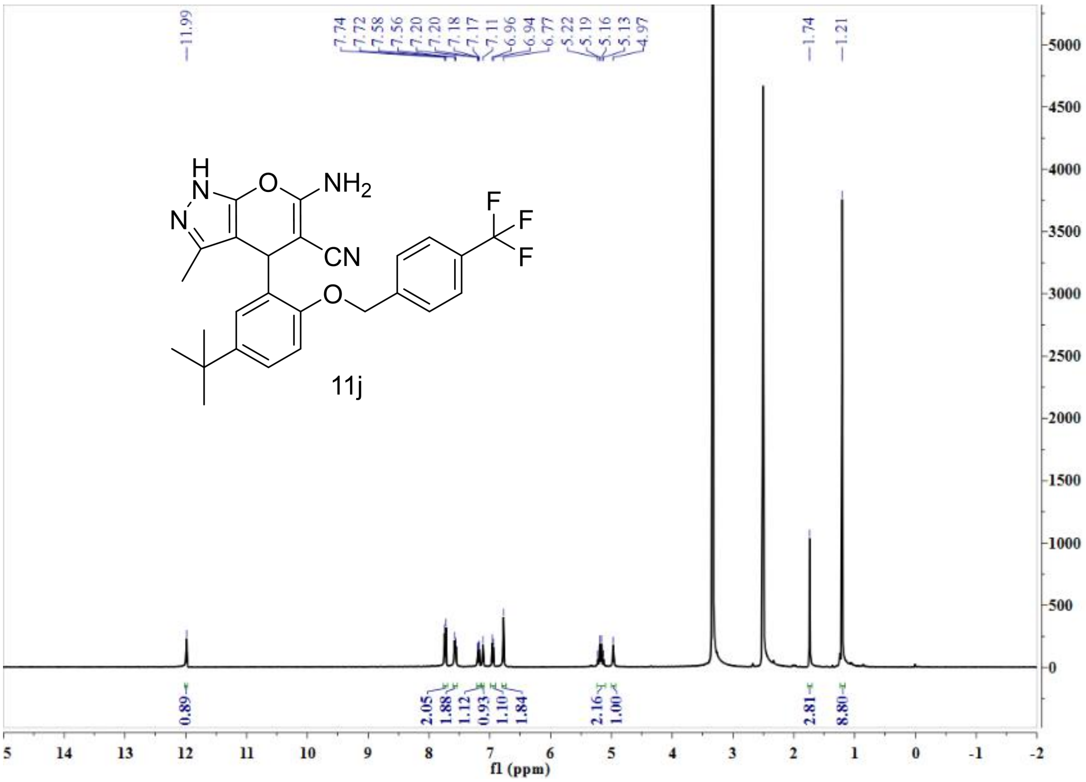
11.99
7.74 7.72 7.58 7.56 7.20 7.20 7.18 7.17 7.11 6.96 6.94 6.77 5.22 5.19 5.16 5.13 4.97
1.74
1.21
H N O NH2
N
CN
F F F
O
11j
0.89 2.05 1.88 1.12 0.93 1.10 1.84 2.16 1.00 2.81 8.80
f1 (ppm)

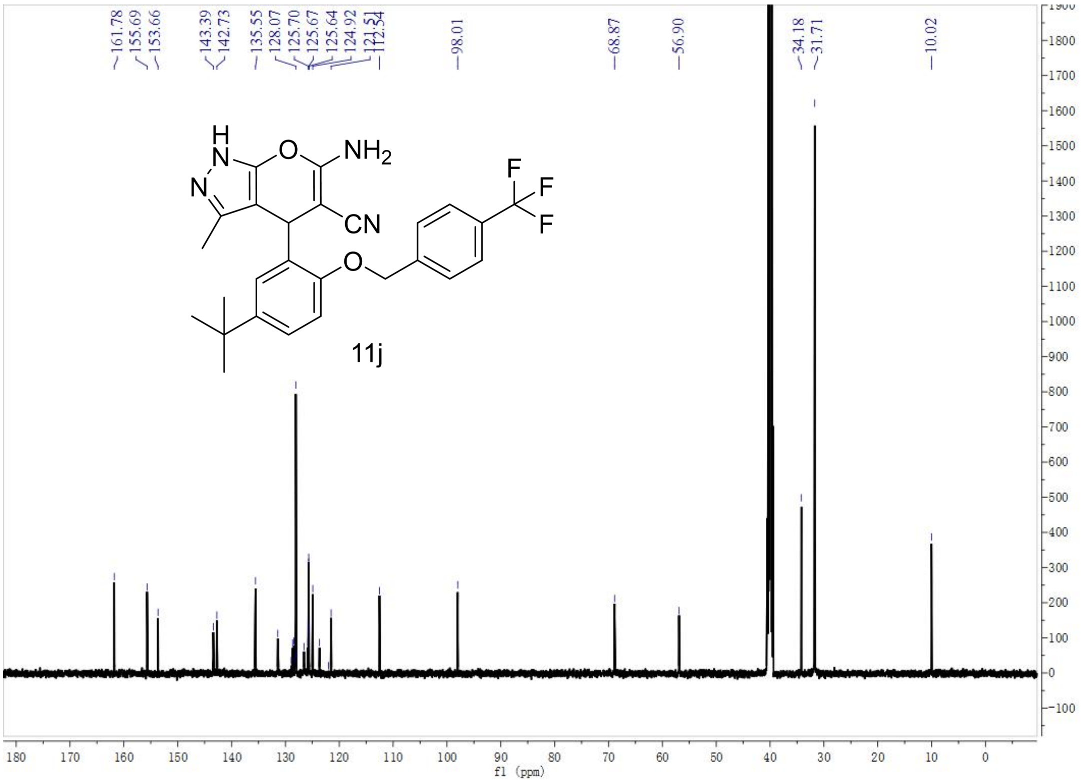
161.78 155.69 153.66 143.39 142.73 135.55 128.07 125.70 125.67 125.64 124.92 121.51 112.54
98.01
68.87
56.90
34.18 31.71
10.02
H N O NH2
N
CN
F F F
O
11j
f1 (ppm)

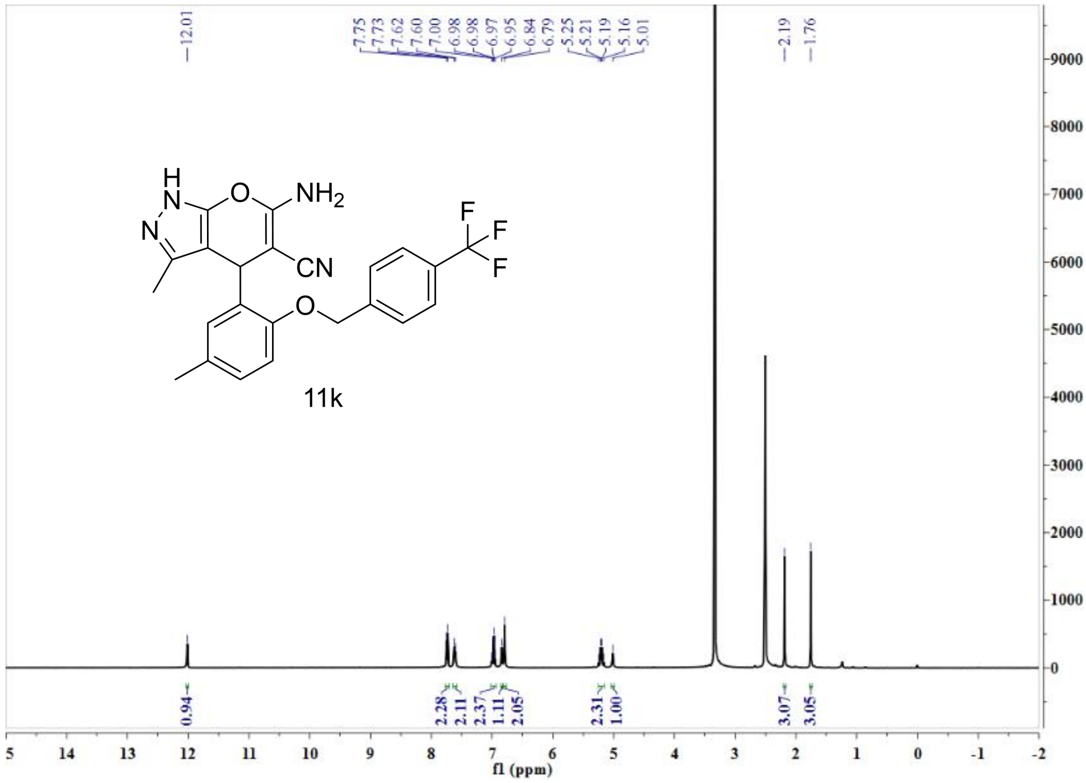
12.01
7.75 7.73 7.62 7.60 7.00 6.98 6.98 6.97 6.95 6.84 6.79 5.25 5.21 5.19 5.16 5.01
2.19 1.76
NH2
CN
F F F
11k
0.94 2.28 2.11 2.37 1.11 2.05 2.31 1.00 3.07 3.05
f1 (ppm)

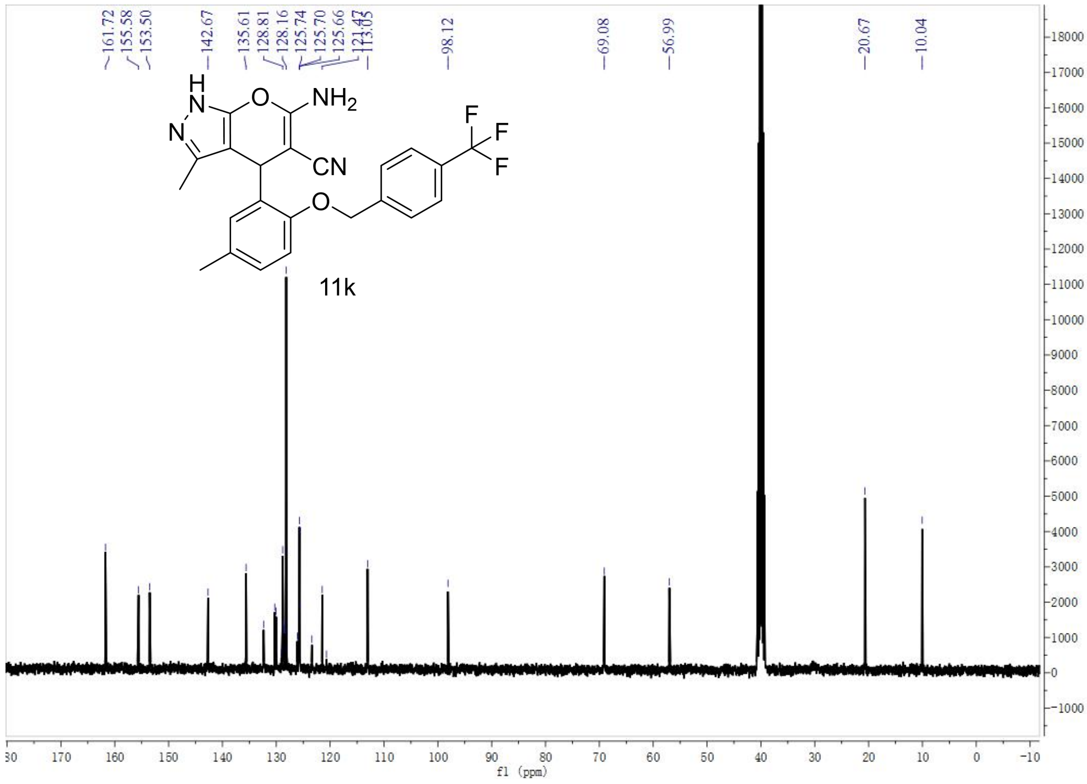
161.72 155.58 153.50 142.67 135.61 128.81 128.16 125.74 125.70 125.66 121.47 113.05 98.12 69.08 56.99 20.67 10.04
NH2
CN
F F F
11k
f1 (ppm)

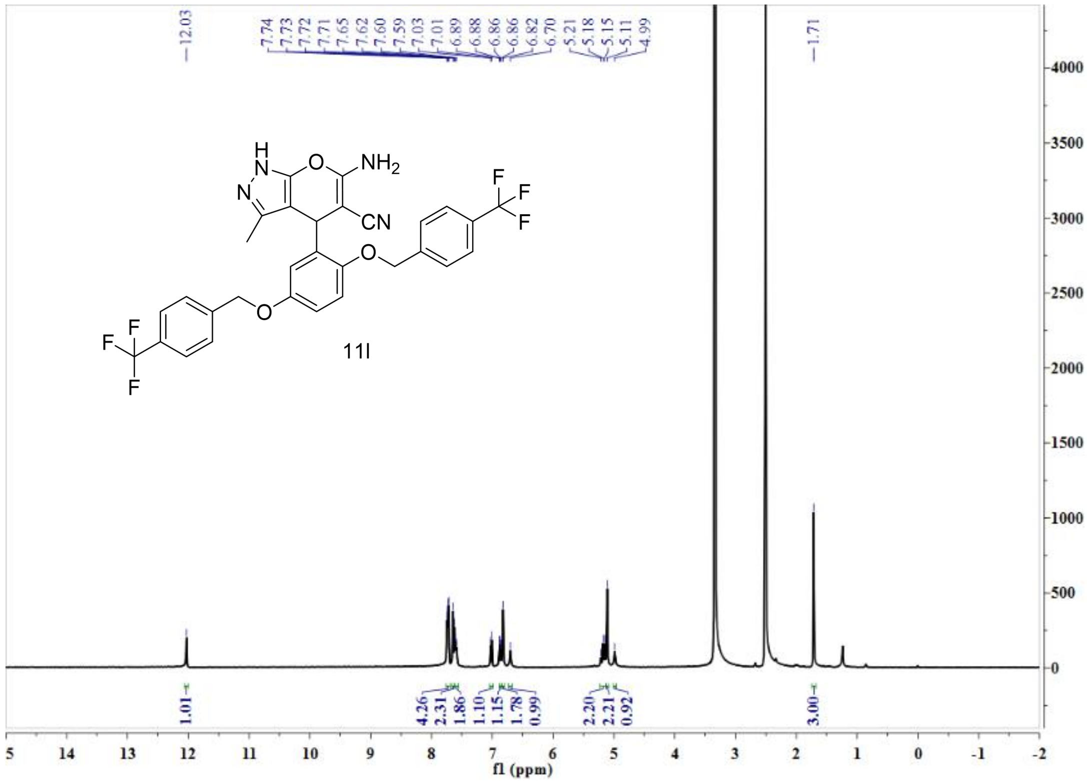
12.03
7.74
7.73
7.72
7.71
7.65
7.62
7.60
7.59
7.03
7.01
6.89
6.88
6.86
6.86
6.82
6.70
5.21
5.18
5.15
5.11
4.99
1.71
1.01
4.26
2.31
1.86
1.10
1.15
1.78
0.99
2.20
2.21
0.92
3.00
NH2
CN
F
O
N
11l
f1 (ppm)

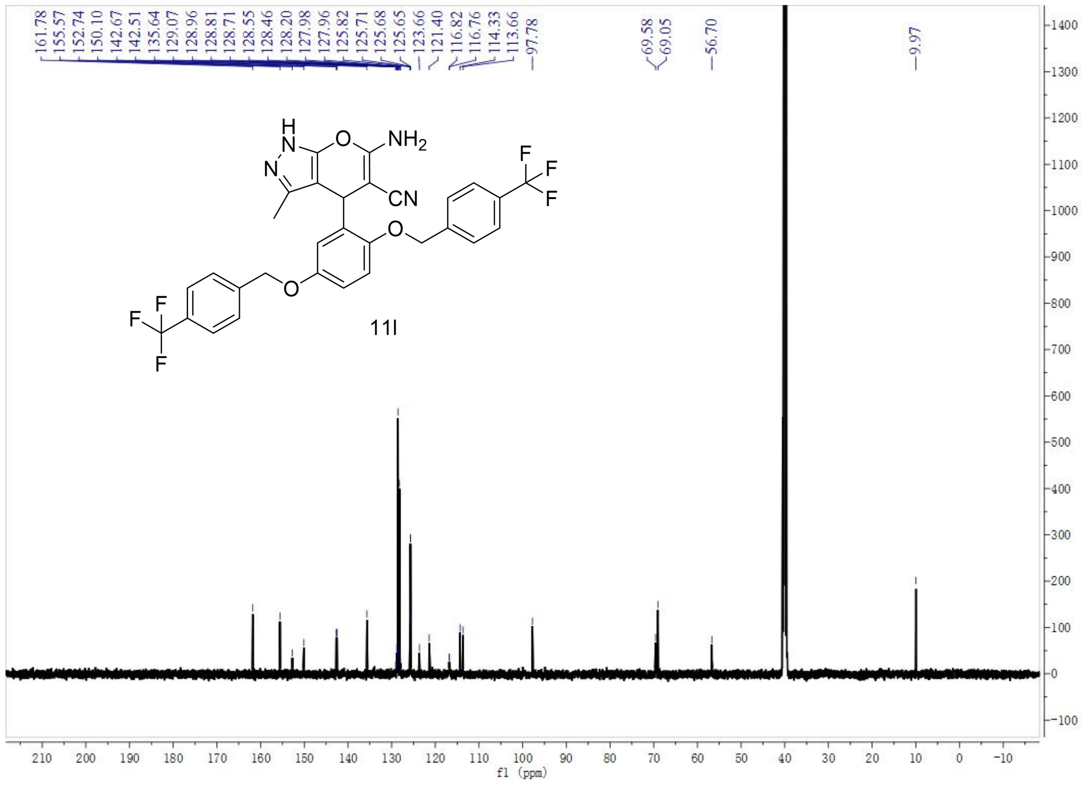
161.78
155.57
152.74
150.10
142.67
142.51
135.64
129.07
128.96
128.81
128.71
128.55
128.46
128.20
127.98
127.96
125.82
125.71
125.68
125.65
123.66
121.40
116.82
116.76
114.33
113.66
97.78
69.58
69.05
56.70
9.97
NH2
CN
11l
f1 (ppm)

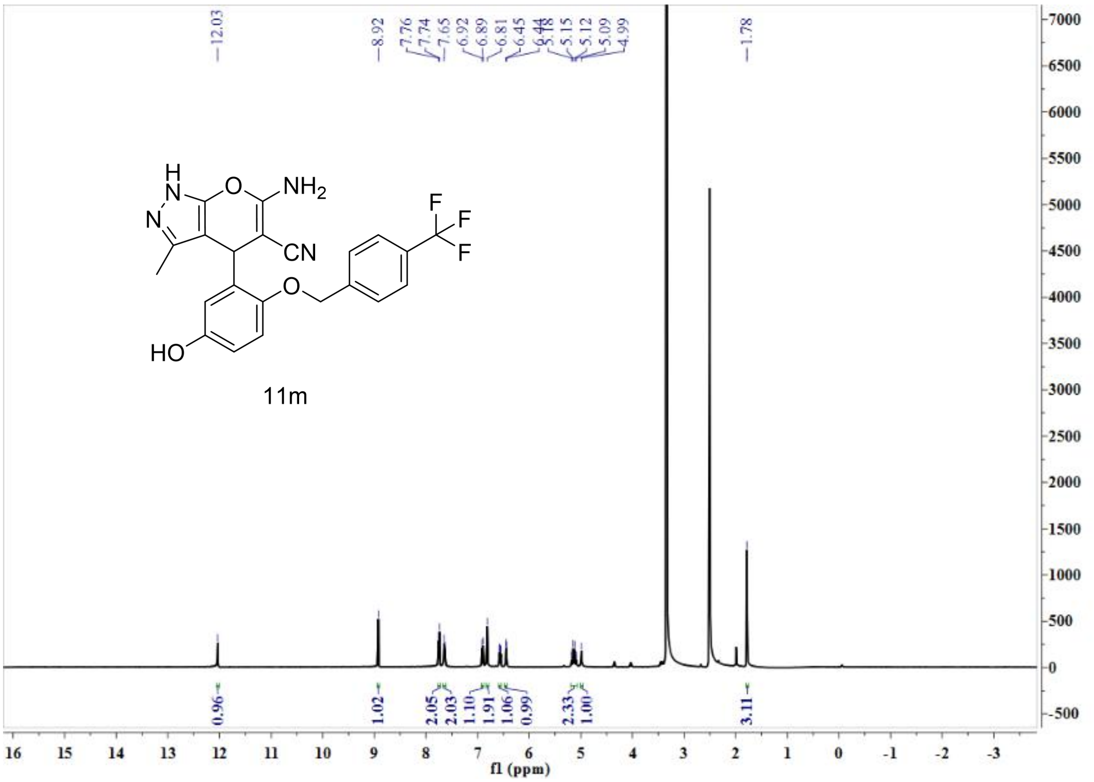

12.03
8.92
7.76
7.74
7.65
6.92
6.89
6.81
6.45
6.44
5.18
5.15
5.12
5.09
4.99
1.78
0.96
1.02
2.05
2.03
1.10
1.91
1.06
0.99
2.33
1.00
3.11
NH2
CN
F
F
F
O
HO
11m
f1 (ppm)

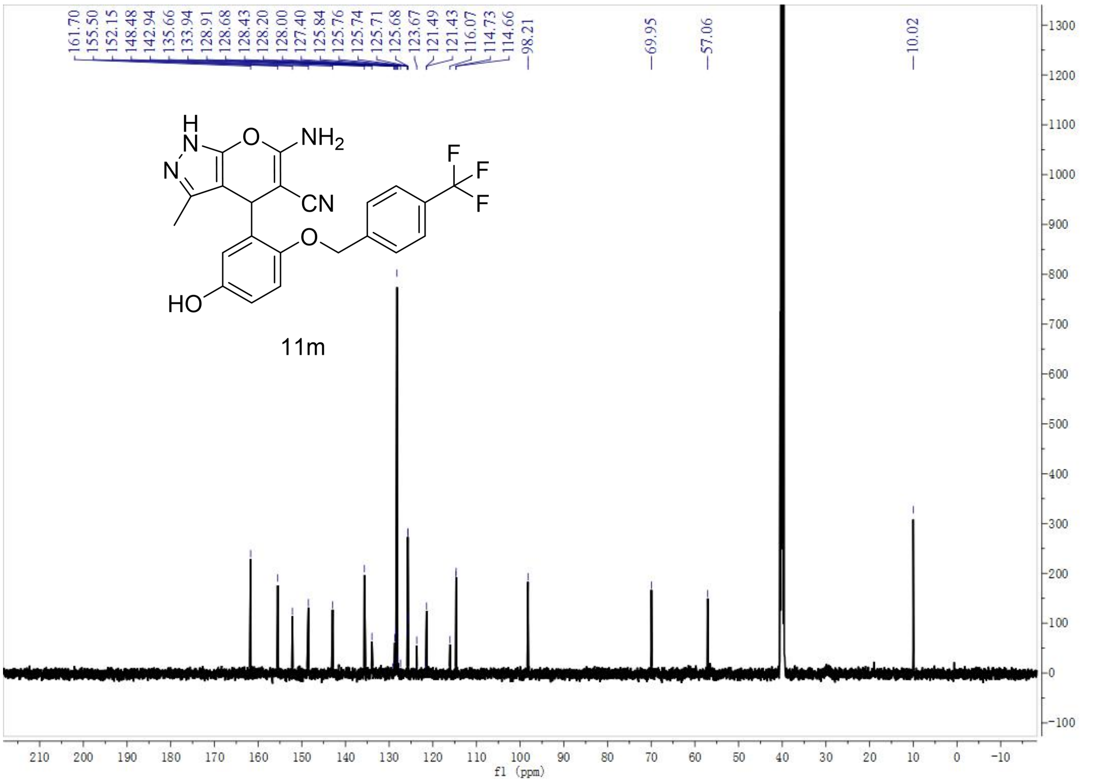

161.70
155.50
152.15
148.48
142.94
135.66
133.94
128.91
128.68
128.43
128.20
128.00
127.40
125.84
125.76
125.74
125.71
125.68
123.67
121.49
121.43
116.07
114.73
114.66
98.21
69.95
57.06
10.02
11m
f1 (ppm)

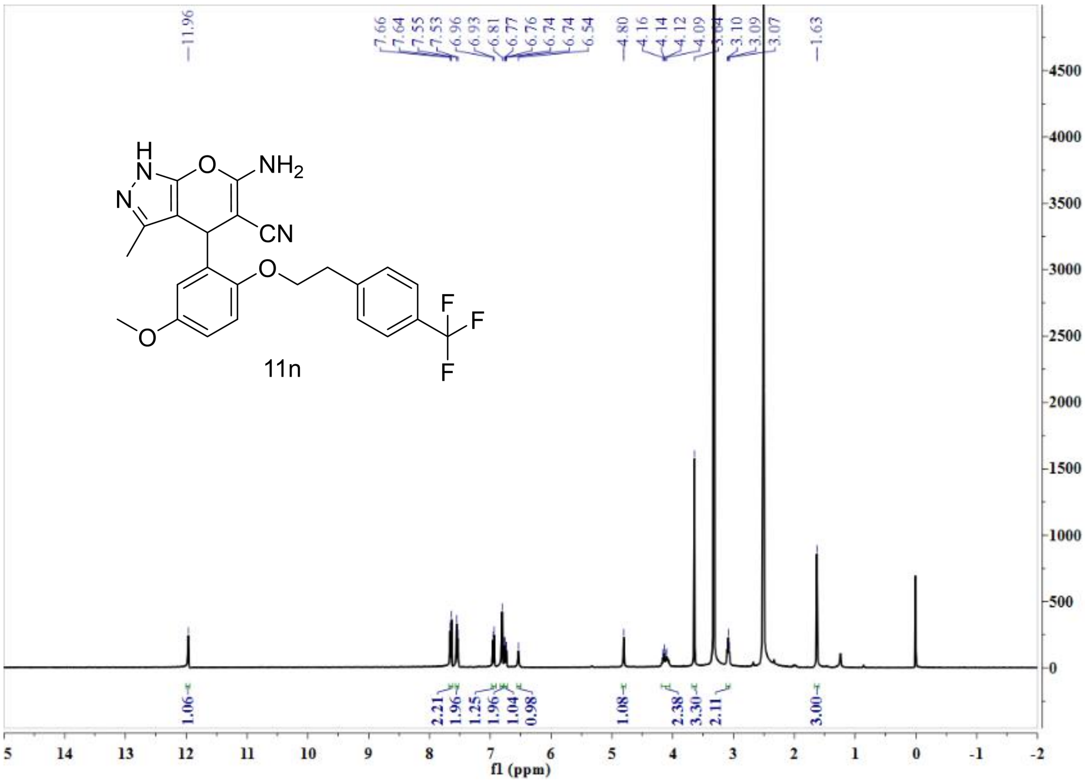
11.96
7.66 7.64 7.55 7.53 6.96 6.93 6.81 6.77 6.76 6.74 6.74 6.54
4.80 4.16 4.14 4.12 4.09 3.64 3.10 3.09 3.07 1.63
1.06 2.21 1.96 1.25 1.96 1.04 0.98 1.08 2.38 3.30 2.11 3.00
NH2
CN
O
N
H
F
11n
f1 (ppm)

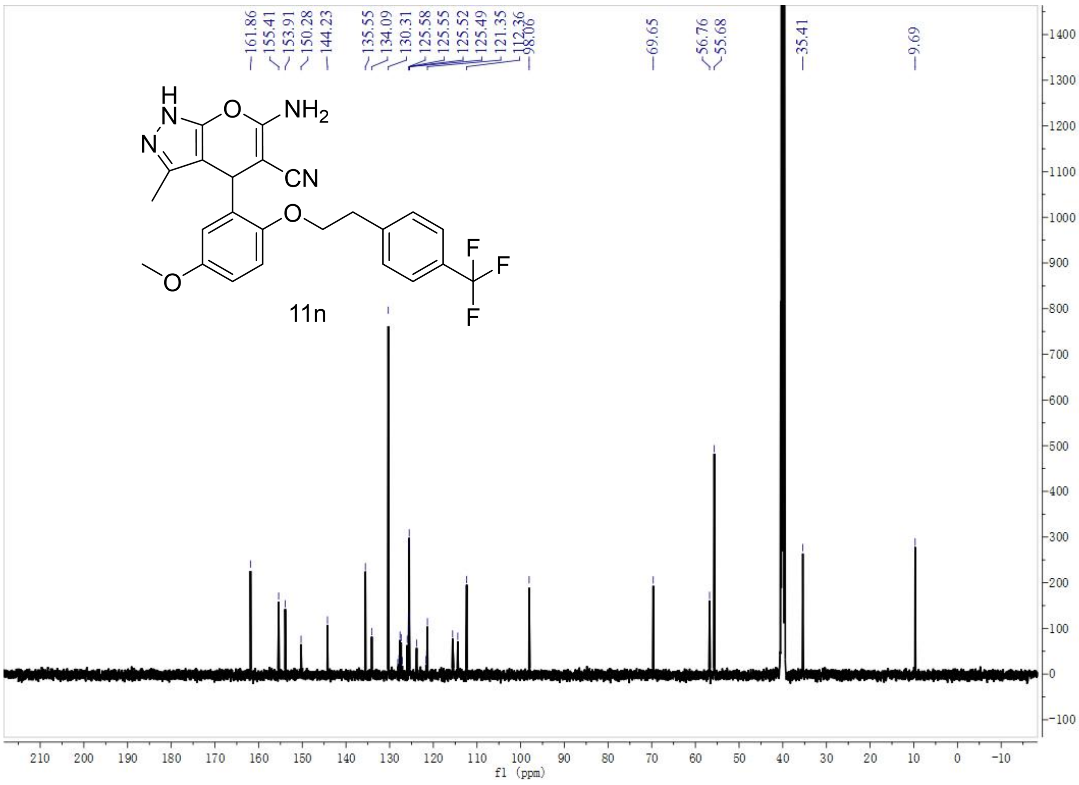
161.86 155.41 153.91 150.28 144.23 135.55 134.09 130.31 125.58 125.55 125.52 125.49 121.35 112.36 98.06 69.65 56.76 55.68 35.41 9.69
NH2
CN
O
N
H
F
11n
f1 (ppm)

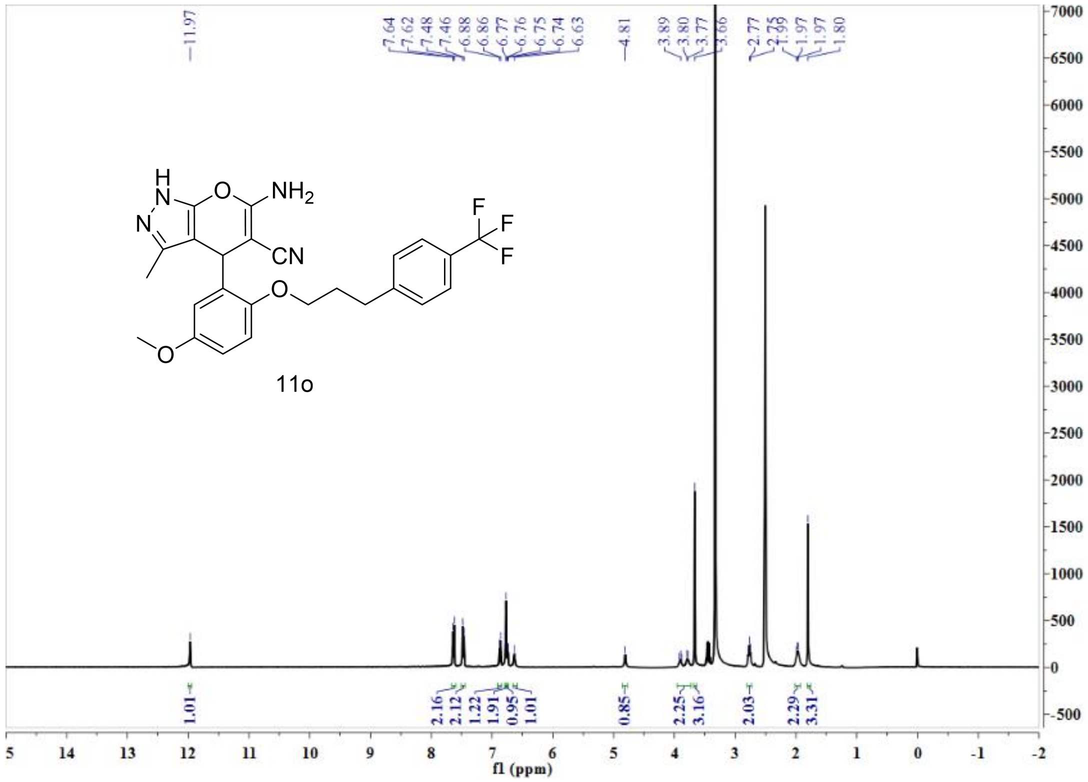
11.97
7.64 7.62 7.48 7.46 6.88 6.86 6.77 6.76 6.75 6.74 6.63
4.81
3.89 3.80 3.77 3.66
2.77 2.75 1.99 1.97 1.97 1.80
NH2
CN
F
F
F
O
N
H
11o
1.01 2.16 2.12 1.22 1.91 0.95 1.01 0.85 2.25 3.16 2.03 2.29 3.31
f1 (ppm)

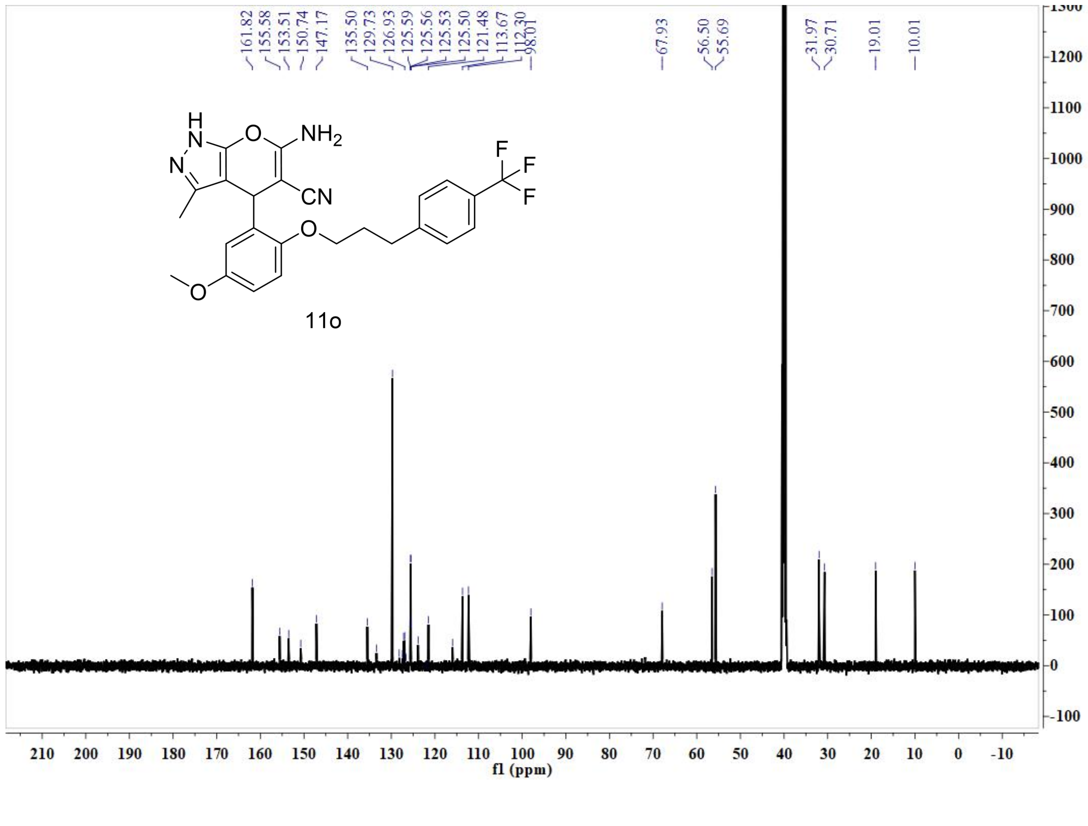
161.82 155.58 153.51 150.74 147.17 135.50 129.73 126.93 125.59 125.56 125.53 125.50 121.48 113.67 112.30 98.01
67.93
56.50 55.69
31.97 30.71
19.01
10.01
NH2
CN
F
F
F
11o
f1 (ppm)

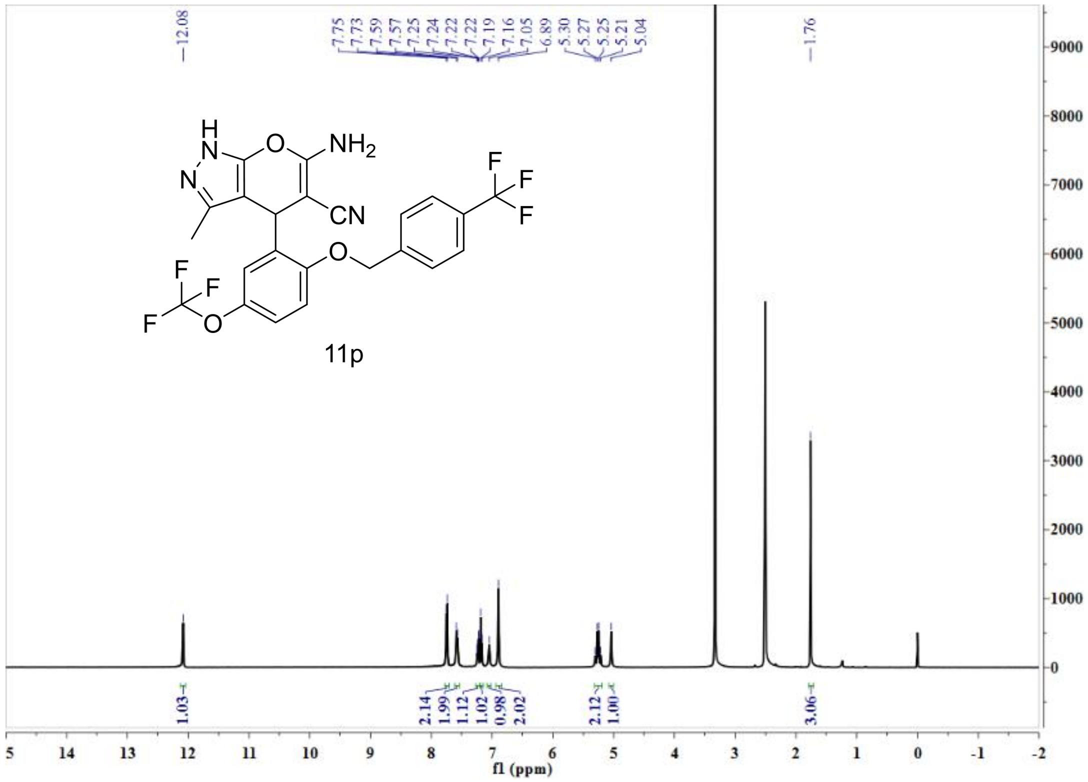
12.08
7.75 7.73 7.59 7.57 7.25 7.24 7.22 7.22 7.19 7.16 7.05 6.89 5.30 5.27 5.25 5.21 5.04
1.76
NH2
CN
11p
1.03 2.14 1.99 1.12 1.02 0.98 2.02 2.12 1.00 3.06
f1 (ppm)

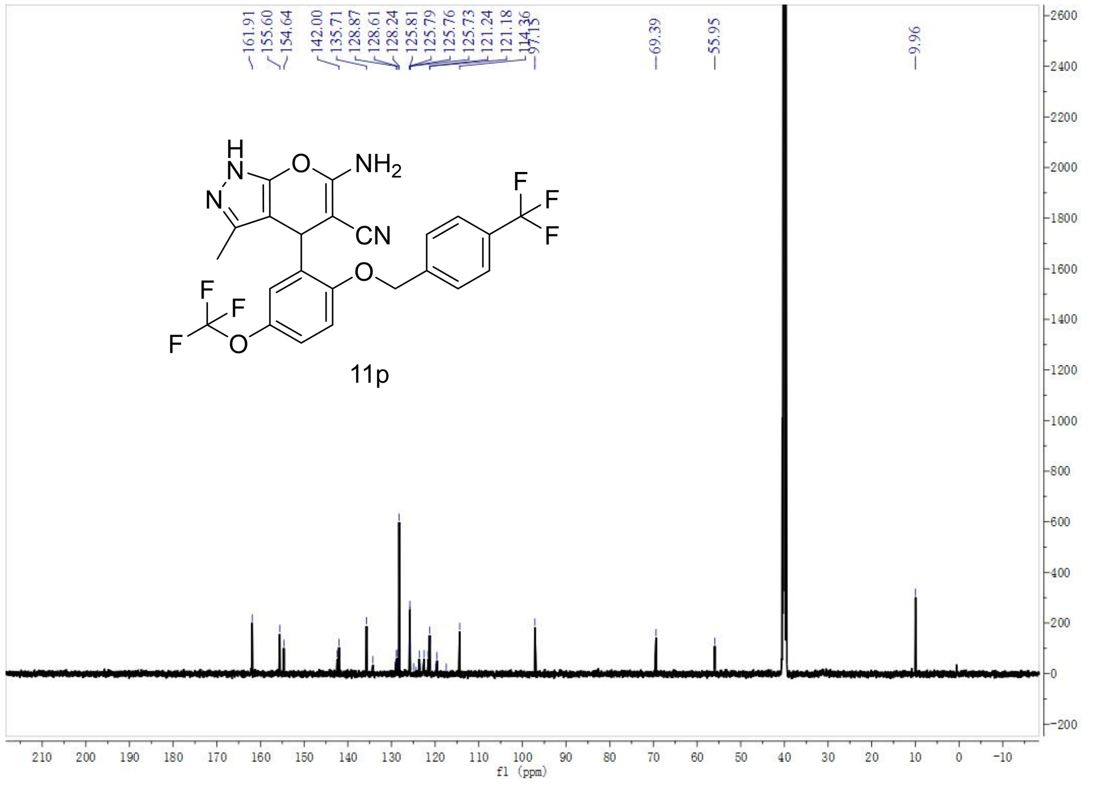
161.91 155.60 154.64 142.00 135.71 128.87 128.61 128.24 125.81 125.79 125.76 125.73 121.24 121.18 114.36 97.15
69.39
55.95
9.96
NH2
CN
11p
f1 (ppm)

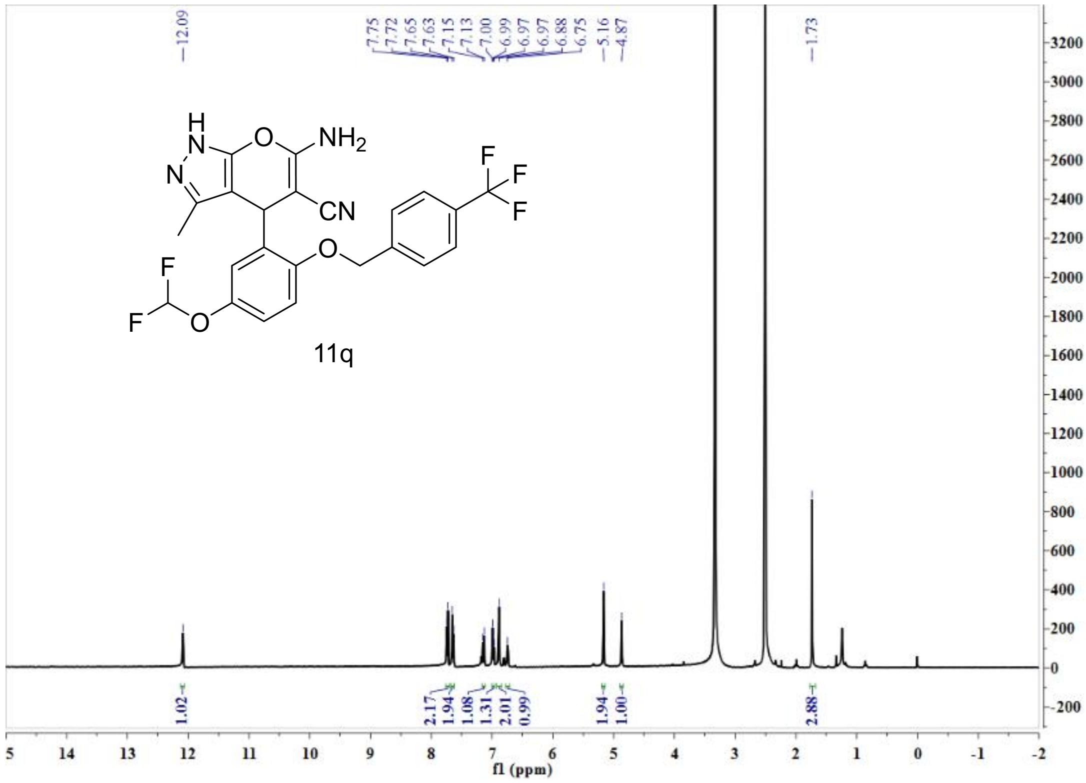
12.09
7.75
7.72
7.65
7.63
7.15
7.13
7.00
6.99
6.97
6.97
6.88
6.75
5.16
4.87
1.73
NH2
CN
O
F
11q
1.02
2.17
1.94
1.08
1.31
2.01
0.99
1.94
1.00
2.88
f1 (ppm)

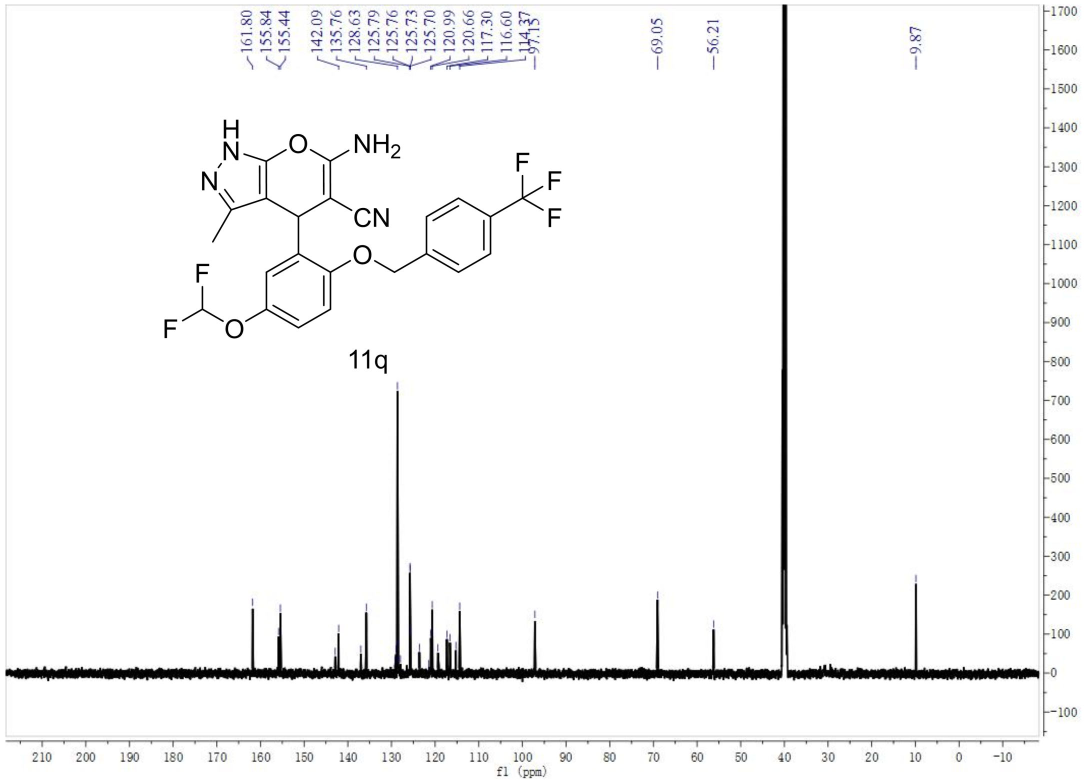
161.80
155.84
155.44
142.09
135.76
128.63
125.79
125.76
125.73
125.70
120.99
120.66
117.30
116.60
114.37
97.15
69.05
56.21
9.87
NH2
CN
11q
f1 (ppm)

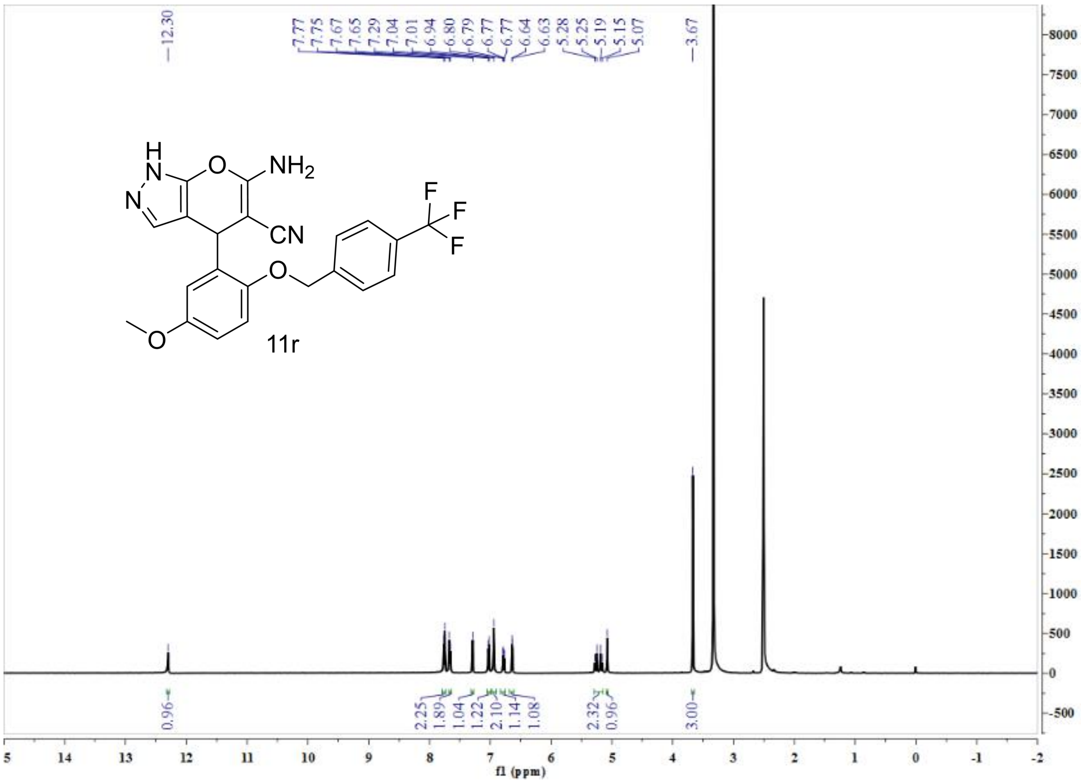
12.30
7.77
7.75
7.67
7.65
7.29
7.04
7.01
6.94
6.80
6.79
6.77
6.77
6.64
6.63
5.28
5.25
5.19
5.15
5.07
3.67
0.96
2.25
1.89
1.04
1.22
2.10
1.14
1.08
2.32
0.96
3.00
11r
f1 (ppm)

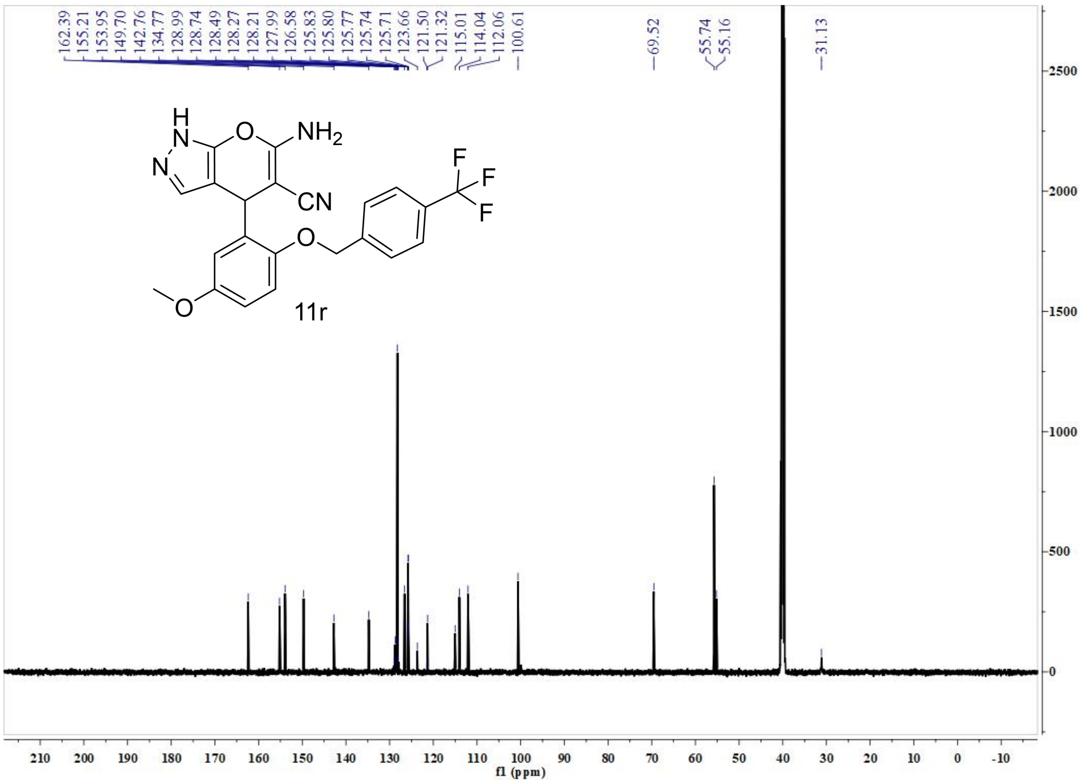
162.39
155.21
153.95
149.70
142.76
134.77
128.99
128.74
128.49
128.27
128.21
127.99
126.58
125.83
125.80
125.77
125.74
125.71
123.66
121.50
121.32
115.01
114.04
112.06
100.61
69.52
55.74
55.16
31.13
11r
f1 (ppm)

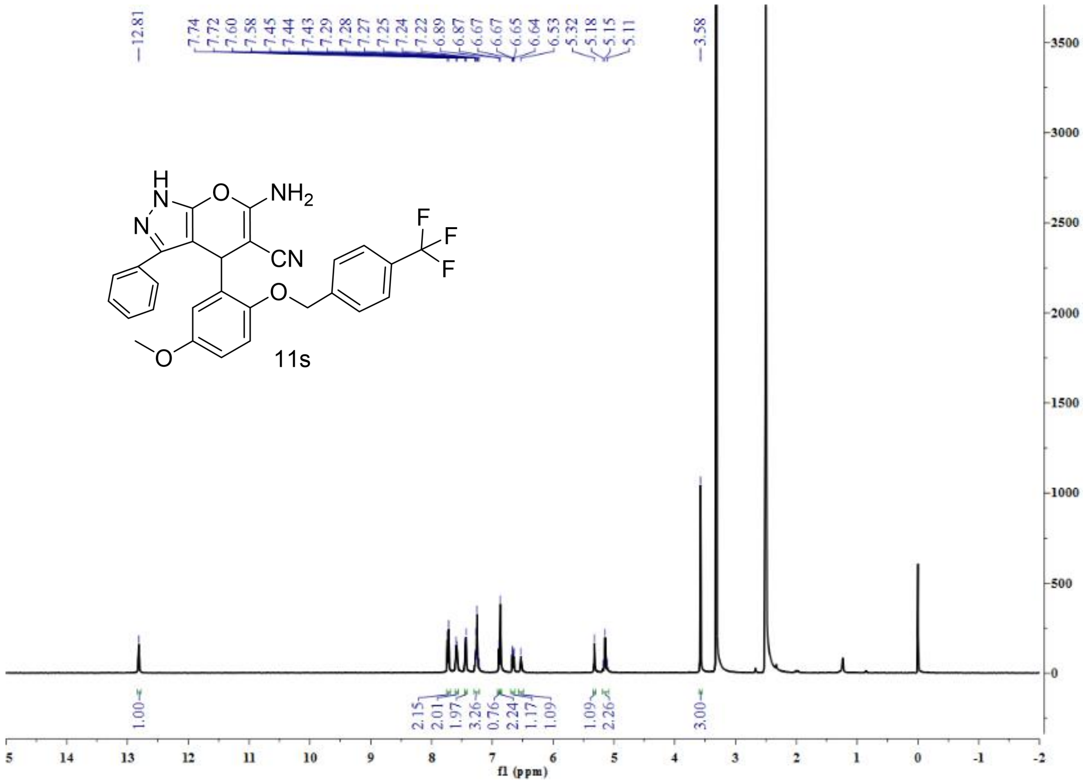

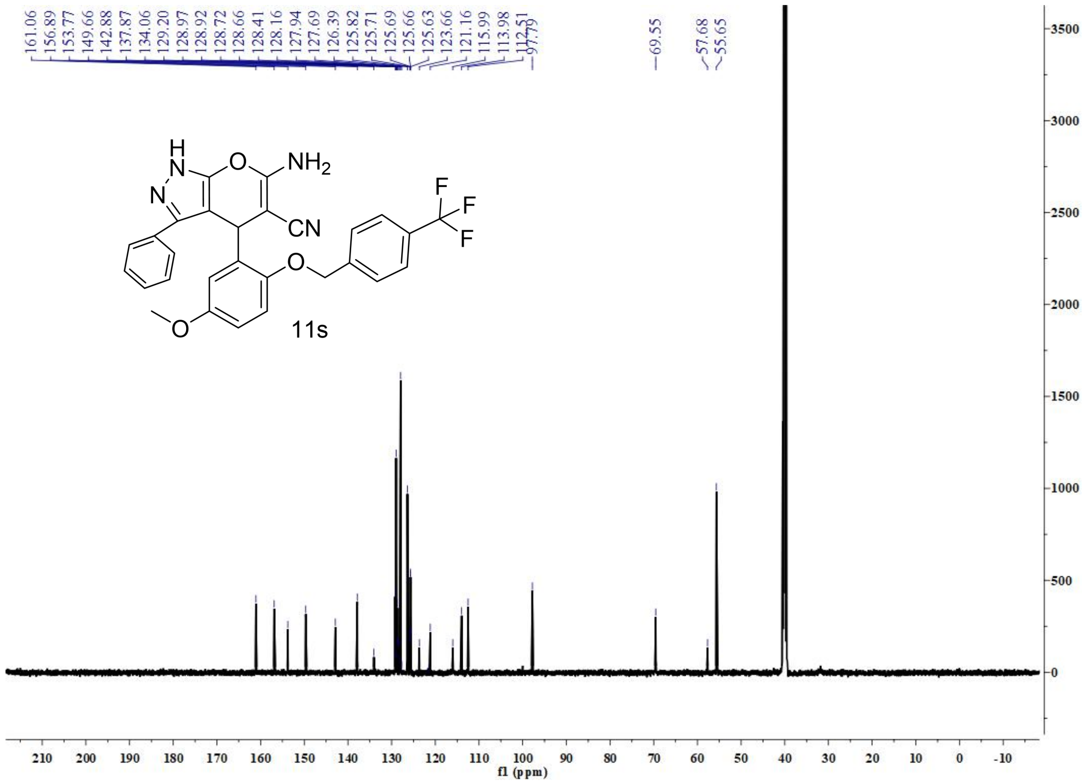

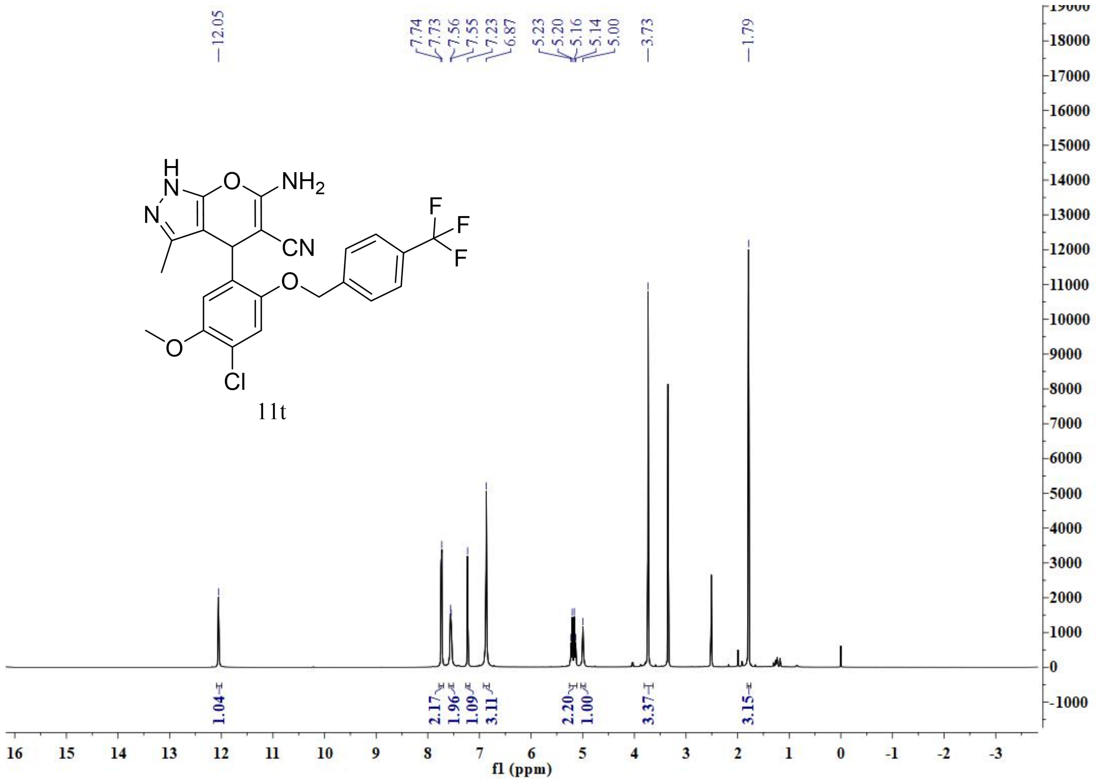

12.05
7.74
7.73
7.56
7.55
7.23
6.87
5.23
5.20
5.16
5.14
5.00
3.73
1.79
11t
1.04
2.17
1.96
1.09
3.11
2.20
1.00
3.37
3.15
fl (ppm)

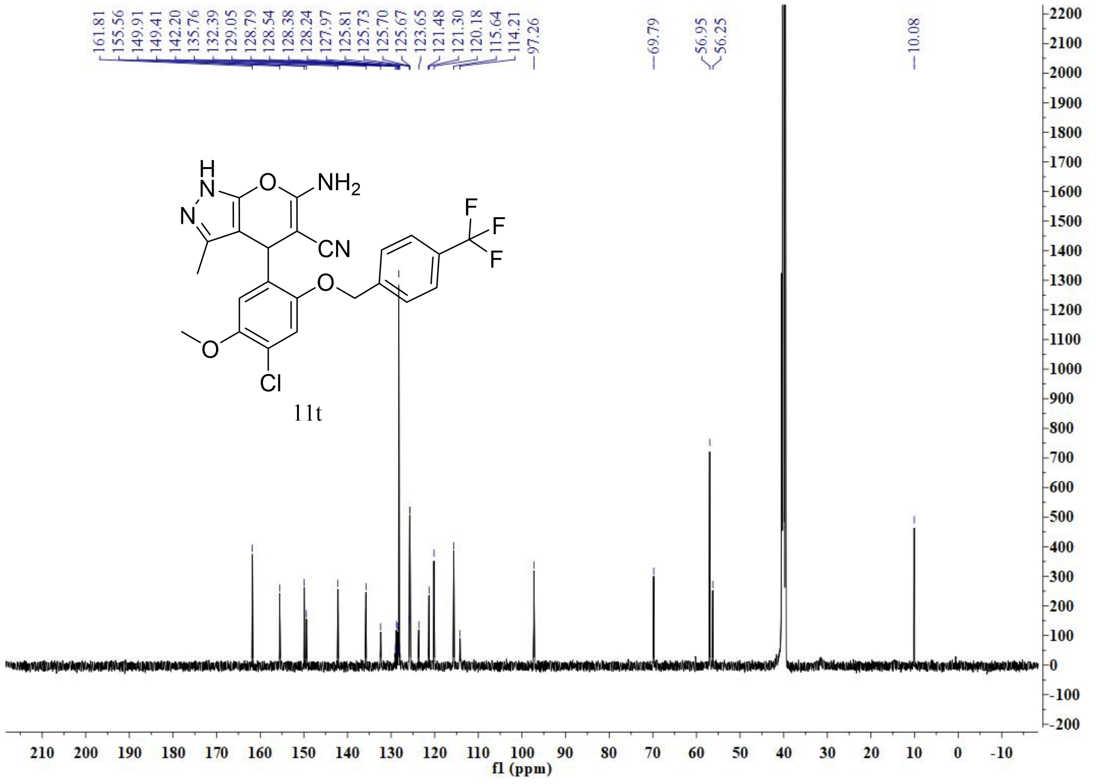

161.81
155.56
149.91
149.41
142.20
135.76
132.39
129.05
128.79
128.54
128.38
128.24
127.97
125.81
125.73
125.70
125.67
123.65
121.48
121.30
120.18
115.64
114.21
97.26
69.79
56.95
56.25
10.08
11t
fl (ppm)

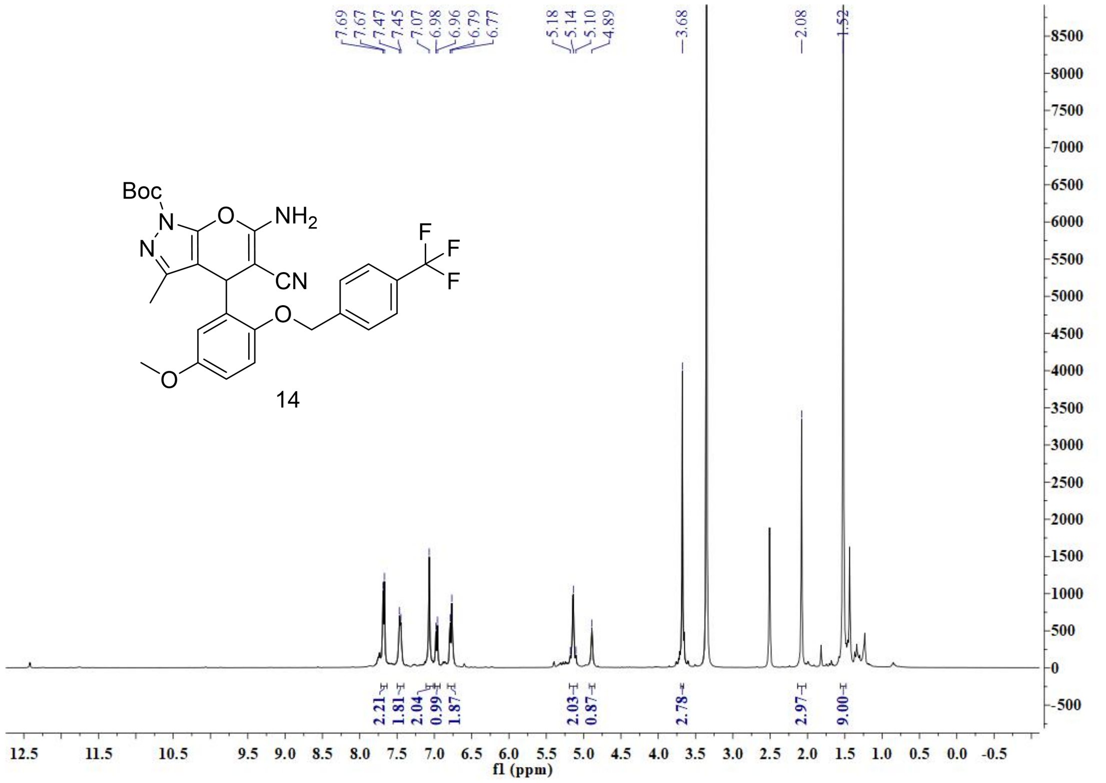

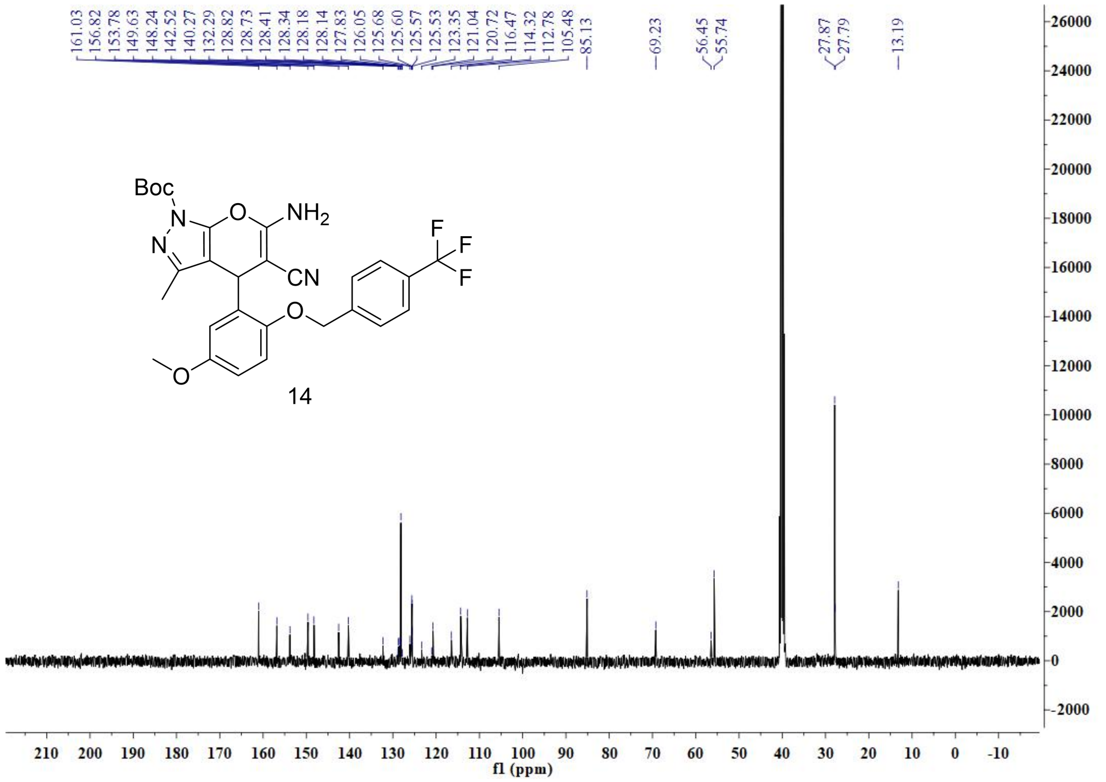

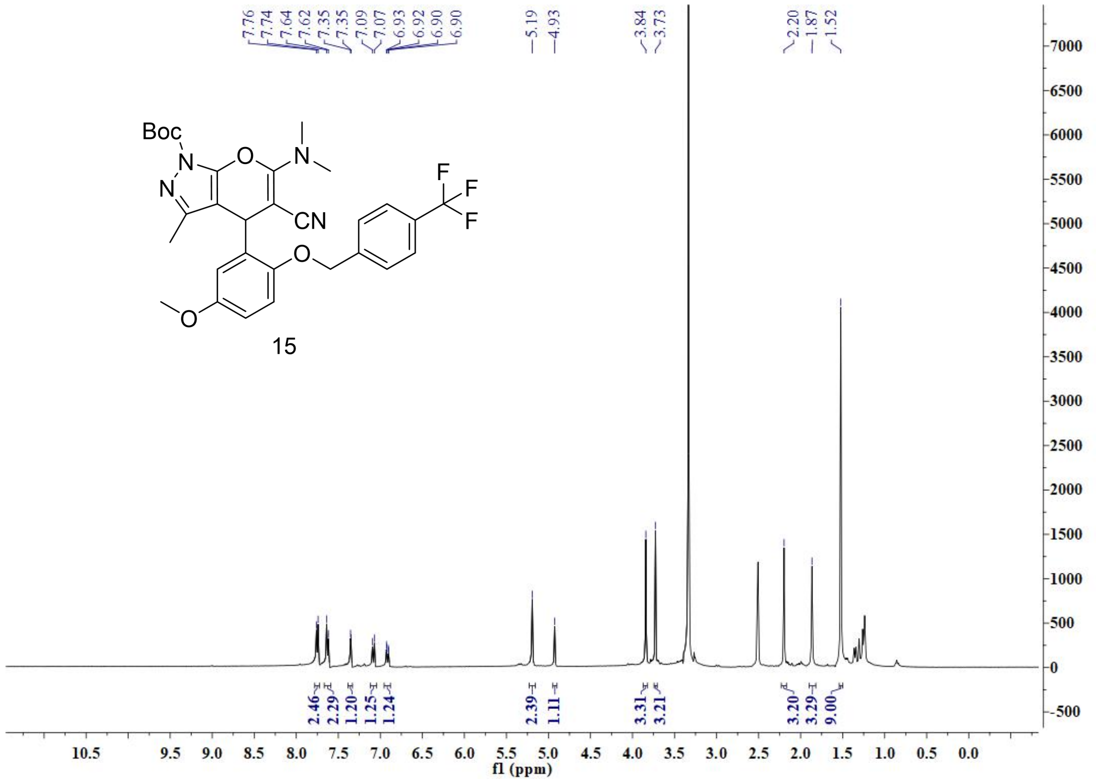
7.76
7.74
7.64
7.62
7.35
7.35
7.09
7.07
6.93
6.92
6.90
6.90
5.19
4.93
3.84
3.73
2.20
1.87
1.52
Boc
N
N
O
N
CN
F
F
F
O
O
15
2.46
2.29
1.20
1.25
1.24
2.39
1.11
3.31
3.21
3.20
3.29
9.00
fl (ppm)

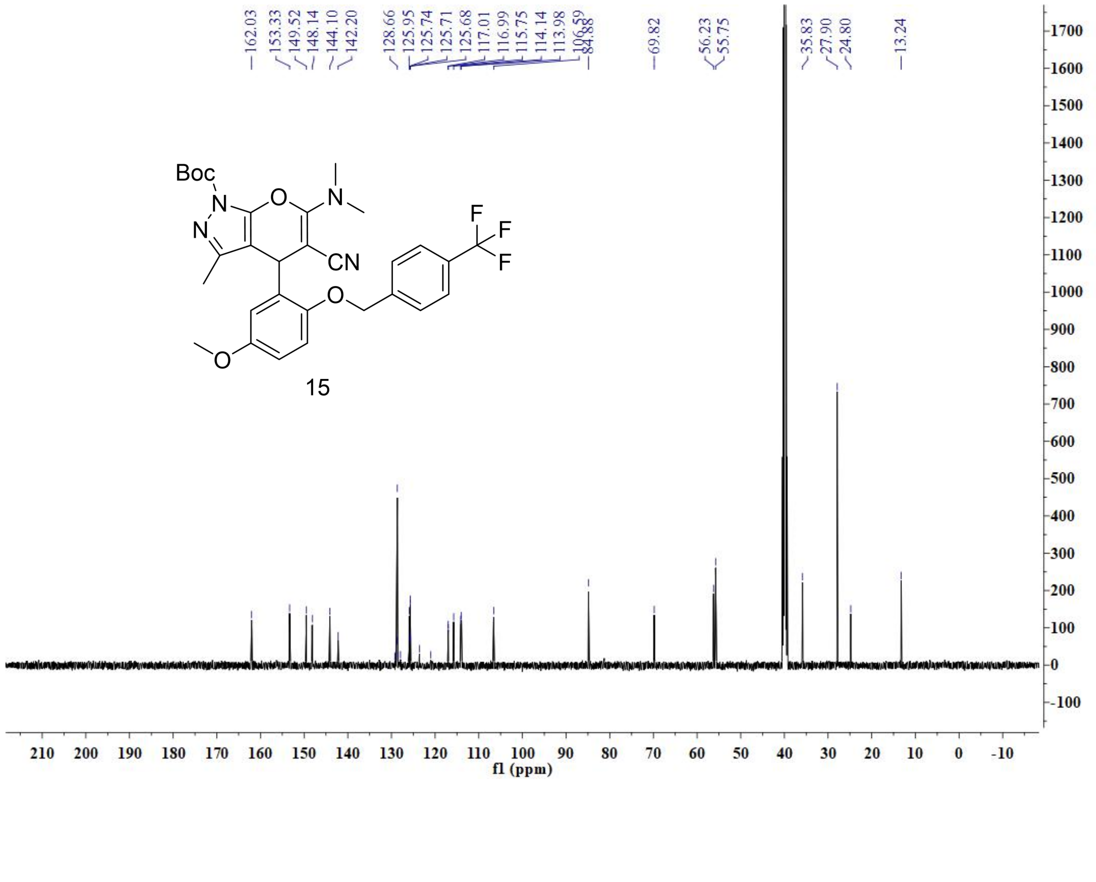
162.03
153.33
149.52
148.14
144.10
142.20
128.66
125.95
125.74
125.71
125.68
117.01
116.99
115.75
114.14
113.98
106.59
84.88
69.82
56.23
55.75
35.83
27.90
24.80
13.24
Boc
N
N
O
N
CN
F
F
F
O
O
15
fl (ppm)

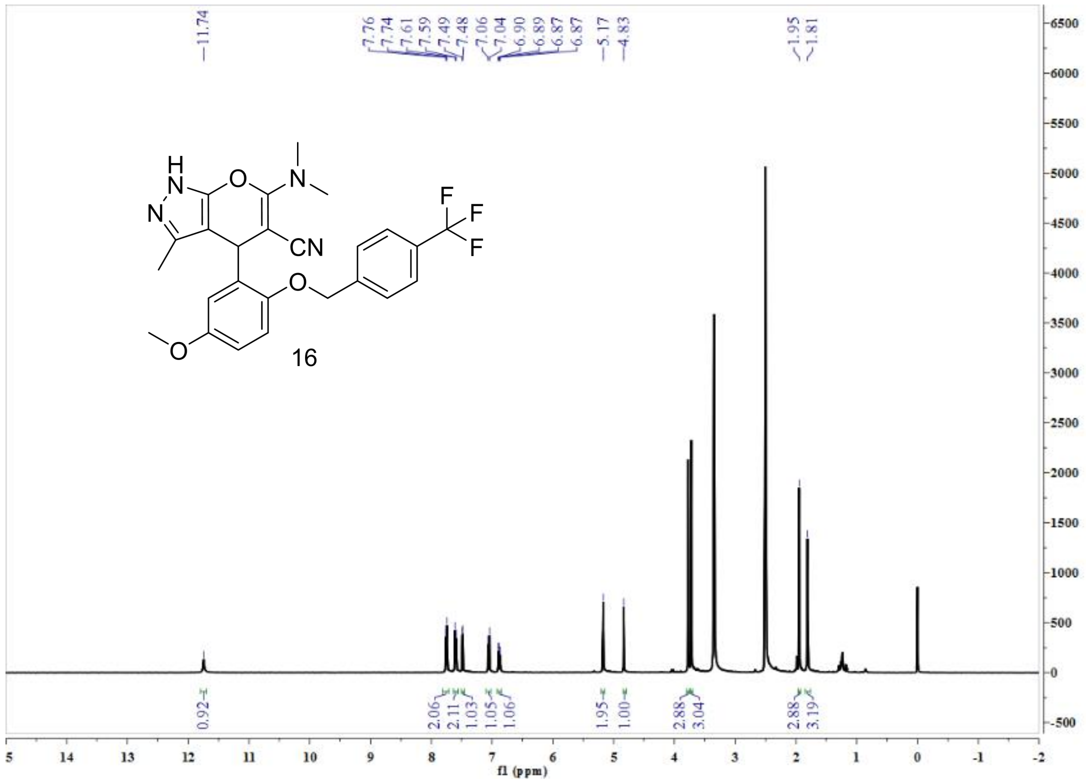
11.74
7.76
7.74
7.61
7.59
7.49
7.48
7.06
7.04
6.90
6.89
6.87
6.87
5.17
4.83
1.95
1.81
0.92
2.06
2.11
1.03
1.05
1.06
1.95
1.00
2.88
3.04
2.88
3.19
f1 (ppm)
CN
F
F
F
O
O
O
N
N
H
N

16

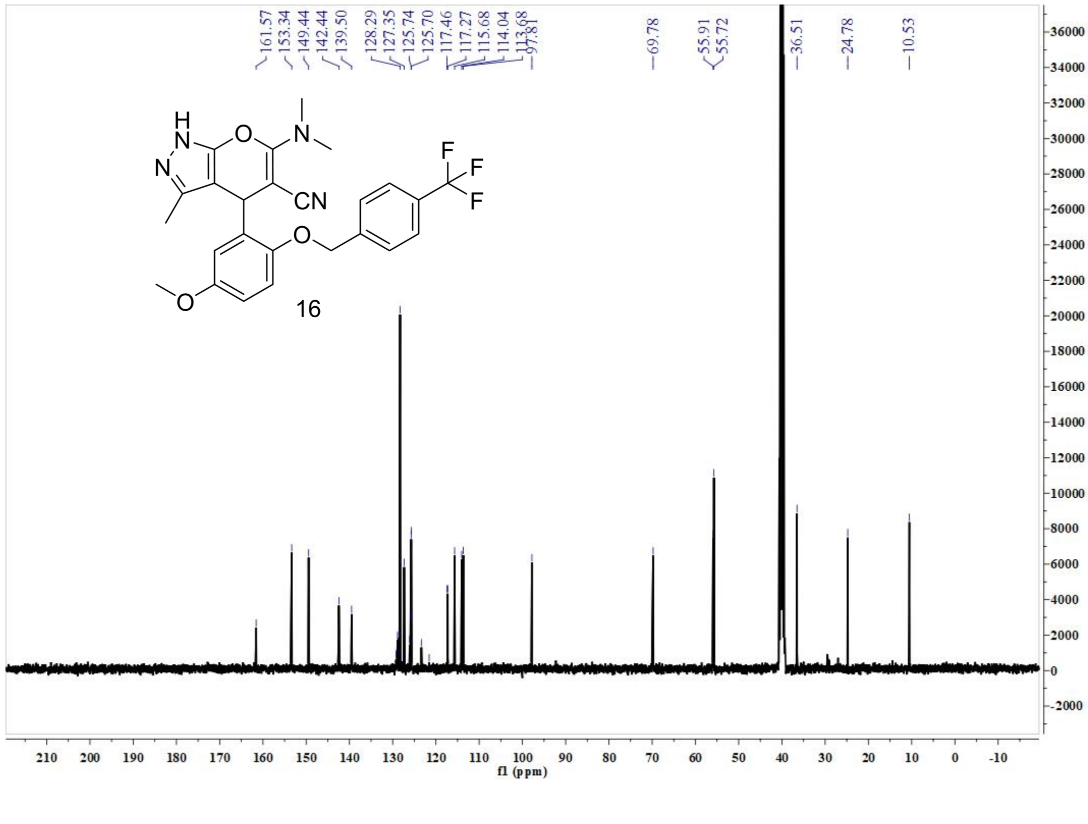
161.57
153.34
149.44
142.44
139.50
128.29
127.35
125.74
125.70
117.46
117.27
115.68
114.04
113.68
97.81
69.78
55.91
55.72
36.51
24.78
10.53
f1 (ppm)
CN
F
F
F
O
O
O
N
N
H
N

16

2. HRMS spectra of target compounds

HRMS spectrum of **4a**

Elmt	Val.	Min	Max	Elmt	Val.	Min	Max	Elmt	Val.	Min	Max	Use Adduct
H	1	0	21	Al	3	0	0	Zn	2	0	0	K
2H	1	0	0	Si	4	0	0	Se	2	0	0	H
B	3	0	0	P	3	0	0	Br	1	0	0	Na
C	4	0	28	S	2	0	0	Ru	2	0	0	NH4
N	3	0	4	Cl	1	0	1	Sb	3	0	0	
O	2	0	4	Fe	2	0	0	I	3	0	0	
F	1	0	3	Ni	2	0	0	Ir	3	0	0	
Na	1	0	0	Cu	2	0	0	Pt	2	0	0	

Error Margin (ppm): 100
HC Ratio: 0.0 - 100.0
Max Isotopes: all
MSn Iso RI (%): 75.00

DBE Range: -100.0 - 100.0
Apply N Rule: no
Isotope RI (%): 1.00
MSn Logic Mode: OR

Electron Ions: both
Use MSn Info: yes
Isotope Res: 10000
Max Results: 100

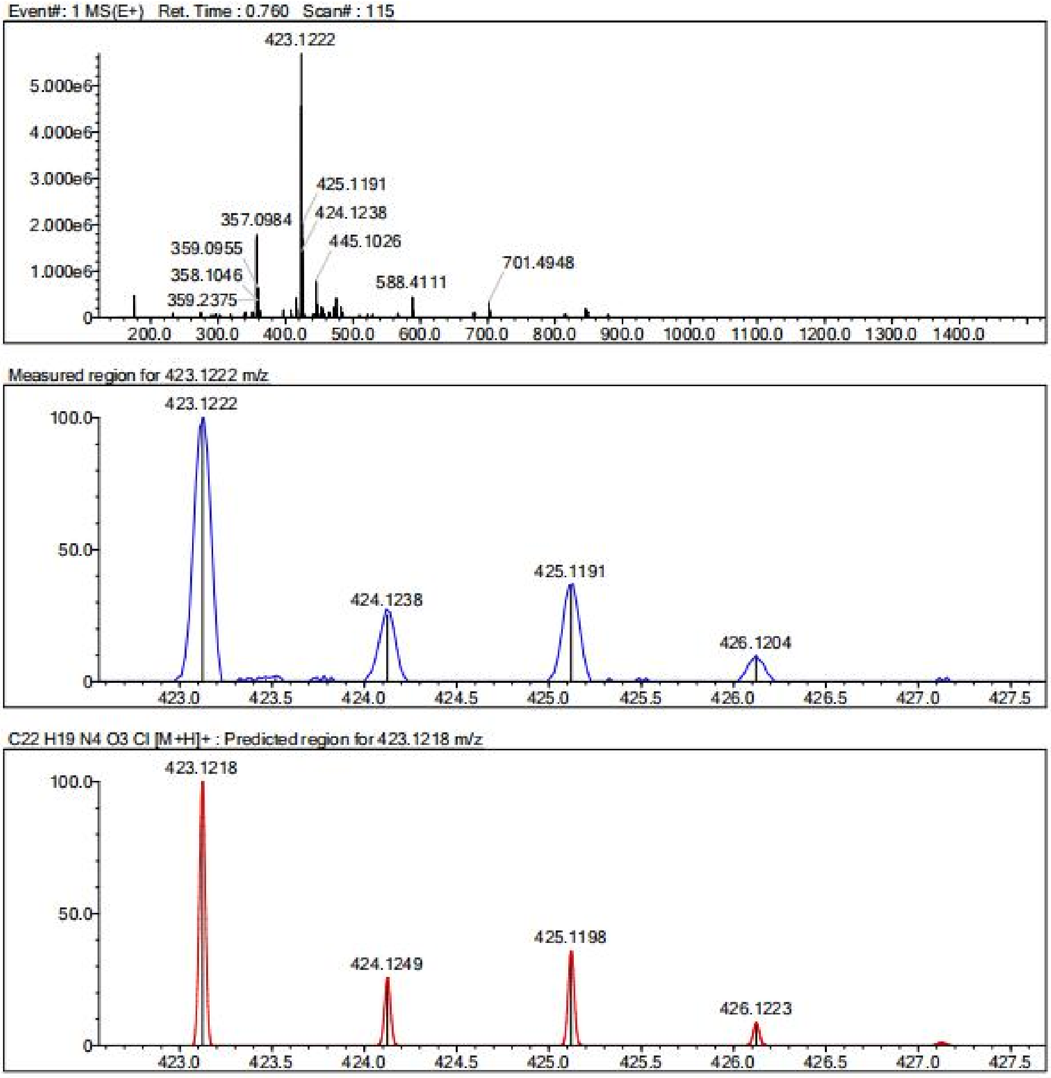

Rank	Score	Formula (M)	Ion	Meas. m/z	Pred. m/z	Df. (mDa)	Df. (ppm)	Iso	DBE
1	78.64	C22 H19 N4 O3 Cl	[M+H]+	423.1222	423.1218	0.4	0.95	78.64	15.0

HRMS spectrum of **4b**

Elmt	Val.	Min	Max	Elmt	Val.	Min	Max	Elmt	Val.	Min	Max	Use Adduct
H	1	0	21	Al	3	0	0	Zn	2	0	0	K
2H	1	0	0	Si	4	0	0	Se	2	0	0	H
B	3	0	0	P	3	0	0	Br	1	0	0	Na
C	4	0	28	S	2	0	0	Ru	2	0	0	NH4
N	3	0	4	Cl	1	0	1	Sb	3	0	0	
O	2	0	4	Fe	2	0	0	I	3	0	0	
F	1	0	3	Ni	2	0	0	Ir	3	0	0	
Na	1	0	0	Cu	2	0	0	Pt	2	0	0	

Error Margin (ppm):	100	DBE Range:	-100.0 - 100.0	Electron Ions:	both
HC Ratio:	0.0 - 100.0	Apply N Rule:	no	Use MSn Info:	yes
Max Isotopes:	all	Isotope RI (%):	1.00	Isotope Res:	10000
MSn Iso RI (%):	75.00	MSn Logic Mode:	OR	Max Results:	100

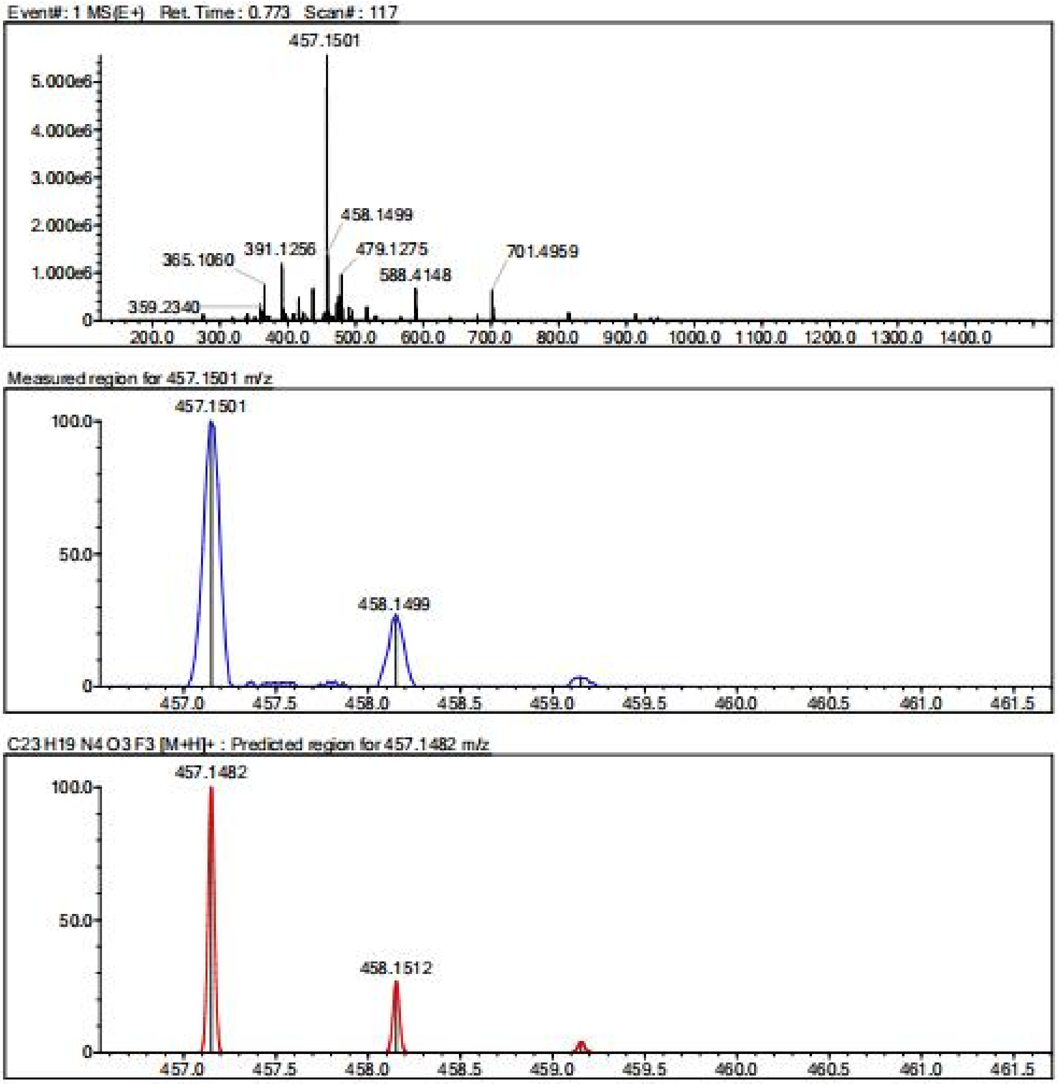

Rank	Score	Formula (M)	Ion	Meas. m/z	Pred. m/z	Df. (mDa)	Df. (ppm)	Iso	DBE
5	77.49	C23 H19 N4 O3 F3	[M+H]+	457.1501	457.1482	1.9	4.16	84.14	15.0

HRMS spectrum of **4c**

Elmt	Val.	Min	Max	Elmt	Val.	Min	Max	Elmt	Val.	Min	Max	Use Adduct
H	1	0	21	Al	3	0	0	Zn	2	0	0	K
2H	1	0	0	Si	4	0	0	Se	2	0	0	H
B	3	0	0	P	3	0	0	Br	1	0	0	Na
C	4	0	28	S	2	0	0	Ru	2	0	0	NH4
N	3	0	4	Cl	1	0	1	Sb	3	0	0	
O	2	0	4	Fe	2	0	0	I	3	0	0	
F	1	0	3	Ni	2	0	0	Ir	3	0	0	
Na	1	0	0	Cu	2	0	0	Pt	2	0	0	

Error Margin (ppm): 100
HC Ratio: 0.0 - 100.0
Max Isotopes: all
MSn Iso RI (%): 75.00

DBE Range: -100.0 - 100.0
Apply N Rule: no
Isotope RI (%): 1.00
MSn Logic Mode: OR

Electron Ions: both
Use MSn Info: yes
Isotope Res: 10000
Max Results: 100

Event#: 1 MS(E+) Ret. Time : 0.827 Scan# : 125

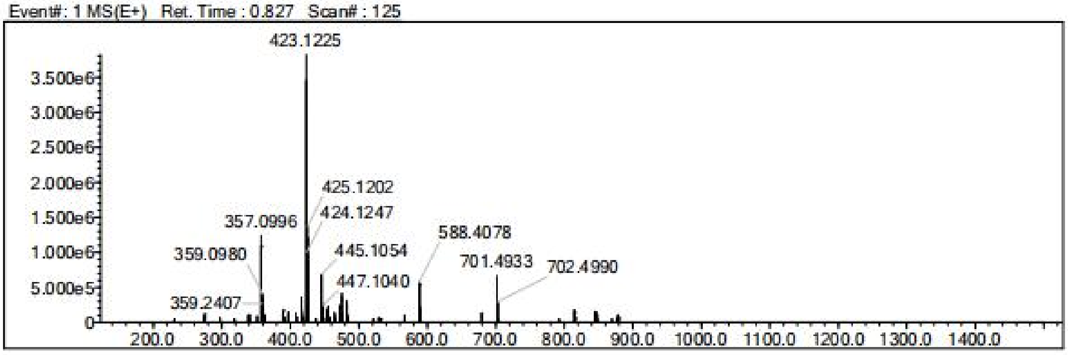

Measured region for 423.1225 m/z

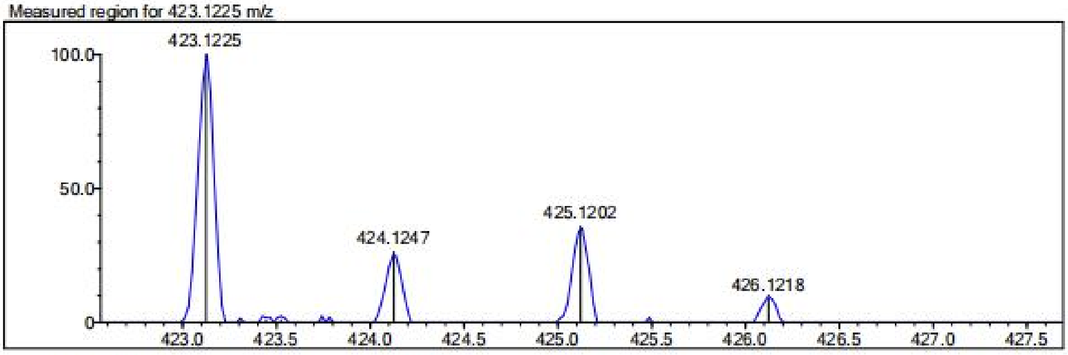

C22 H19 N4 O3 Cl [M+H]+ : Predicted region for 423.1218 m/z

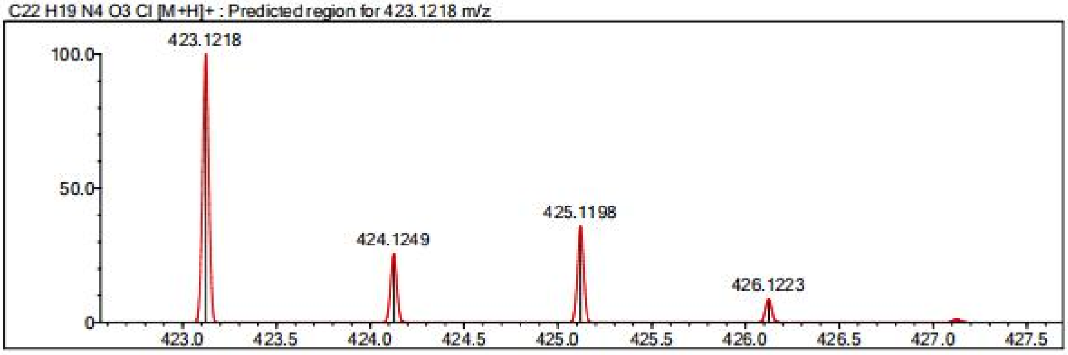

Rank	Score	Formula (M)	Ion	Meas. m/z	Pred. m/z	Df. (mDa)	Df. (ppm)	Iso	DBE
1	93.26	C22 H19 N4 O3 Cl	[M+H]+	423.1225	423.1218	0.7	1.65	94.80	15.0

HRMS spectrum of **8a**

Elmt	Val.	Min	Max	Elmt	Val.	Min	Max	Elmt	Val.	Min	Max	Use Adduct
H	1	0	17	Al	3	0	0	Zn	2	0	0	K
2H	1	0	0	Si	4	0	0	Se	2	0	0	H
B	3	0	0	P	3	0	0	Br	1	0	0	Na
C	4	0	23	S	2	0	0	Ru	2	0	0	NH4
N	3	0	4	Cl	1	0	0	Sb	3	0	0	
O	2	0	5	Fe	2	0	0	I	3	0	0	
F	1	0	3	Ni	2	0	0	Ir	3	0	0	
Na	1	0	0	Cu	2	0	0	Pt	2	0	0	

Error Margin (ppm): 100
HC Ratio: 0.0 - 100.0
Max Isotopes: all
MSn Iso RI (%): 75.00

DBE Range: -100.0 - 100.0
Apply N Rule: no
Isotope RI (%): 1.00
MSn Logic Mode: OR

Electron Ions: both
Use MSn Info: yes
Isotope Res: 10000
Max Results: 100

Event#: 1 MS(E+) Ret. Time : 0.773 Scan# : 117

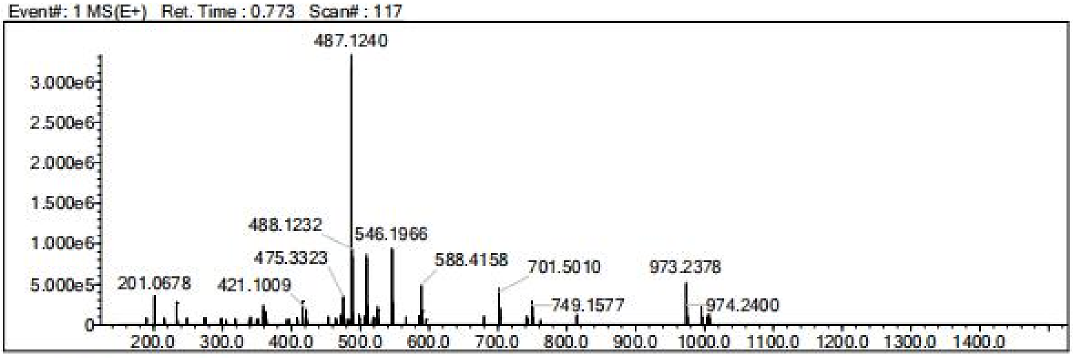

Measured region for 487.1240 m/z

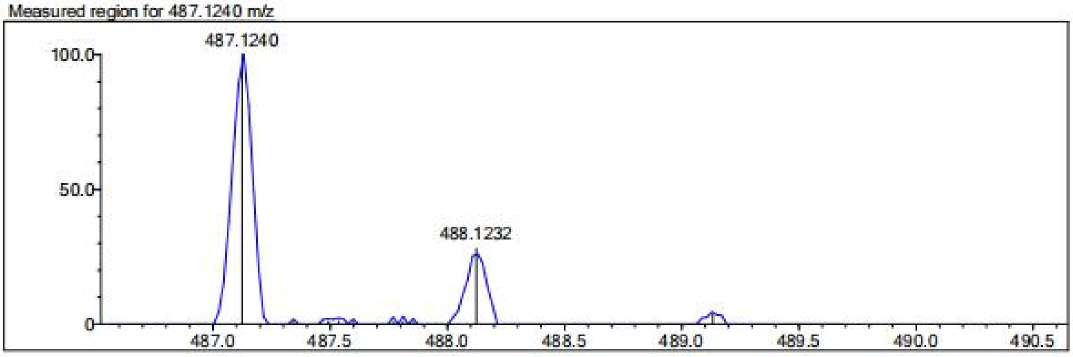

C23 H17 N4 O5 F3 [M+H]+ : Predicted region for 487.1224 m/z

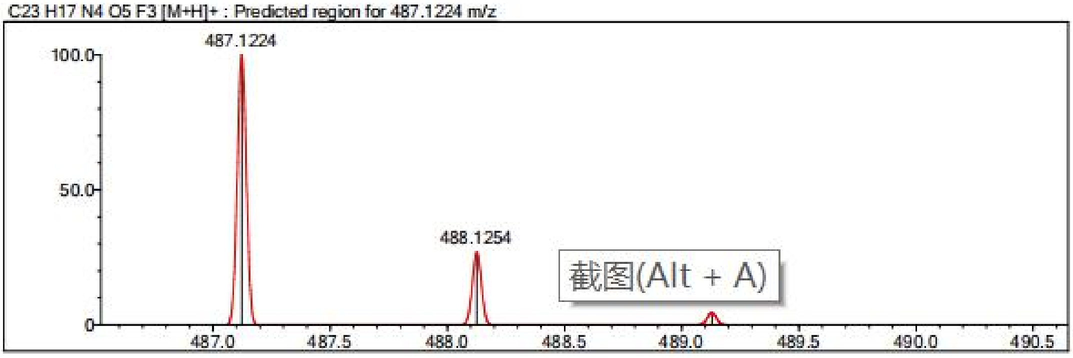

Rank	Score	Formula (M)	Ion	Meas. m/z	Pred. m/z	Df. (mDa)	Df. (ppm)	Iso	DBE
1	82.22	C23 H17 N4 O5 F3	[M+H]+	487.1240	487.1224	1.6	3.28	87.19	16.0

HRMS spectrum of **8b**

Elmt	Val.	Min	Max	Elmt	Val.	Min	Max	Elmt	Val.	Min	Max	Use Adduct
H	1	0	25	Al	3	0	0	Zn	2	0	0	K
2H	1	0	0	Si	4	0	0	Se	2	0	0	H
B	3	0	0	P	3	0	0	Br	1	0	0	Na
C	4	0	30	S	2	0	0	Ru	2	0	0	NH4
N	3	0	4	Cl	1	0	1	Sb	3	0	0	
O	2	0	4	Fe	2	0	0	I	3	0	0	
F	1	0	6	Ni	2	0	0	Ir	3	0	0	
Na	1	0	0	Cu	2	0	0	Pt	2	0	0	

Error Margin (ppm): 100
HC Ratio: 0.0 - 100.0
Max Isotopes: all
MSn Iso RI (%): 75.00

DBE Range: -100.0 - 100.0
Apply N Rule: no
Isotope RI (%): 1.00
MSn Logic Mode: OR

Electron Ions: both
Use MSn Info: yes
Isotope Res: 10000
Max Results: 100

Event#: 1 MS(E+) Ret. Time : 0.760 Scan# : 115

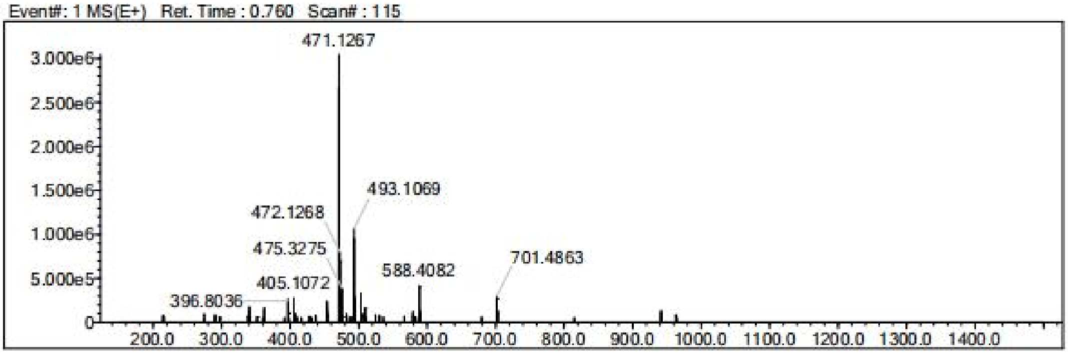

Measured region for 471.1267 m/z

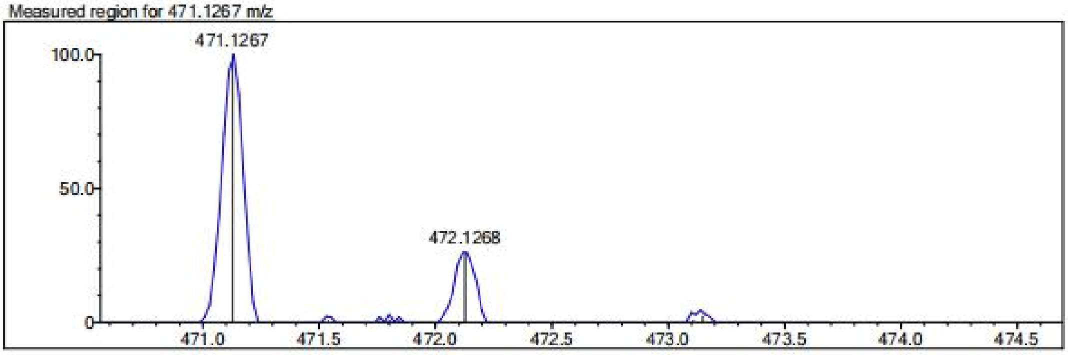

C23 H17 N4 O4 F3 [M+H]+ : Predicted region for 471.1275 m/z

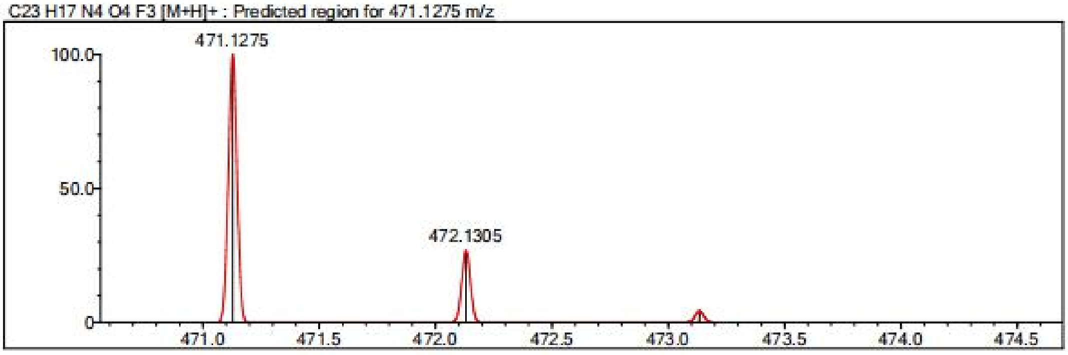

Rank	Score	Formula (M)	Ion	Meas. m/z	Pred. m/z	Df. (mDa)	Df. (ppm)	Iso	DBE
5	87.87	C23 H17 N4 O4 F3	[M+H]+	471.1267	471.1275	-0.8	-1.70	89.44	16.0

HRMS spectrum of **8c**

Elmt	Val.	Min	Max	Elmt	Val.	Min	Max	Elmt	Val.	Min	Max	Use Adduct
H	1	0	25	Al	3	0	0	Zn	2	0	0	K
2H	1	0	0	Si	4	0	0	Se	2	0	0	H
B	3	0	0	P	3	0	0	Br	1	0	0	Na
C	4	0	30	S	2	0	0	Ru	2	0	0	NH4
N	3	0	4	Cl	1	0	1	Sb	3	0	0	
O	2	0	4	Fe	2	0	0	I	3	0	0	
F	1	0	6	Ni	2	0	0	Ir	3	0	0	
Na	1	0	0	Cu	2	0	0	Pt	2	0	0	

Error Margin (ppm): 100
HC Ratio: 0.0 - 100.0
Max Isotopes: all
MSn Iso RI (%): 75.00

DBE Range: -100.0 - 100.0
Apply N Rule: no
Isotope RI (%): 1.00
MSn Logic Mode: OR

Electron Ions: both
Use MSn Info: yes
Isotope Res: 10000
Max Results: 100

Event#: 1 MS(E+) Ret. Time : 0.827 Scan# : 125

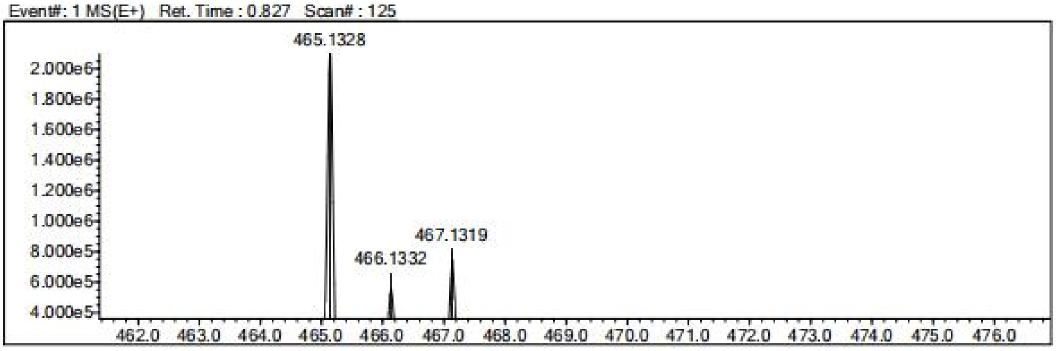

Measured region for 465.1328 m/z

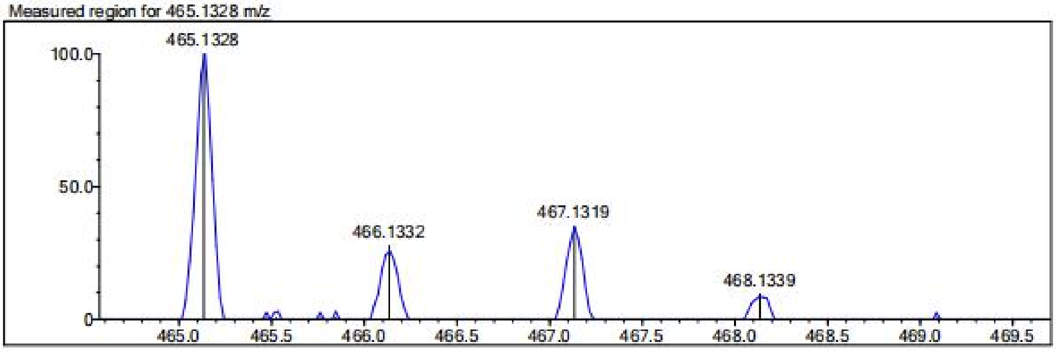

C24 H21 N4 O4 Cl [M+H]+ : Predicted region for 465.1324 m/z

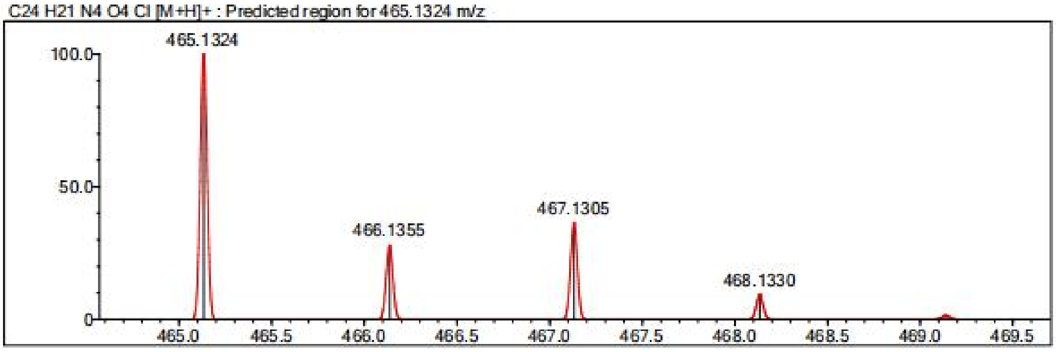

Rank	Score	Formula (M)	Ion	Meas. m/z	Pred. m/z	Df. (mDa)	Df. (ppm)	Iso	DBE
6	89.13	C24 H21 N4 O4 Cl	[M+H]+	465.1328	465.1324	0.4	0.86	89.13	16.0

HRMS spectrum of **8d**

Elmt	Val.	Min	Max	Elmt	Val.	Min	Max	Elmt	Val.	Min	Max	Use Adduct
H	1	0	25	Al	3	0	0	Zn	2	0	0	K
2H	1	0	0	Si	4	0	0	Se	2	0	0	H
B	3	0	0	P	3	0	0	Br	1	0	0	Na
C	4	0	30	S	2	0	0	Ru	2	0	0	NH4
N	3	0	4	Cl	1	0	1	Sb	3	0	0	
O	2	0	4	Fe	2	0	0	I	3	0	0	
F	1	0	6	Ni	2	0	0	Ir	3	0	0	
Na	1	0	0	Cu	2	0	0	Pt	2	0	0	

Error Margin (ppm): 100
HC Ratio: 0.0 - 100.0
Max Isotopes: all
MSn Iso RI (%): 75.00

DBE Range: -100.0 - 100.0
Apply N Rule: no
Isotope RI (%): 1.00
MSn Logic Mode: OR

Electron Ions: both
Use MSn Info: yes
Isotope Res: 10000
Max Results: 100

Event#: 1 MS(E+) Ret. Time : 0.880 Scan# : 133

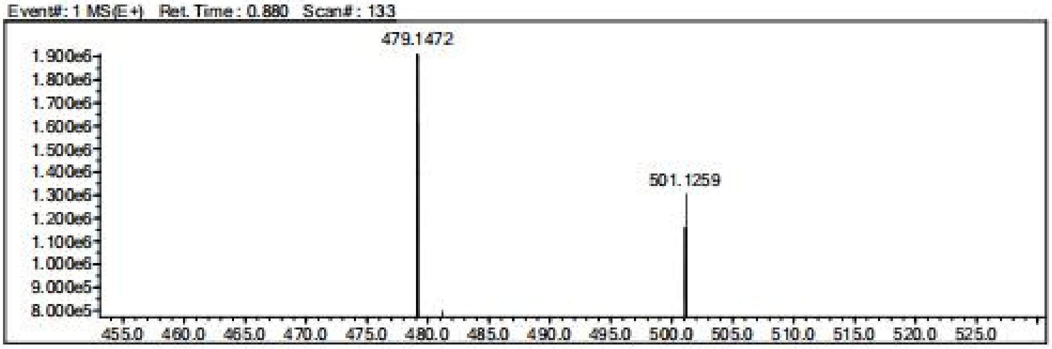

Measured region for 479.1472 m/z

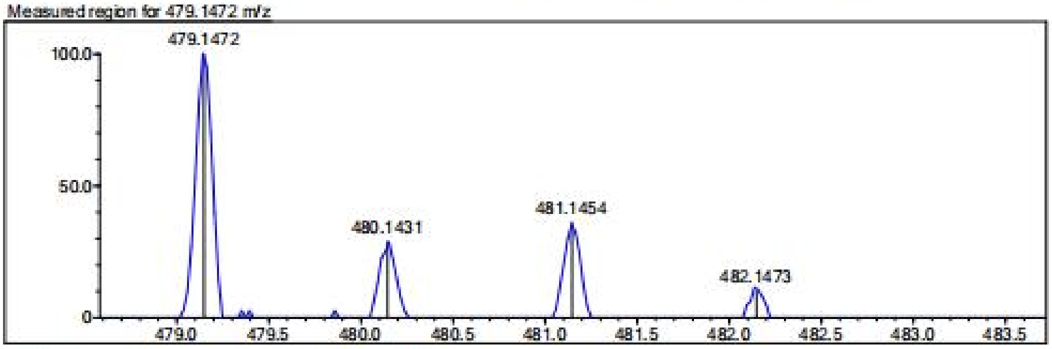

C25 H23 N4 O4 Cl [M+H]+ : Predicted region for 479.1481 m/z

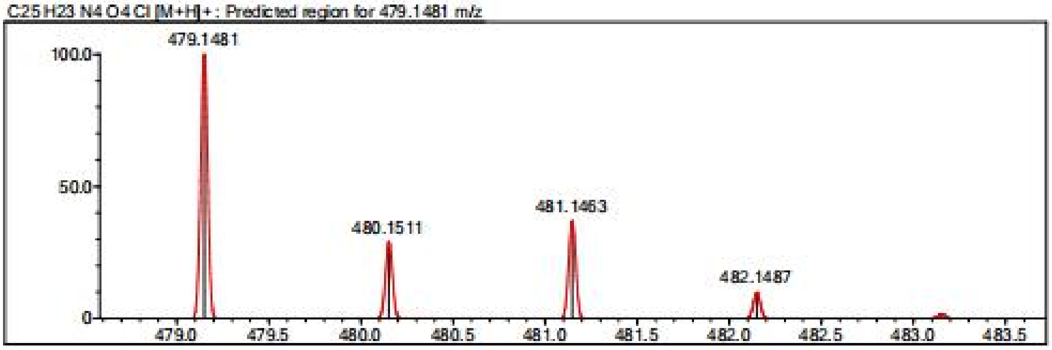

Rank	Score	Formula (M)	Ion	Meas. m/z	Pred. m/z	Df. (mDa)	Df. (ppm)	Iso	DBE
5	95.78	C25 H23 N4 O4 Cl	[M+H]+	479.1472	479.1481	-0.9	-1.88	97.94	16.0

HRMS spectrum of **11a**

Elmt	Val.	Min	Max	Elmt	Val.	Min	Max	Elmt	Val.	Min	Max	Elmt	Val.	Min	Max	Use Adduct
H	1	0	25	O	2	0	4	S	2	0	0	Br	1	0	0	H
2H	1	0	0	18O	2	0	0	Cl	1	0	0	Pd	2	0	0	
B	3	0	0	F	1	0	0	K	1	0	0	I	3	0	0	
C	4	0	25	Si	4	0	0	Mn	3	0	0	Pt	2	0	0	
N	3	0	4	P	3	0	0	Se	2	0	0					

Error Margin (ppm): 100
HC Ratio: 0.0 - 10.0
Max Isotopes: all
MSn Iso RI (%): 75.00

DBE Range: not fixed
Apply N Rule: no
Isotope RI (%): 1.00
MSn Logic Mode: AND

Electron Ions: both
Use MSn Info: no
Isotope Res: 10000
Max Results: 100

Event#: 1 MS(E+) Ret. Time : 0.853 Scan# : 129

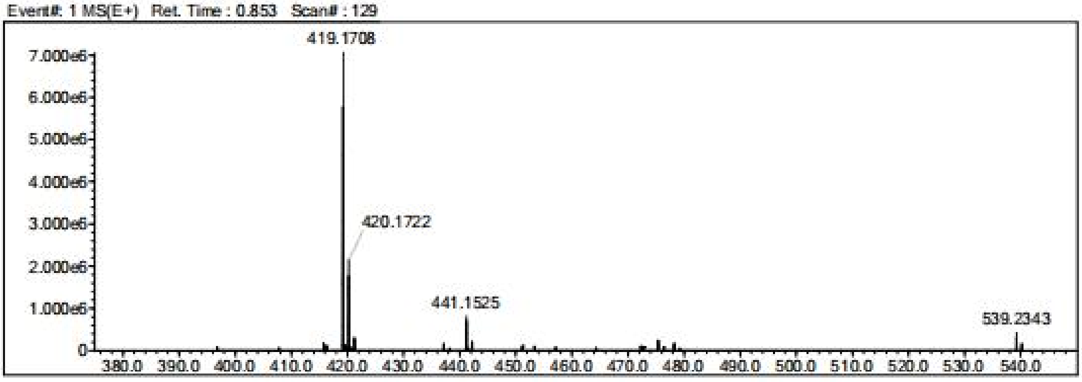

Measured region for 419.1708 m/z

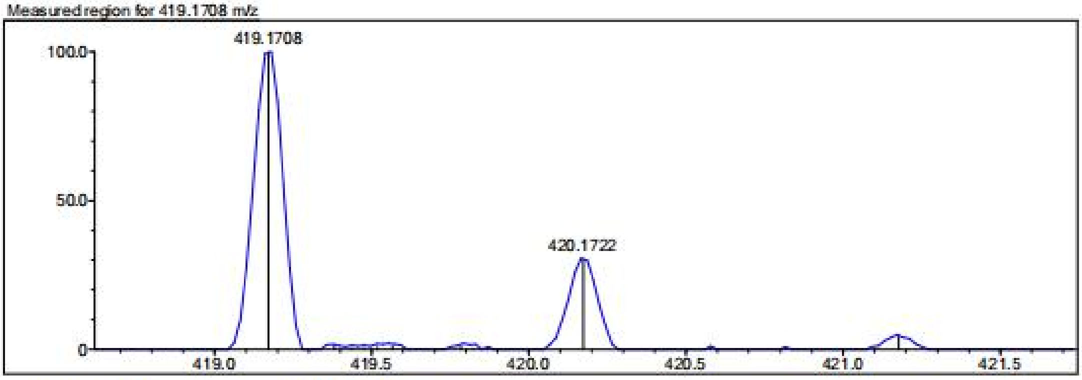

C23 H22 N4 O4 [M+H]+ : Predicted region for 419.1714 m/z

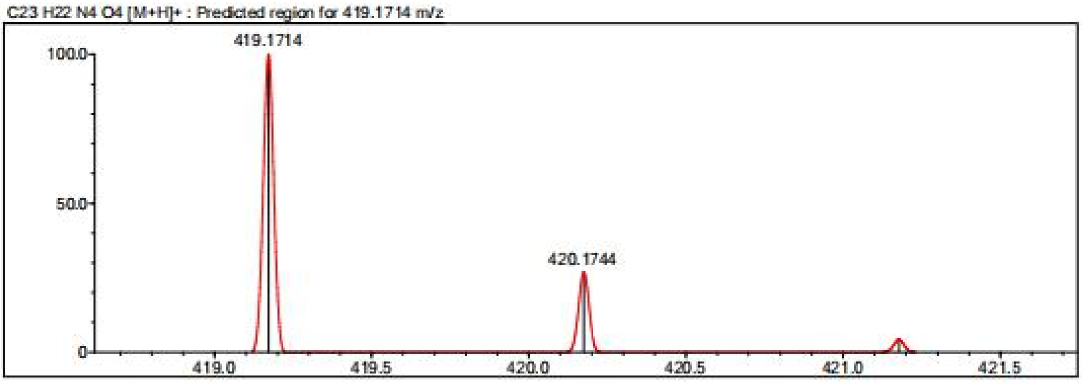

Rank	Score	Formula (M)	Ion	Meas. m/z	Pred. m/z	Df. (mDa)	Df. (ppm)	Iso	DBE
1	72.95	C23 H22 N4 O4	[M+H]+	419.1708	419.1714	-0.6	-1.43	73.74	15.0

HRMS spectrum of **11b**

Elmt	Val.	Min	Max	Elmt	Val.	Min	Max	Elmt	Val.	Min	Max	Use Adduct
H	1	0	23	Al	3	0	0	Zn	2	0	0	K
2H	1	0	0	Si	4	0	0	Se	2	0	0	H
B	3	0	0	P	3	0	0	Br	1	0	0	Na
C	4	0	25	S	2	0	0	Ru	2	0	0	NH4
N	3	0	5	Cl	1	0	1	Sb	3	0	0	
O	2	0	5	Fe	2	0	0	I	3	0	0	
F	1	0	3	Ni	2	0	0	Ir	3	0	0	
Na	1	0	0	Cu	2	0	0	Pt	2	0	0	

Error Margin (ppm): 100
HC Ratio: 0.0 - 100.0
Max Isotopes: all
MSn Iso RI (%): 75.00

DBE Range: -100.0 - 100.0
Apply N Rule: no
Isotope RI (%): 1.00
MSn Logic Mode: OR

Electron Ions: both
Use MSn Info: yes
Isotope Res: 10000
Max Results: 100

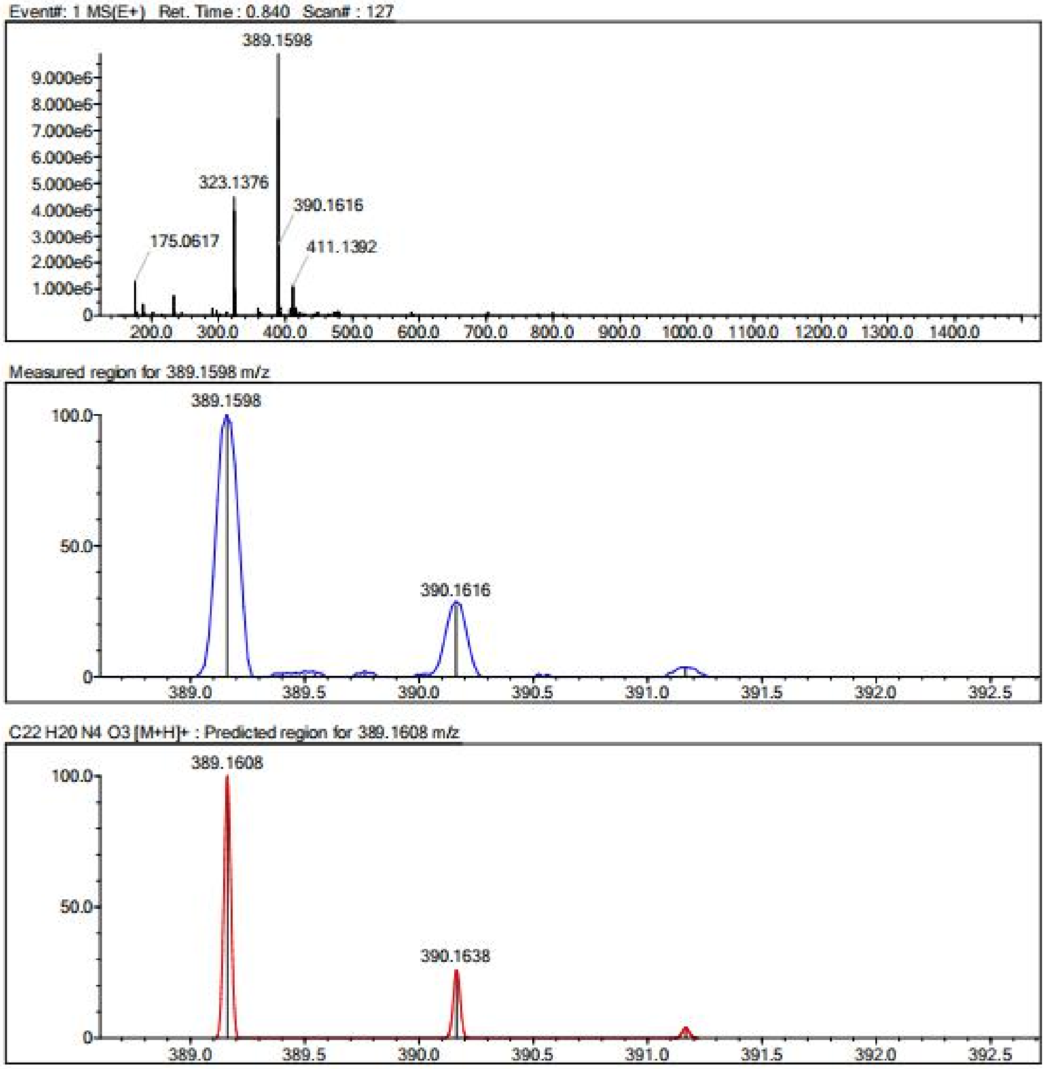

Rank	Score	Formula (M)	Ion	Meas. m/z	Pred. m/z	Df. (mDa)	Df. (ppm)	Iso	DBE
4	73.41	C22 H20 N4 O3	[M+H]+	389.1598	389.1608	-1.0	-2.57	76.41	15.0

HRMS spectrum of **11c**

Elmt	Val.	Min	Max	Elmt	Val.	Min	Max	Elmt	Val.	Min	Max	Elmt	Val.	Min	Max	Use Adduct
H	1	0	25	O	2	0	4	S	2	0	0	Br	1	0	0	H
2H	1	0	0	18O	2	0	0	Cl	1	0	1	Pd	2	0	0	
B	3	0	0	F	1	0	0	K	1	0	0	I	3	0	0	
C	4	0	25	Si	4	0	0	Mn	3	0	0	Pt	2	0	0	
N	3	0	4	P	3	0	0	Se	2	0	0					

Error Margin (ppm): 100
HC Ratio: 0.0 - 10.0
Max Isotopes: all
MSn Iso RI (%): 75.00

DBE Range: not fixed
Apply N Rule: no
Isotope RI (%): 1.00
MSn Logic Mode: AND

Electron Ions: both
Use MSn Info: no
Isotope Res: 10000
Max Results: 100

Event#: 1 MS(E+) Ret. Time : 0.760 Scan# : 115

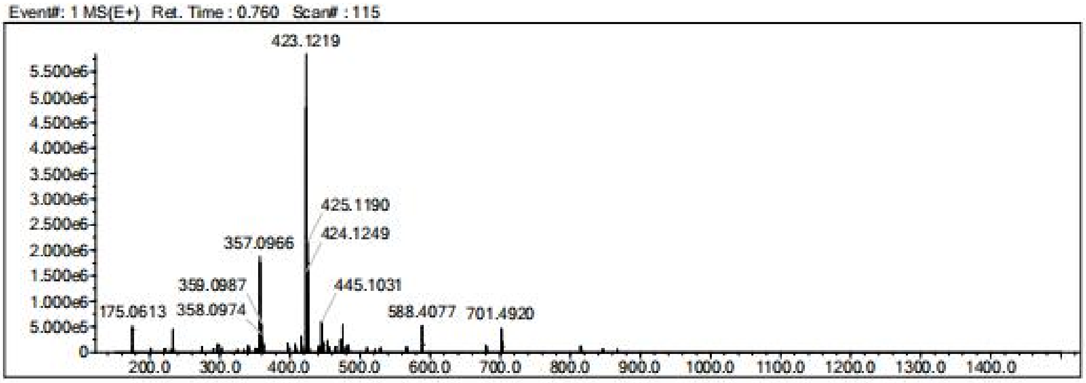

Measured region for 423.1219 m/z

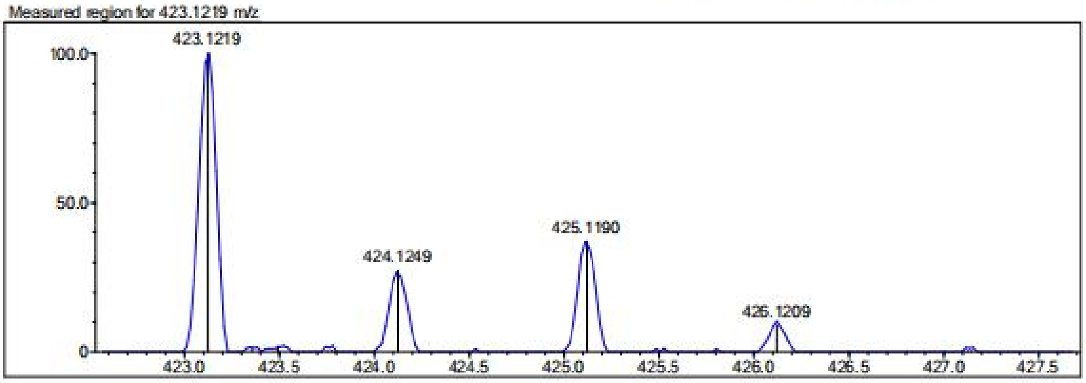

C22 H19 N4 O3 Cl [M+H]+ : Predicted region for 423.1218 m/z

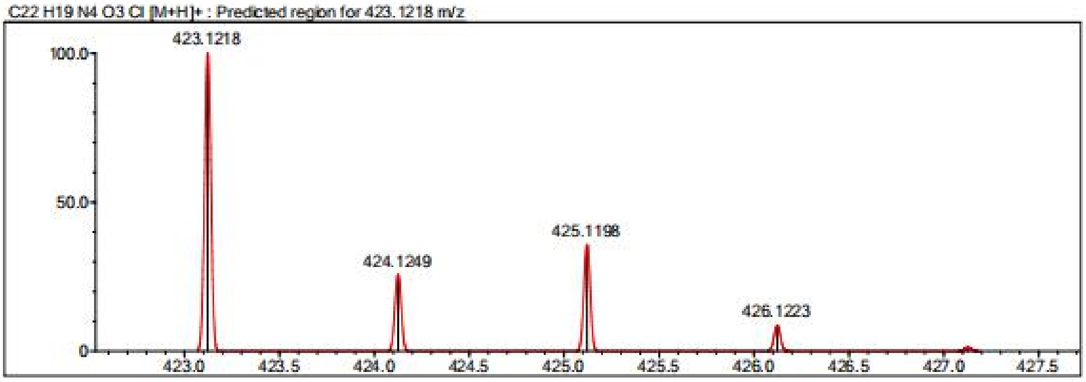

Rank	Score	Formula (M)	Ion	Meas. m/z	Pred. m/z	Df. (mDa)	Df. (ppm)	Iso	DBE
1	90.94	C22 H19 N4 O3 Cl	[M+H]+	423.1219	423.1218	0.1	0.24	90.94	15.0

HRMS spectrum of **11d**

Elmt	Val.	Min	Max	Elmt	Val.	Min	Max	Elmt	Val.	Min	Max	Use Adduct
H	1	0	23	Al	3	0	0	Zn	2	0	0	K
2H	1	0	0	Si	4	0	0	Se	2	0	0	H
B	3	0	0	P	3	0	0	Br	1	0	0	Na
C	4	0	25	S	2	0	0	Ru	2	0	0	NH4
N	3	0	5	Cl	1	0	1	Sb	3	0	0	
O	2	0	5	Fe	2	0	0	I	3	0	0	
F	1	0	3	Ni	2	0	0	Ir	3	0	0	
Na	1	0	0	Cu	2	0	0	Pt	2	0	0	

Error Margin (ppm):	100	DBE Range:	-100.0 - 100.0	Electron Ions:	both
HC Ratio:	0.0 - 100.0	Apply N Rule:	no	Use MSn Info:	yes
Max Isotopes:	all	Isotope RI (%):	1.00	Isotope Res:	10000
MSn Iso RI (%):	75.00	MSn Logic Mode:	OR	Max Results:	100

Event#: 1 MS(E+) Ret. Time : 0.760 Scan# : 115

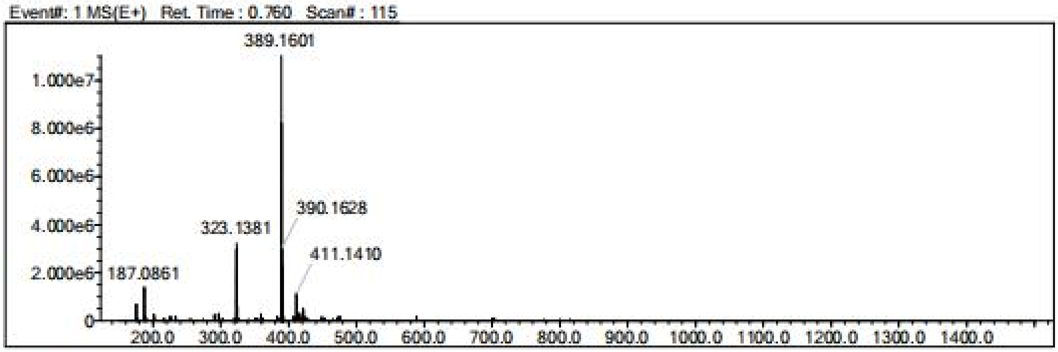

Measured region for 389.1601 m/z

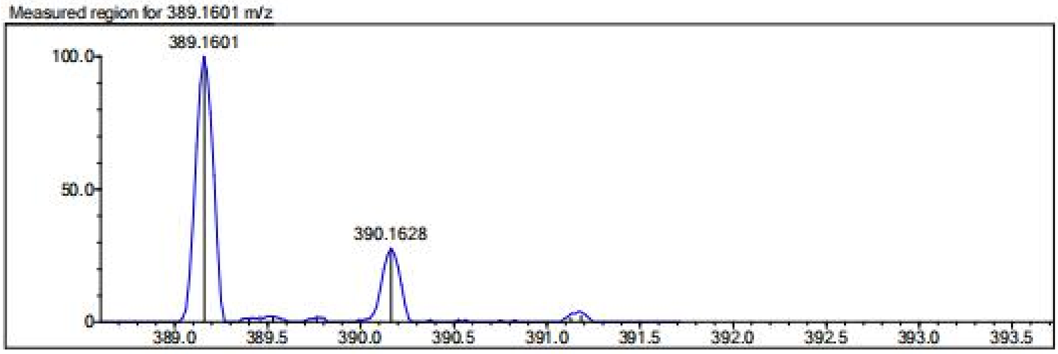

C22 H20 N4 O3 [M+H]+ : Predicted region for 389.1608 m/z

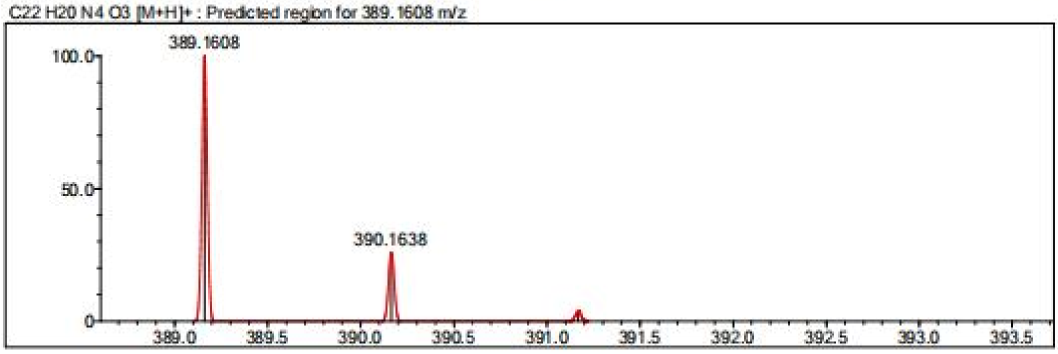

Rank	Score	Formula (M)	Ion	Meas. m/z	Pred. m/z	Df. (mDa)	Df. (ppm)	Iso	DBE
4	70.11	C22 H20 N4 O3	[M+H]+	389.1601	389.1608	-0.7	-1.80	71.54	15.0

HRMS spectrum of **11e**

Elmt	Val.	Min	Max	Elmt	Val.	Min	Max	Elmt	Val.	Min	Max	Elmt	Val.	Min	Max	Use Adduct
H	1	0	25	O	2	0	4	S	2	0	0	Br	1	0	0	H
2H	1	0	0	18O	2	0	0	Cl	1	0	1	Pd	2	0	0	
B	3	0	0	F	1	0	0	K	1	0	0	I	3	0	0	
C	4	0	25	Si	4	0	0	Mn	3	0	0	Pt	2	0	0	
N	3	0	4	P	3	0	0	Se	2	0	0					

Error Margin (ppm): 100
HC Ratio: 0.0 - 10.0
Max Isotopes: all
MSn Iso RI (%): 75.00
DBE Range: not fixed
Apply N Rule: no
Isotope RI (%): 1.00
MSn Logic Mode: AND
Electron Ions: both
Use MSn Info: no
Isotope Res: 10000
Max Results: 100

Event#: 1 MS(E+) Ret. Time : 0.773 Scan# : 117

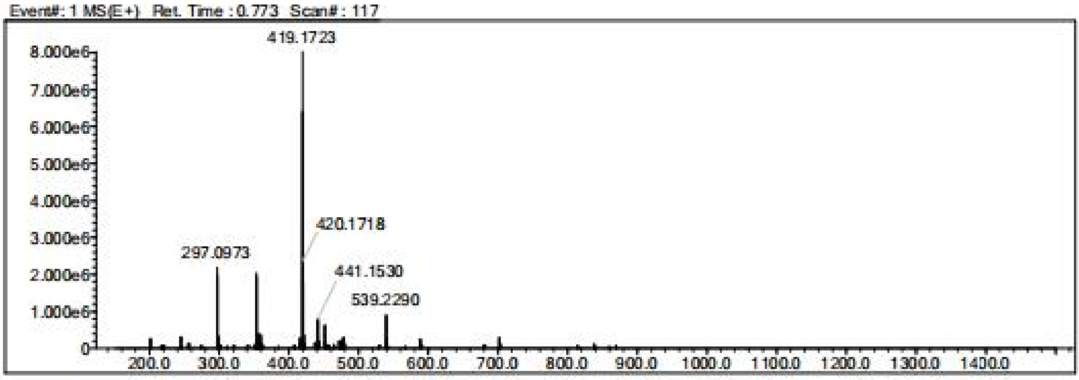

Measured region for 419.1723 m/z

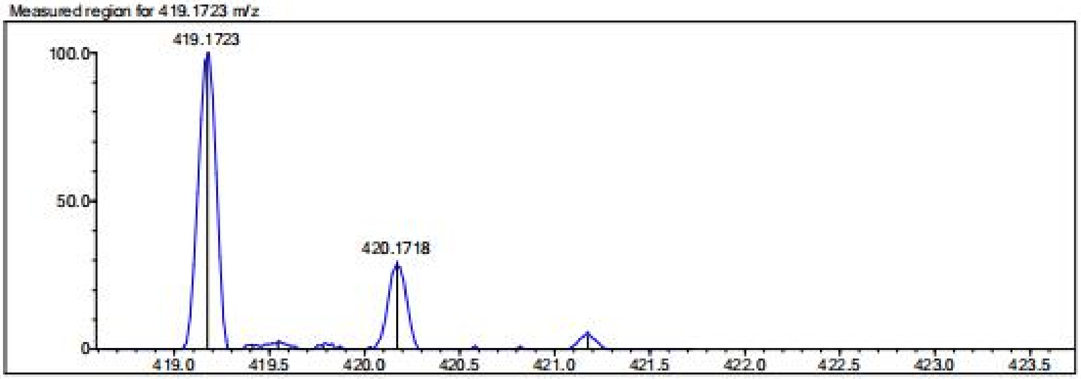

C23 H22 N4 O4 [M+H]+ : Predicted region for 419.1714 m/z

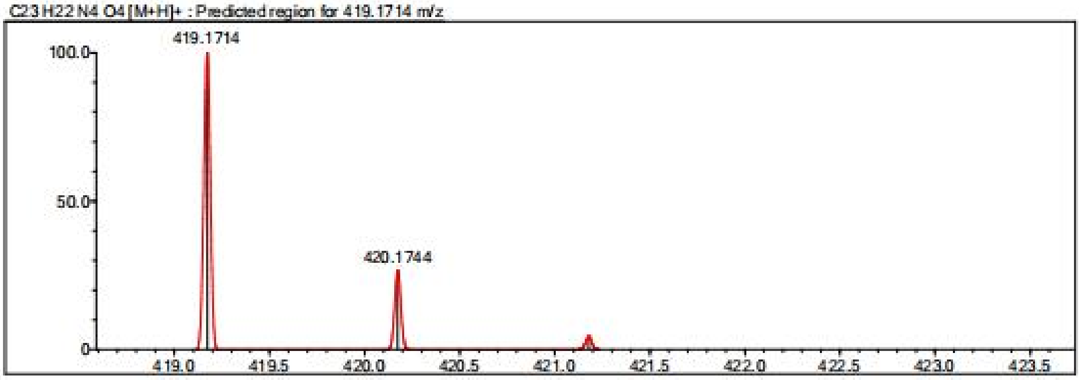

Rank	Score	Formula (M)	Ion	Meas. m/z	Pred. m/z	Df. (mDa)	Df. (ppm)	Iso	DBE
1	71.53	C23 H22 N4 O4	[M+H]+	419.1723	419.1714	0.9	2.15	73.65	15.0

HRMS spectrum of **11f**

Elmt	Val.	Min	Max	Elmt	Val.	Min	Max	Elmt	Val.	Min	Max	Use Adduct
H	1	0	23	Al	3	0	0	Zn	2	0	0	K
2H	1	0	0	Si	4	0	0	Se	2	0	0	H
B	3	0	0	P	3	0	0	Br	1	0	0	Na
C	4	0	25	S	2	0	0	Ru	2	0	0	NH4
N	3	0	5	Cl	1	0	1	Sb	3	0	0	
O	2	0	5	Fe	2	0	0	I	3	0	0	
F	1	0	3	Ni	2	0	0	Ir	3	0	0	
Na	1	0	0	Cu	2	0	0	Pt	2	0	0	

Error Margin (ppm): 100
HC Ratio: 0.0 - 100.0
Max Isotopes: all
MSn Iso RI (%): 75.00

DBE Range: -100.0 - 100.0
Apply N Rule: no
Isotope RI (%): 1.00
MSn Logic Mode: OR

Electron Ions: both
Use MSn Info: yes
Isotope Res: 10000
Max Results: 100

Event#: 1 MS(E+) Ret. Time : 0.773 Scan# : 117

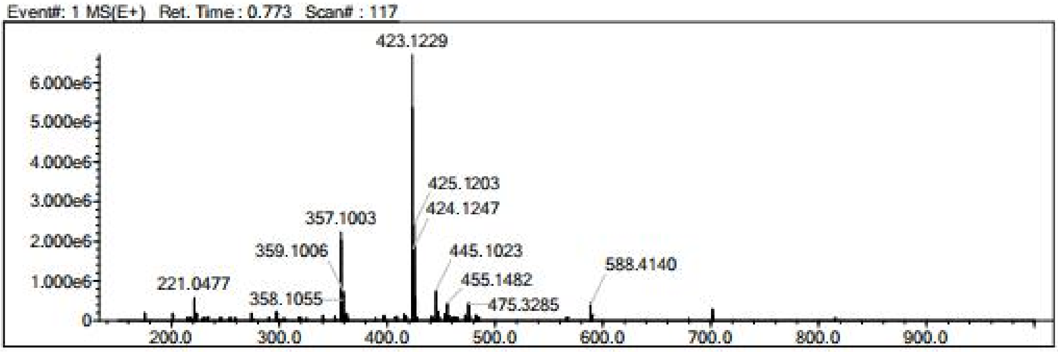

Measured region for 423.1229 m/z

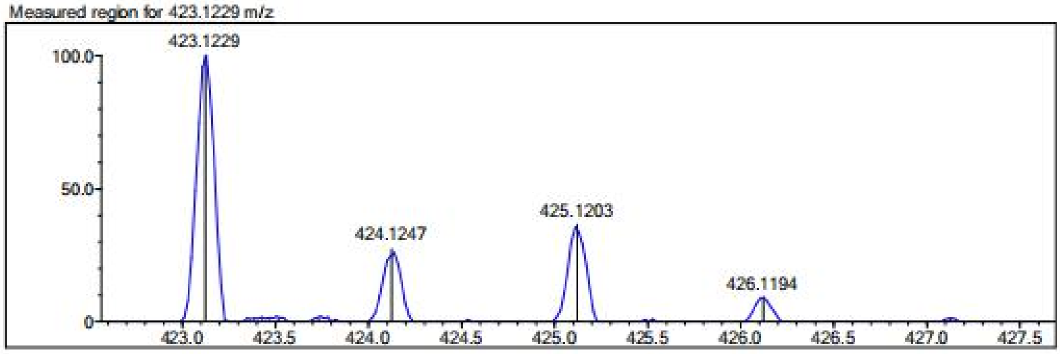

C22 H19 N4 O3 Cl [M+H]+ : Predicted region for 423.1218 m/z

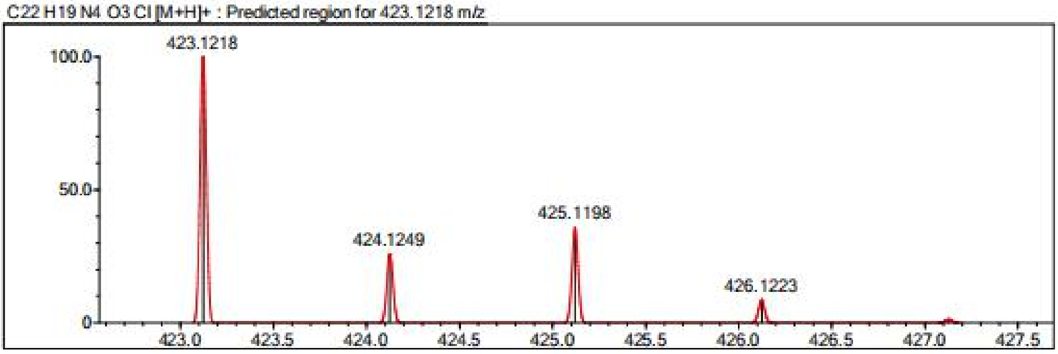

Rank	Score	Formula (M)	Ion	Meas. m/z	Pred. m/z	Df. (mDa)	Df. (ppm)	Iso	DBE
9	88.67	C22 H19 N4 O3 Cl	[M+H]+	423.1229	423.1218	1.1	2.60	92.36	15.0

HRMS spectrum of **11g**

Elmt	Val.	Min	Max	Elmt	Val.	Min	Max	Elmt	Val.	Min	Max	Use Adduct
H	1	0	23	Al	3	0	0	Zn	2	0	0	K
2H	1	0	0	Si	4	0	0	Se	2	0	0	H
B	3	0	0	P	3	0	0	Br	1	0	0	Na
C	4	0	25	S	2	0	0	Ru	2	0	0	NH4
N	3	0	5	Cl	1	0	1	Sb	3	0	0	
O	2	0	5	Fe	2	0	0	I	3	0	0	
F	1	0	3	Ni	2	0	0	Ir	3	0	0	
Na	1	0	0	Cu	2	0	0	Pt	2	0	0	

Error Margin (ppm): 100
HC Ratio: 0.0 - 100.0
Max Isotopes: all
MSn Iso RI (%): 75.00

DBE Range: -100.0 - 100.0
Apply N Rule: no
Isotope RI (%): 1.00
MSn Logic Mode: OR

Electron Ions: both
Use MSn Info: yes
Isotope Res: 10000
Max Results: 100

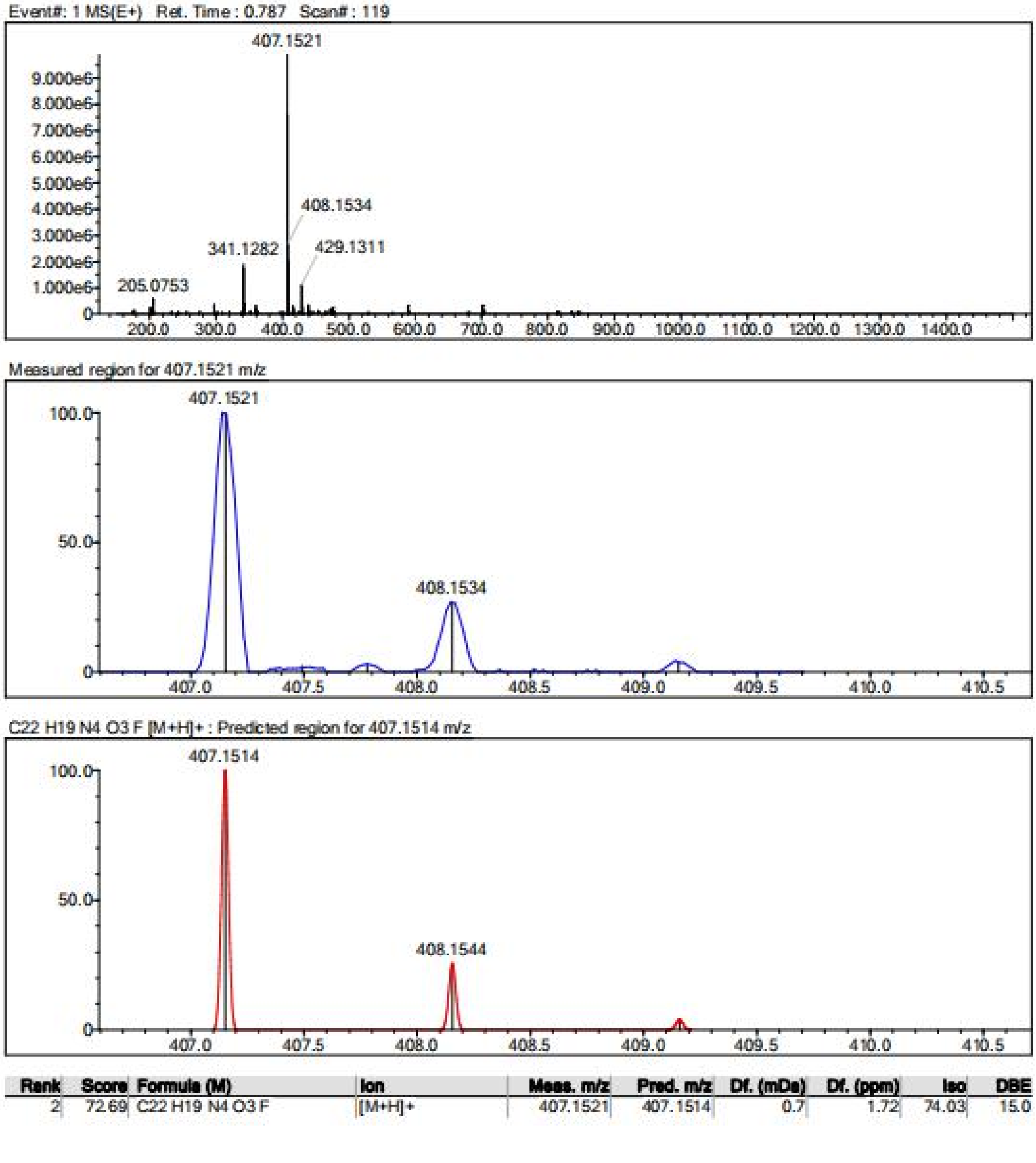

Rank	Score	Formula (M)	Ion	Meas. m/z	Pred. m/z	Df. (mDa)	Df. (ppm)	Iso	DBE
2	72.69	C22 H19 N4 O3 F	[M+H]+	407.1521	407.1514	0.7	1.72	74.03	15.0

HRMS spectrum of **11h**

Elmt	Val.	Min	Max	Elmt	Val.	Min	Max	Elmt	Val.	Min	Max	Use Adduct
H	1	0	25	Al	3	0	0	Zn	2	0	0	K
2H	1	0	0	Si	4	0	0	Se	2	0	0	H
B	3	0	0	P	3	0	0	Br	1	0	0	Na
C	4	0	25	S	2	0	0	Ru	2	0	0	NH4
N	3	0	4	Cl	1	0	0	Sb	3	0	0	
O	2	0	4	Fe	2	0	0	I	3	0	0	
F	1	0	3	Ni	2	0	0	Ir	3	0	0	
Na	1	0	0	Cu	2	0	0	Pt	2	0	0	

Error Margin (ppm): 100
HC Ratio: 0.0 - 100.0
Max Isotopes: all
MSn Iso RI (%): 75.00

DBE Range: -100.0 - 100.0
Apply N Rule: no
Isotope RI (%): 1.00
MSn Logic Mode: OR

Electron Ions: both
Use MSn Info: yes
Isotope Res: 10000
Max Results: 100

Event#: 1 MS(E+) Ret. Time : 0.853 Scan# : 129

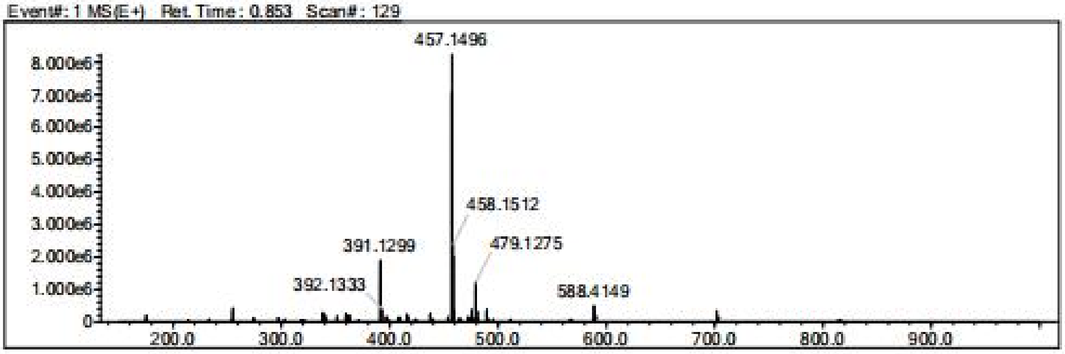

Measured region for 457.1496 m/z

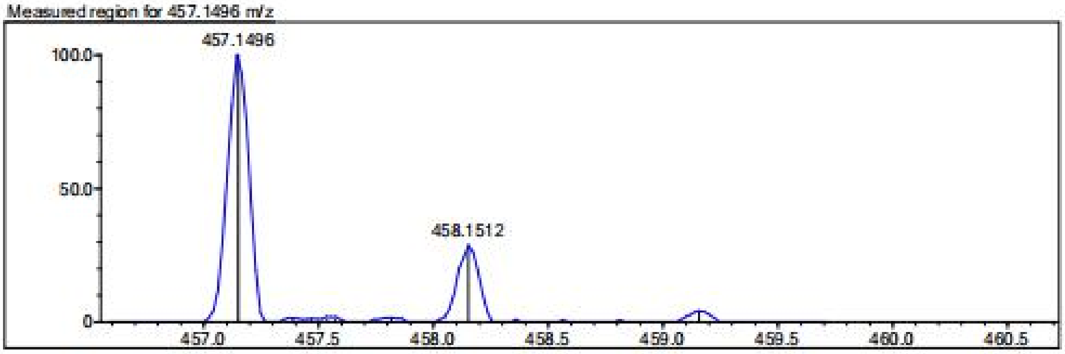

C23 H19 N4 O3 F3 [M+H]+ : Predicted region for 457.1482 m/z

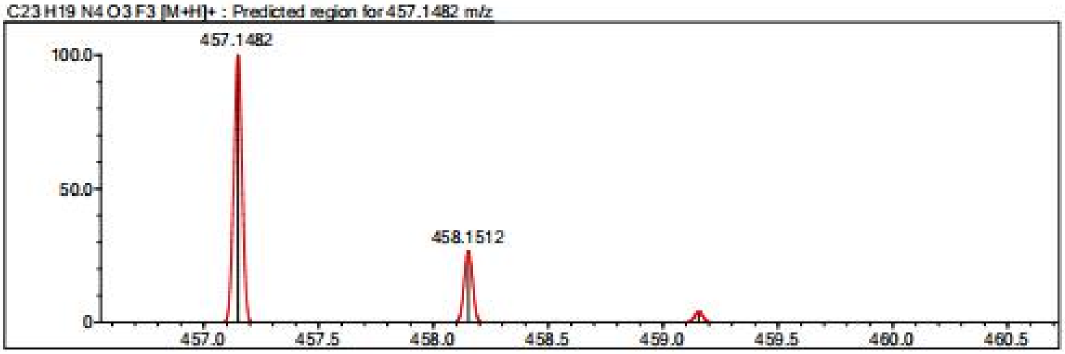

Rank	Score	Formula (M)	Ion	Meas. m/z	Pred. m/z	Df. (mDa)	Df. (ppm)	Iso	DBE
5	76.78	C23 H19 N4 O3 F3	[M+H]+	457.1496	457.1482	1.4	3.06	80.95	15.0

HRMS spectrum of **11i**

Elmt	Val.	Min	Max	Elmt	Val.	Min	Max	Elmt	Val.	Min	Max	Elmt	Val.	Min	Max	Use Adduct
H	1	0	19	O	2	0	4	S	2	0	0	Br	1	0	0	H
2H	1	0	0	18O	2	0	0	Cl	1	0	0	Pd	2	0	0	
B	3	0	0	F	1	0	3	K	1	0	0	I	3	0	0	
C	4	0	23	Si	4	0	0	Mn	3	0	0	Pt	2	0	0	
N	3	0	4	P	3	0	0	Se	2	0	0					

Error Margin (ppm): 100
HC Ratio: 0.0 - 10.0
Max Isotopes: all
MSn Iso RI (%): 75.00

DBE Range: not fixed
Apply N Rule: no
Isotope RI (%): 1.00
MSn Logic Mode: AND

Electron Ions: both
Use MSn Info: no
Isotope Res: 10000
Max Results: 100

Event#: 1 MS(E+) Ret. Time : 0.787 Scan# : 119

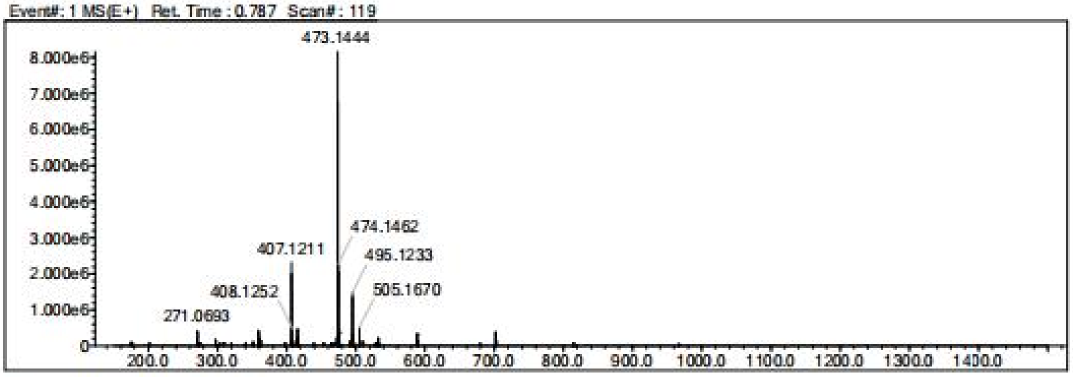

Measured region for 473.1444 m/z

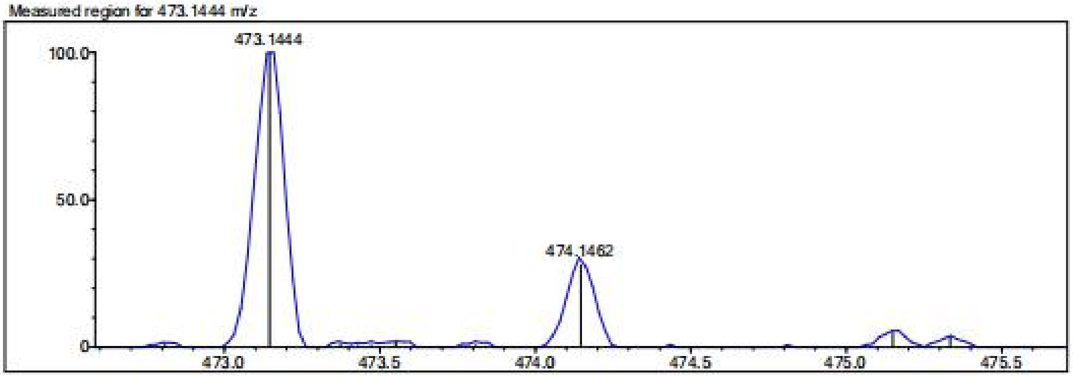

C23 H19 N4 O4 F3 [M+H]+ : Predicted region for 473.1431 m/z

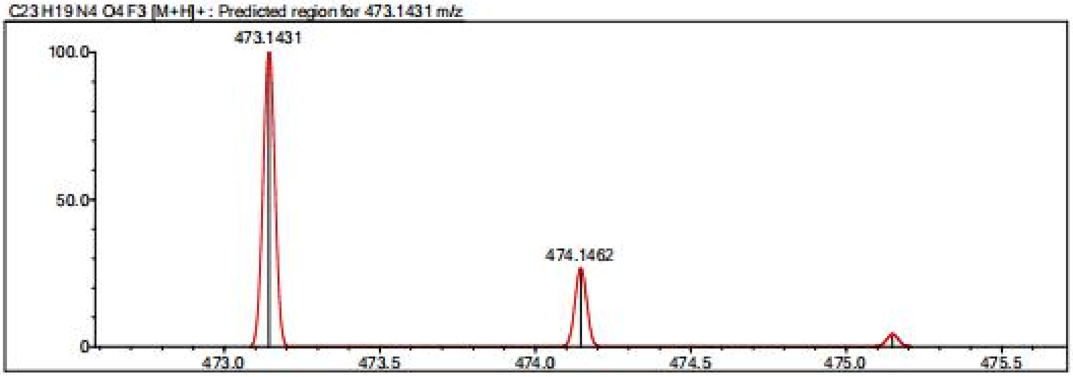

Rank	Score	Formula (M)	Ion	Meas. m/z	Pred. m/z	Df. (mDa)	Df. (ppm)	Iso	DBE
1	73.86	C23 H19 N4 O4 F3	[M+H]+	473.1444	473.1431	1.3	2.75	77.23	15.0

HRMS spectrum of **11j**

Elmt	Val.	Min	Max	Elmt	Val.	Min	Max	Elmt	Val.	Min	Max	Use Adduct
H	1	0	25	Al	3	0	0	Zn	2	0	0	K
2H	1	0	0	Si	4	0	0	Se	2	0	0	H
B	3	0	0	P	3	0	0	Br	1	0	0	Na
C	4	0	30	S	2	0	0	Ru	2	0	0	NH4
N	3	0	4	Cl	1	0	1	Sb	3	0	0	
O	2	0	4	Fe	2	0	0	I	3	0	0	
F	1	0	6	Ni	2	0	0	Ir	3	0	0	
Na	1	0	0	Cu	2	0	0	Pt	2	0	0	

Error Margin (ppm): 100
HC Ratio: 0.0 - 100.0
Max Isotopes: all
MSn Iso RI (%): 75.00

DBE Range: -100.0 - 100.0
Apply N Rule: no
Isotope RI (%): 1.00
MSn Logic Mode: OR

Electron Ions: both
Use MSn Info: yes
Isotope Res: 10000
Max Results: 100

Event#: 1 MS(E+) Ret. Time : 1.027 Scan# : 155

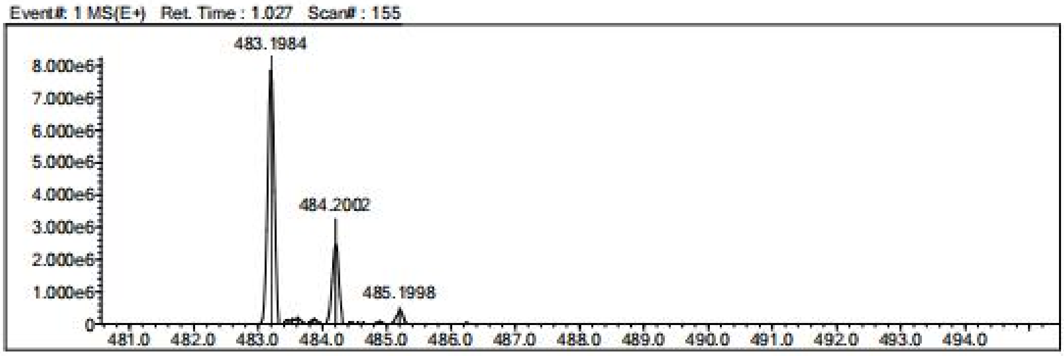

Measured region for 483.1984 m/z

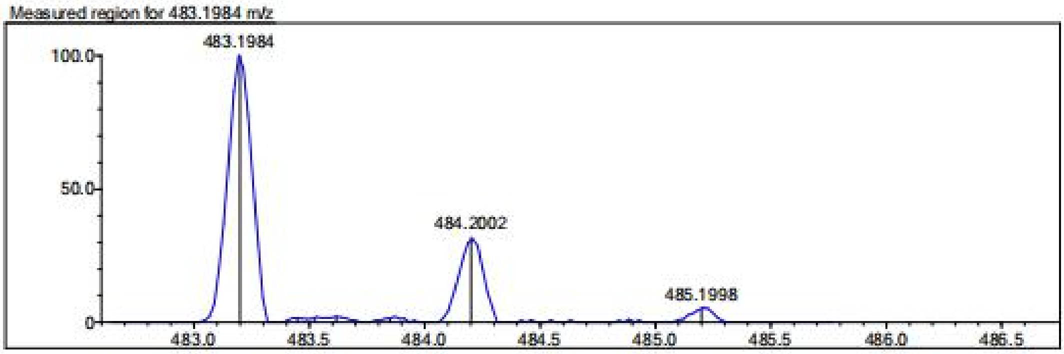

C26 H25 N4 O2 F3 [M+H]+ : Predicted region for 483.2002 m/z

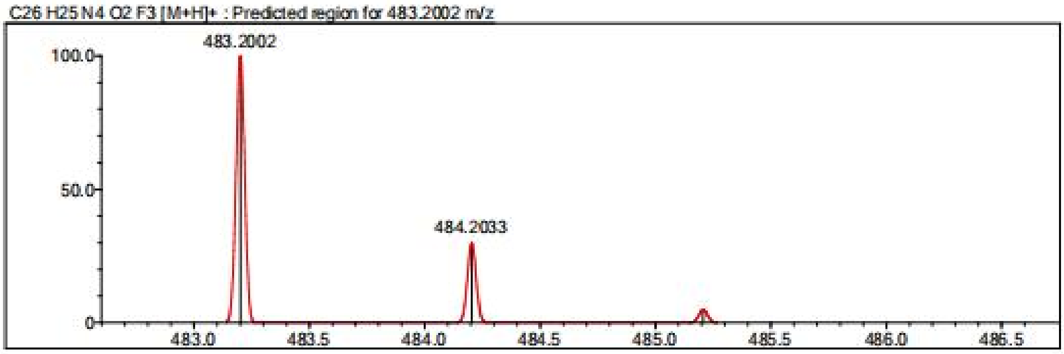

Rank	Score	Formula (M)	Ion	Meas. m/z	Pred. m/z	Df. (mDa)	Df. (ppm)	Iso	DBE
1	74.26	C26 H25 N4 O2 F3	[M+H]+	483.1984	483.2002	-1.8	-3.73	79.70	15.0

HRMS spectrum of **11k**

Elmt	Val.	Min	Max	Elmt	Val.	Min	Max	Elmt	Val.	Min	Max	Use Adduct
H	1	0	25	Al	3	0	0	Zn	2	0	0	K
2H	1	0	0	Si	4	0	0	Se	2	0	0	H
B	3	0	0	P	3	0	0	Br	1	0	0	Na
C	4	0	30	S	2	0	0	Ru	2	0	0	NH4
N	3	0	4	Cl	1	0	0	Sb	3	0	0	
O	2	0	4	Fe	2	0	0	I	3	0	0	
F	1	0	6	Ni	2	0	0	Ir	3	0	0	
Na	1	0	0	Cu	2	0	0	Pt	2	0	0	

Error Margin (ppm): 100
HC Ratio: 0.0 - 100.0
Max Isotopes: all
MSn Iso RI (%): 75.00

DBE Range: -100.0 - 100.0
Apply N Rule: no
Isotope RI (%): 1.00
MSn Logic Mode: OR

Electron Ions: both
Use MSn Info: yes
Isotope Res: 10000
Max Results: 100

Event#: 1 MS(E+) Ret. Time : 0.787 Scan# : 119

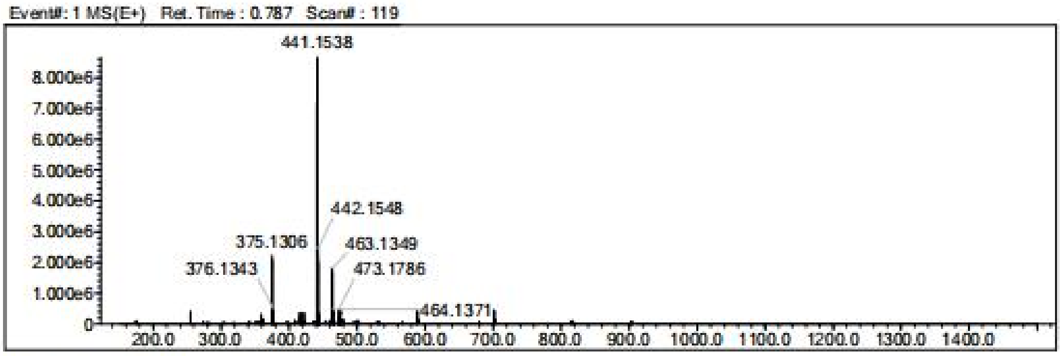

Measured region for 441.1538 m/z

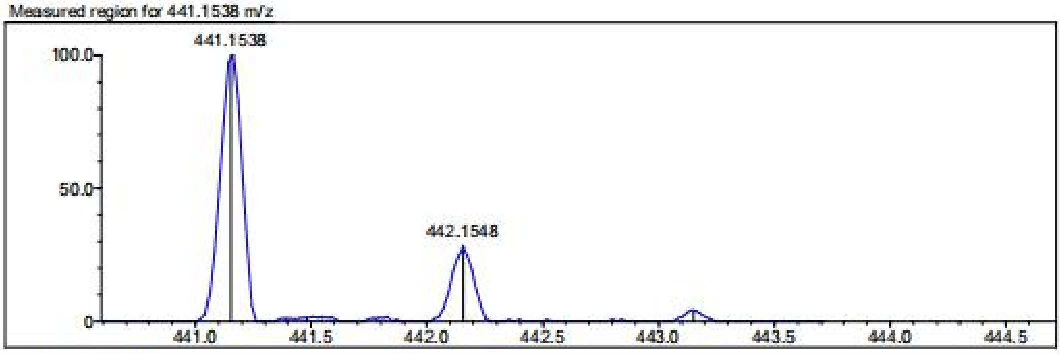

C23 H19 N4 O2 F3 [M+H]+ : Predicted region for 441.1533 m/z

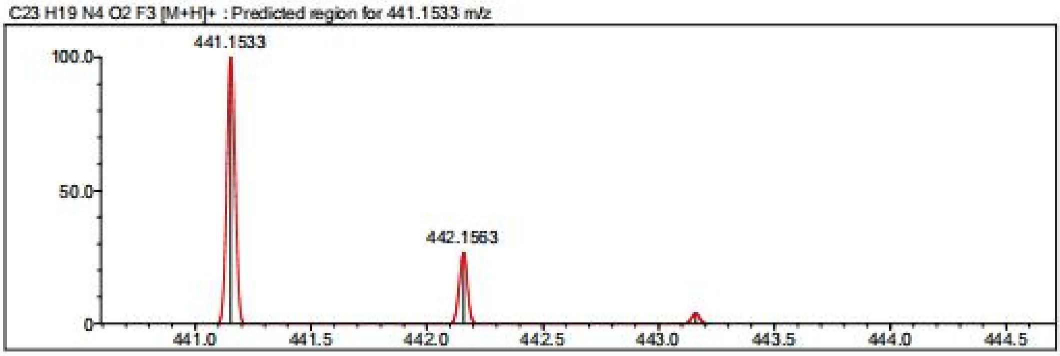

Rank	Score	Formula (M)	Ion	Meas. m/z	Pred. m/z	Df. (mDa)	Df. (ppm)	Iso	DBE
2	77.55	C23 H19 N4 O2 F3	[M+H]+	441.1538	441.1533	0.5	1.13	77.80	15.0

HRMS spectrum of **11l**

Elmt	Val.	Min	Max	Elmt	Val.	Min	Max	Elmt	Val.	Min	Max	Use Adduct
H	1	0	25	Al	3	0	0	Zn	2	0	0	K
2H	1	0	0	Si	4	0	0	Se	2	0	0	H
B	3	0	0	P	3	0	0	Br	1	0	0	Na
C	4	0	30	S	2	0	0	Ru	2	0	0	NH4
N	3	0	4	Cl	1	0	0	Sb	3	0	0	
O	2	0	4	Fe	2	0	0	I	3	0	0	
F	1	0	6	Ni	2	0	0	Ir	3	0	0	
Na	1	0	0	Cu	2	0	0	Pt	2	0	0	

Error Margin (ppm):	100	DBE Range:	-100.0 - 100.0	Electron Ions:	both
HC Ratio:	0.0 - 100.0	Apply N Rule:	no	Use MSn Info:	yes
Max Isotopes:	all	Isotope RI (%):	1.00	Isotope Res:	10000
MSn Iso RI (%):	75.00	MSn Logic Mode:	OR	Max Results:	100

Event#: 1 MS(E+) Ret. Time : 0.907 Scan# : 137

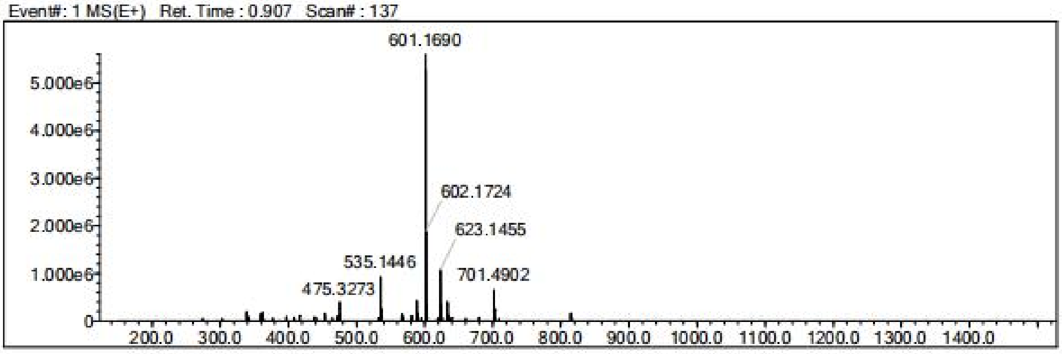

Measured region for 601.1690 m/z

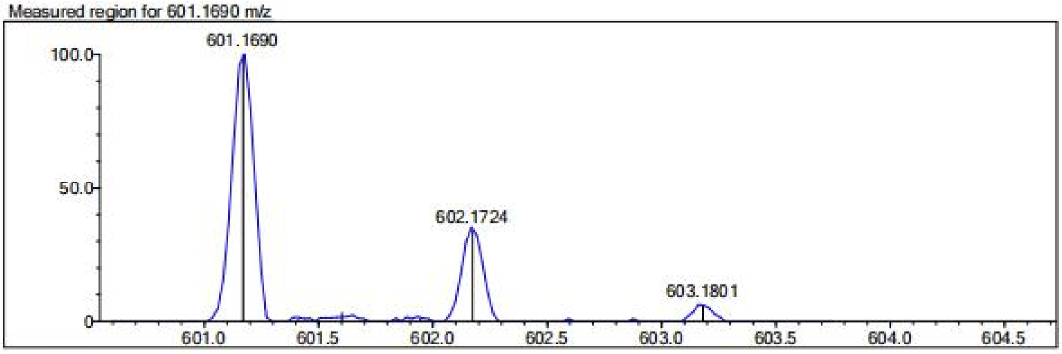

C30 H22 N4 O3 F6 [M+H]+ : Predicted region for 601.1669 m/z

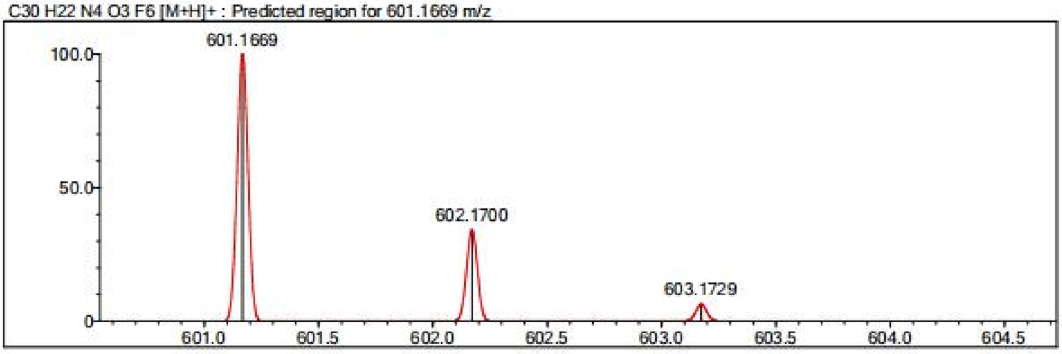

Rank	Score	Formula (M)	Ion	Meas. m/z	Pred. m/z	Df. (mDa)	Df. (ppm)	Iso	DBE
1	88.53	C30 H22 N4 O3 F6	[M+H]+	601.1690	601.1669	2.1	3.49	94.41	19.0

HRMS spectrum of **11m**

Elmt	Val.	Min	Max	Elmt	Val.	Min	Max	Elmt	Val.	Min	Max	Use Adduct
H	1	0	25	Al	3	0	0	Zn	2	0	0	K
2H	1	0	0	Si	4	0	0	Se	2	0	0	H
B	3	0	0	P	3	0	0	Br	1	0	0	Na
C	4	0	30	S	2	0	0	Ru	2	0	0	NH4
N	3	0	4	Cl	1	0	1	Sb	3	0	0	
O	2	0	4	Fe	2	0	0	I	3	0	0	
F	1	0	6	Ni	2	0	0	Ir	3	0	0	
Na	1	0	0	Cu	2	0	0	Pt	2	0	0	

Error Margin (ppm): 100
HC Ratio: 0.0 - 100.0
Max Isotopes: all
MSn Iso RI (%): 75.00

DBE Range: -100.0 - 100.0
Apply N Rule: no
Isotope RI (%): 1.00
MSn Logic Mode: OR

Electron Ions: both
Use MSn Info: yes
Isotope Res: 10000
Max Results: 100

Event#: 1 MS(E+) Ret. Time : 0.773 Scan# : 117

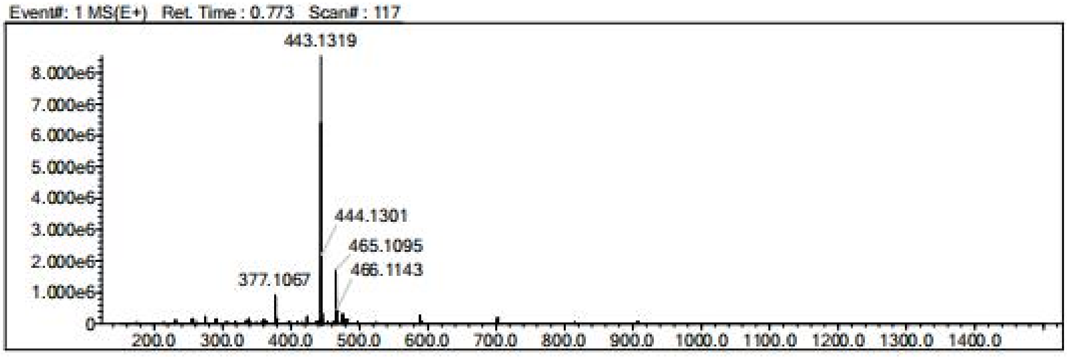

Measured region for 443.1319 m/z

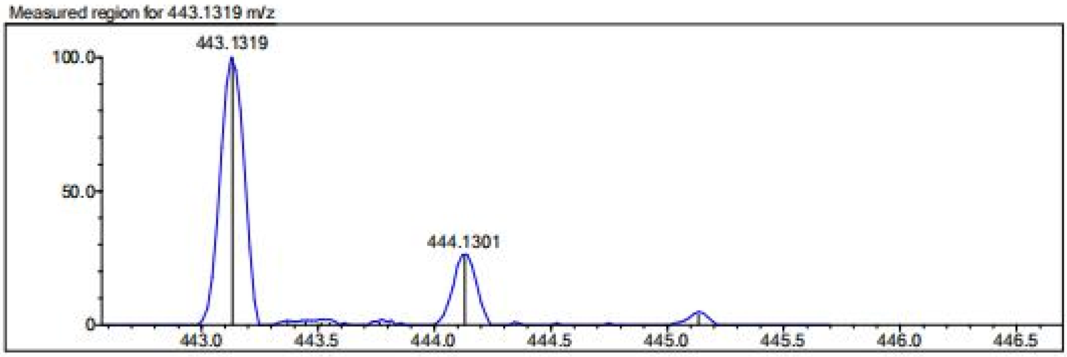

C22 H17 N4 O3 F3 [M+H]+ : Predicted region for 443.1326 m/z

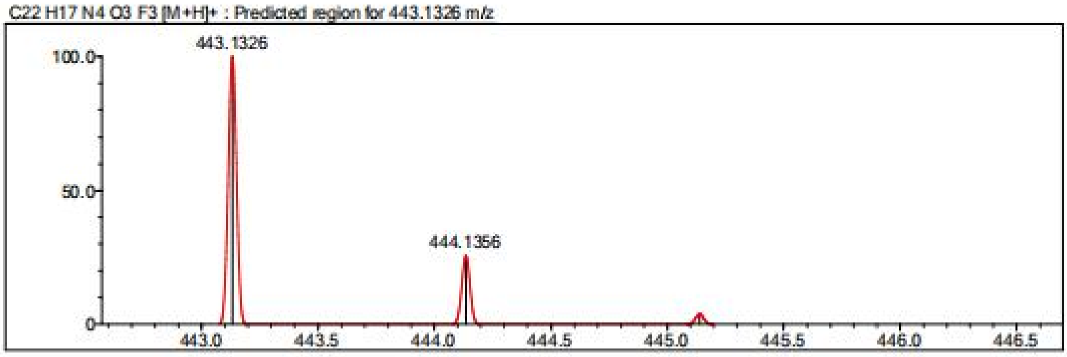

Rank	Score	Formula (M)	Ion	Meas. m/z	Pred. m/z	Df. (mDa)	Df. (ppm)	Iso	DBE
11	70.76	C22 H17 N4 O3 F3	[M+H]+	443.1319	443.1326	-0.7	-1.58	71.80	15.0

HRMS spectrum of **11n**

Elmt	Val.	Min	Max	Elmt	Val.	Min	Max	Elmt	Val.	Min	Max	Use Adduct
H	1	0	25	Al	3	0	0	Zn	2	0	0	K
2H	1	0	0	Si	4	0	0	Se	2	0	0	H
B	3	0	0	P	3	0	0	Br	1	0	0	Na
C	4	0	30	S	2	0	0	Ru	2	0	0	NH4
N	3	0	4	Cl	1	0	0	Sb	3	0	0	
O	2	0	4	Fe	2	0	0	I	3	0	0	
F	1	0	6	Ni	2	0	0	Ir	3	0	0	
Na	1	0	0	Cu	2	0	0	Pt	2	0	0	

Error Margin (ppm):	100	DBE Range:	-100.0 - 100.0	Electron Ions:	both
HC Ratio:	0.0 - 100.0	Apply N Rule:	no	Use MSn Info:	yes
Max Isotopes:	all	Isotope RI (%):	1.00	Isotope Res:	10000
MSn Iso RI (%):	75.00	MSn Logic Mode:	OR	Max Results:	100

Event#: 1 MS(E+) Ret. Time : 0.800 Scan# : 121

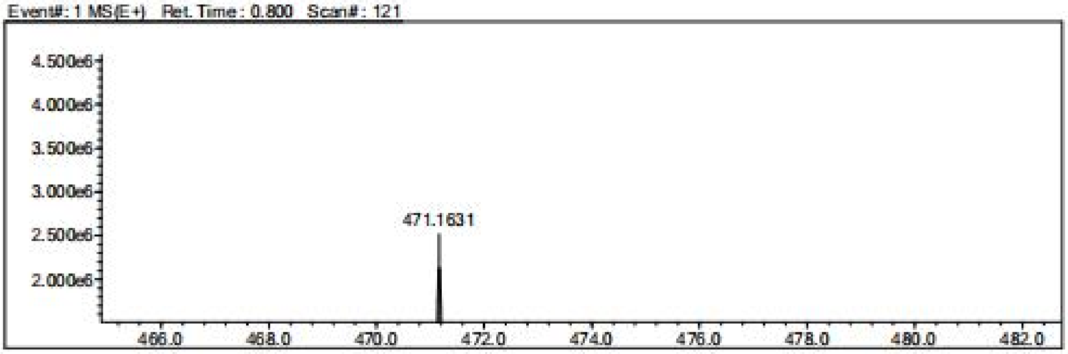

Measured region for 471.1631 m/z

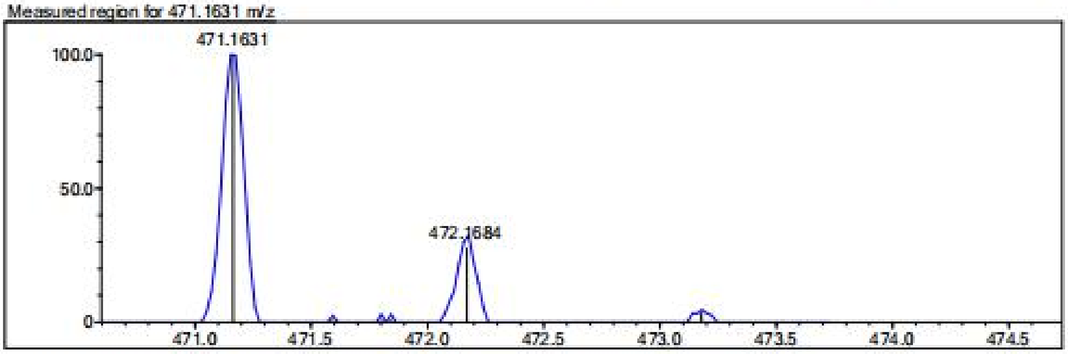

C24 H21 N4 O3 F3 [M+H]+ : Predicted region for 471.1639 m/z

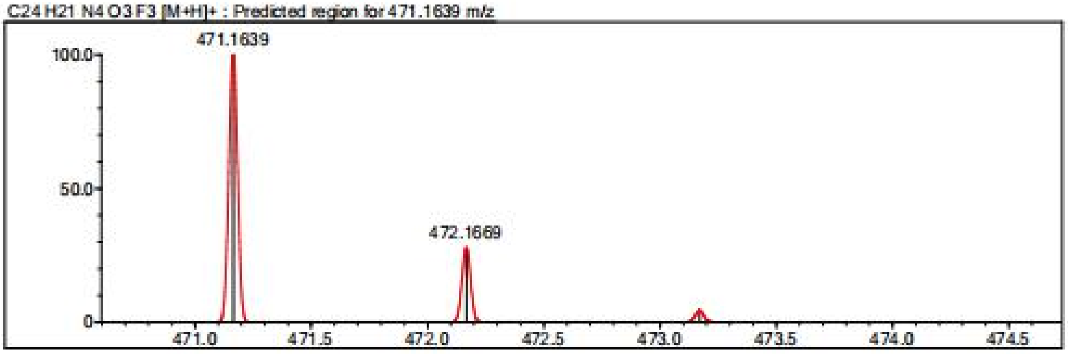

Rank	Score	Formula (M)	Ion	Meas. m/z	Pred. m/z	Df. (mDa)	Df. (ppm)	Iso	DBE
4	90.05	C24 H21 N4 O3 F3	[M+H]+	471.1631	471.1639	-0.8	-1.70	91.66	15.0

HRMS spectrum of **11o**

Elmt	Val.	Min	Max	Elmt	Val.	Min	Max	Elmt	Val.	Min	Max	Use Adduct
H	1	0	25	Al	3	0	0	Zn	2	0	0	K
2H	1	0	0	Si	4	0	0	Se	2	0	0	H
B	3	0	0	P	3	0	0	Br	1	0	0	Na
C	4	0	30	S	2	0	0	Ru	2	0	0	NH4
N	3	0	4	Cl	1	0	0	Sb	3	0	0	
O	2	0	4	Fe	2	0	0	I	3	0	0	
F	1	0	6	Ni	2	0	0	Ir	3	0	0	
Na	1	0	0	Cu	2	0	0	Pt	2	0	0	

Error Margin (ppm): 100
HC Ratio: 0.0 - 100.0
Max Isotopes: all
MSn Iso RI (%): 75.00

DBE Range: -100.0 - 100.0
Apply N Rule: no
Isotope RI (%): 1.00
MSn Logic Mode: OR

Electron Ions: both
Use MSn Info: yes
Isotope Res: 10000
Max Results: 100

Event#: 1 MS(E+) Ret. Time : 0.853 Scan# : 129

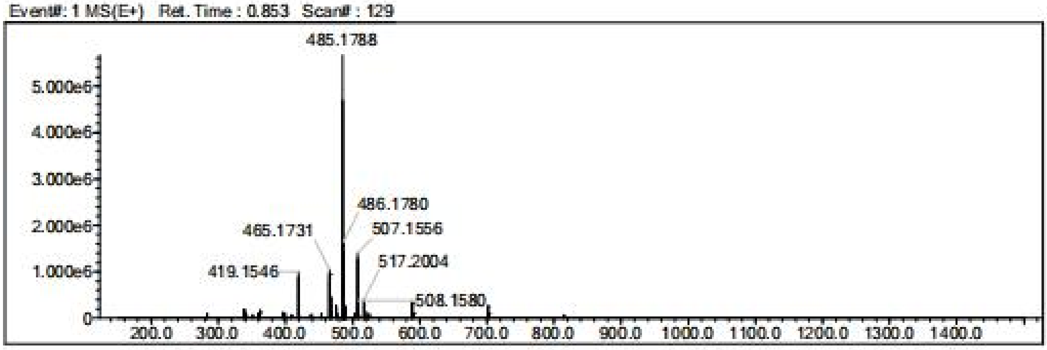

Measured region for 485.1788 m/z

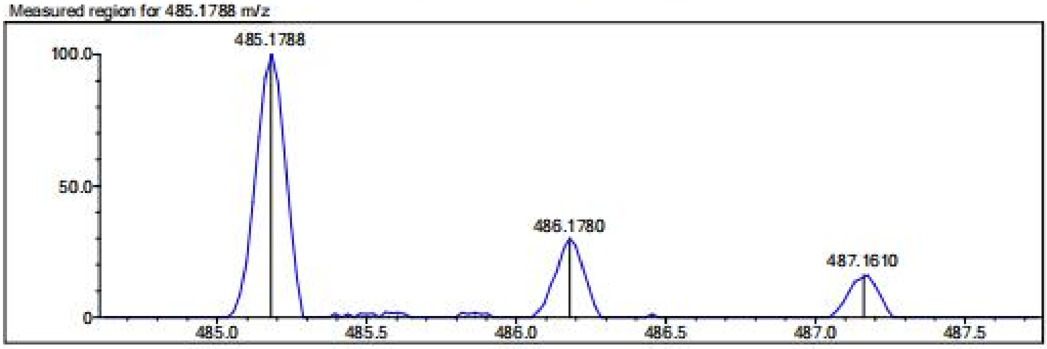

C25 H23 N4 O3 F3 [M+H]+ : Predicted region for 485.1795 m/z

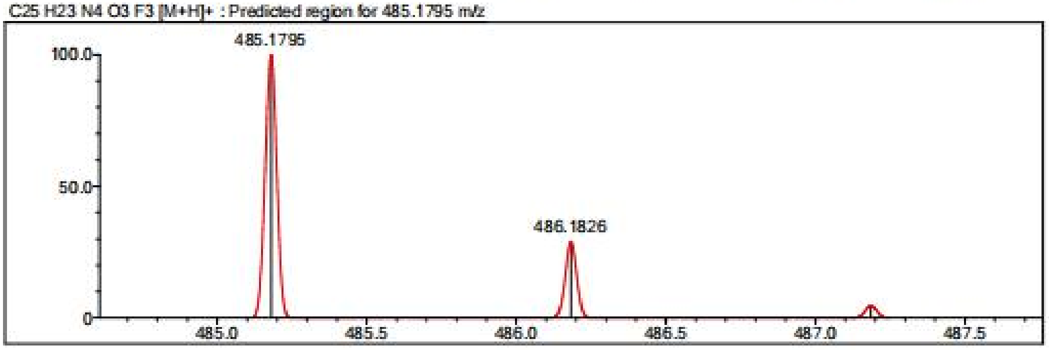

Rank	Score	Formula (M)	Ion	Meas. m/z	Pred. m/z	Df. (mDa)	Df. (ppm)	Iso	DBE
7	36.95	C25 H23 N4 O3 F3	[M+H]+	485.1788	485.1795	-0.7	-1.44	37.37	15.0

HRMS spectrum of **11p**

Elmt	Val.	Min	Max	Elmt	Val.	Min	Max	Elmt	Val.	Min	Max	Use Adduct
H	1	0	25	Al	3	0	0	Zn	2	0	0	K
2H	1	0	0	Si	4	0	0	Se	2	0	0	H
B	3	0	0	P	3	0	0	Br	1	0	0	Na
C	4	0	30	S	2	0	0	Ru	2	0	0	NH4
N	3	0	4	Cl	1	0	0	Sb	3	0	0	
O	2	0	4	Fe	2	0	0	I	3	0	0	
F	1	0	6	Ni	2	0	0	Ir	3	0	0	
Na	1	0	0	Cu	2	0	0	Pt	2	0	0	

Error Margin (ppm): 100
HC Ratio: 0.0 - 100.0
Max Isotopes: all
MSn Iso RI (%): 75.00

DBE Range: -100.0 - 100.0
Apply N Rule: no
Isotope RI (%): 1.00
MSn Logic Mode: OR

Electron Ions: both
Use MSn Info: yes
Isotope Res: 10000
Max Results: 100

Event#: 1 MS(E+) Ret. Time : 0.813 Scan# : 123

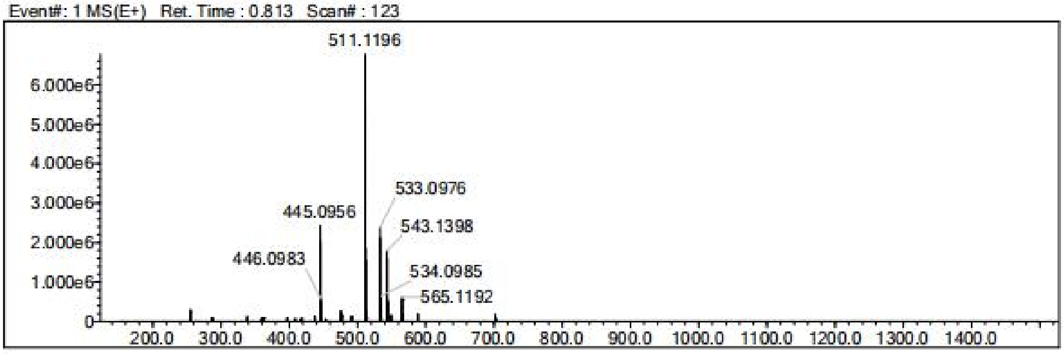

Measured region for 511.1196 m/z

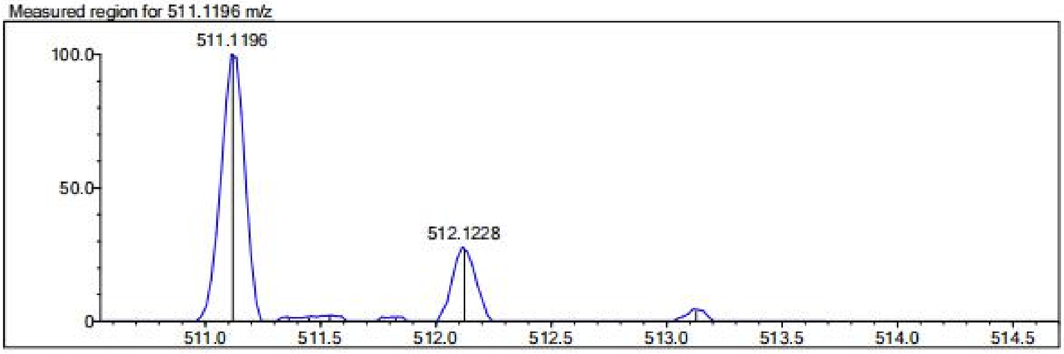

C23 H16 N4 O3 F6 [M+H]+ : Predicted region for 511.1199 m/z

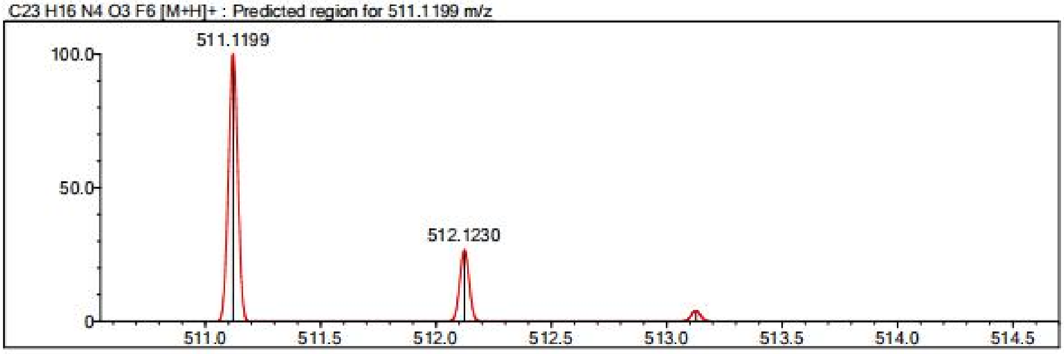

Rank	Score	Formula (M)	Ion	Meas. m/z	Pred. m/z	Df. (mDa)	Df. (ppm)	Iso	DBE
1	85.65	C23 H16 N4 O3 F6	[M+H]+	511.1196	511.1199	-0.3	-0.59	85.65	15.0

HRMS spectrum of **11q**

Elmt	Val.	Min	Max	Elmt	Val.	Min	Max	Elmt	Val.	Min	Max	Use Adduct
H	1	0	25	Al	3	0	0	Zn	2	0	0	K
2H	1	0	0	Si	4	0	0	Se	2	0	0	H
B	3	0	0	P	3	0	0	Br	1	0	0	Na
C	4	0	30	S	2	0	0	Ru	2	0	0	NH4
N	3	0	4	Cl	1	0	0	Sb	3	0	0	
O	2	0	4	Fe	2	0	0	I	3	0	0	
F	1	0	6	Ni	2	0	0	Ir	3	0	0	
Na	1	0	0	Cu	2	0	0	Pt	2	0	0	

Error Margin (ppm): 100 DBE Range: -100.0 - 100.0 Electron Ions: both
HC Ratio: 0.0 - 100.0 Apply N Rule: no Use MSn Info: yes
Max Isotopes: all Isotope RI (%): 1.00 Isotope Res: 10000
MSn Iso RI (%): 75.00 MSn Logic Mode: OR Max Results: 100

Event#: 1 MS(E+) Ret. Time : 0.813 Scan# : 123

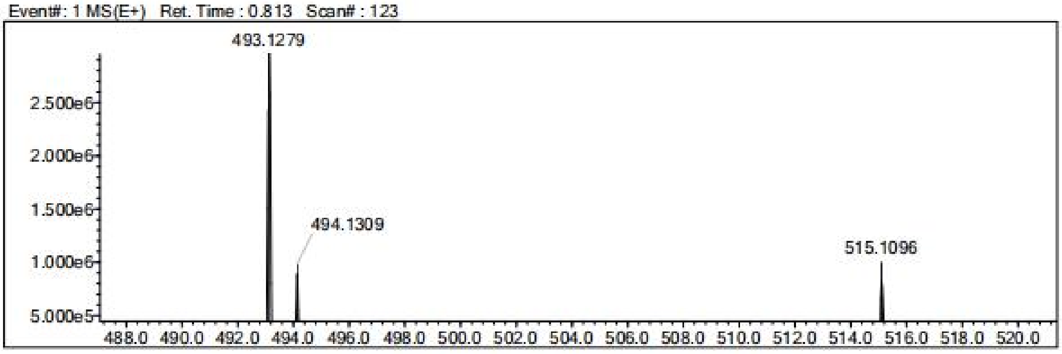

Measured region for 493.1279 m/z

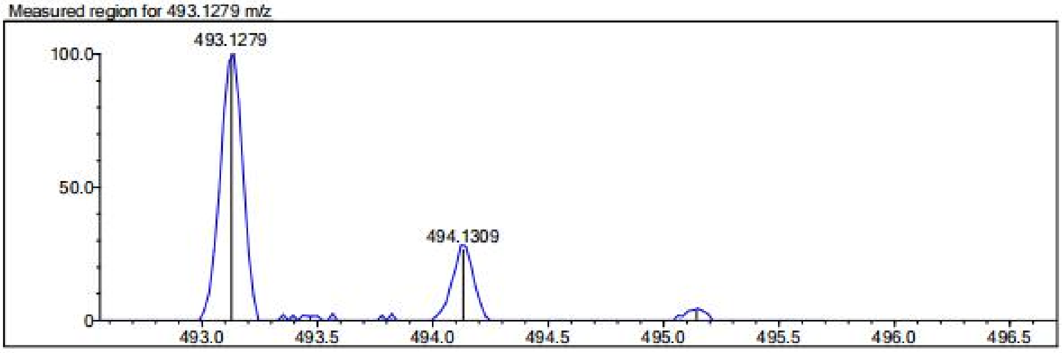

C23 H17 N4 O3 F5 [M+H]+ : Predicted region for 493.1294 m/z

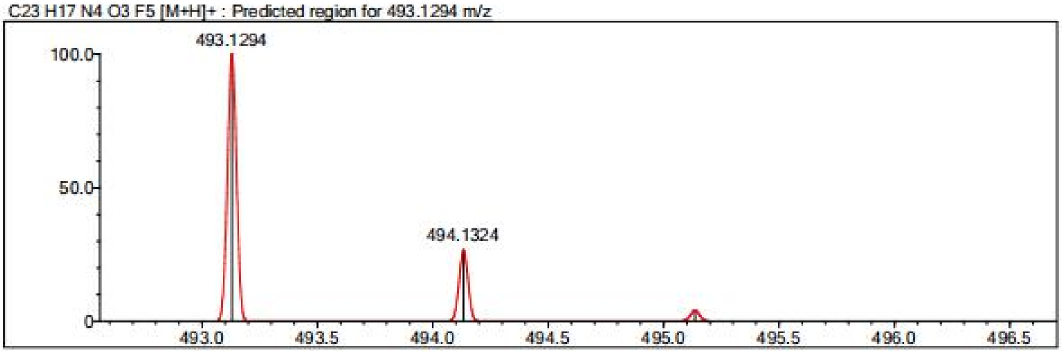

Rank	Score	Formula (M)	Ion	Meas. m/z	Pred. m/z	Df. (mDa)	Df. (ppm)	Iso	DBE
9	76.87	C23 H17 N4 O3 F5	[M+H]+	493.1279	493.1294	-1.5	-3.04	81.00	15.0

HRMS spectrum of **11r**

Elmt	Val.	Min	Max	Elmt	Val.	Min	Max	Elmt	Val.	Min	Max	Use Adduct
H	1	0	23	Al	3	0	0	Zn	2	0	0	K
2H	1	0	0	Si	4	0	0	Se	2	0	0	H
B	3	0	0	P	3	0	0	Br	1	0	0	Na
C	4	0	25	S	2	0	0	Ru	2	0	0	NH4
N	3	0	5	Cl	1	0	0	Sb	3	0	0	
O	2	0	5	Fe	2	0	0	I	3	0	0	
F	1	0	3	Ni	2	0	0	Ir	3	0	0	
Na	1	0	0	Cu	2	0	0	Pt	2	0	0	

Error Margin (ppm): 100
HC Ratio: 0.0 - 100.0
Max Isotopes: all
MSn Iso RI (%): 75.00

DBE Range: -100.0 - 100.0
Apply N Rule: no
Isotope RI (%): 1.00
MSn Logic Mode: OR

Electron Ions: both
Use MSn Info: yes
Isotope Res: 10000
Max Results: 100

Event#: 1 MS(E+) Ret. Time : 0.827 Scan# : 125

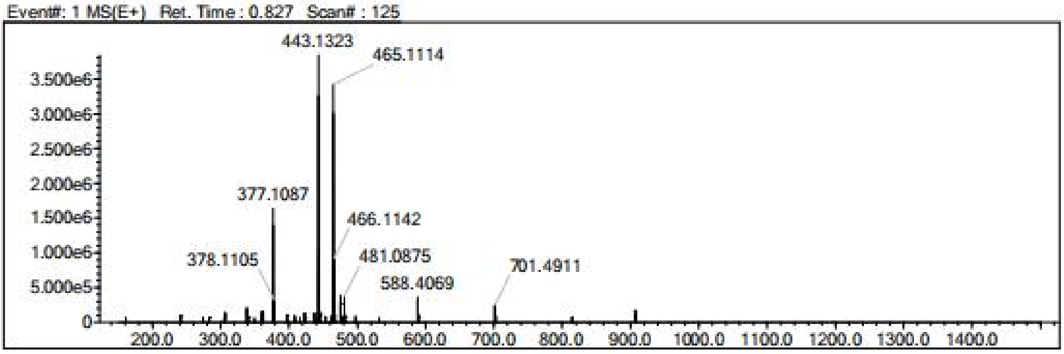

Measured region for 443.1323 m/z

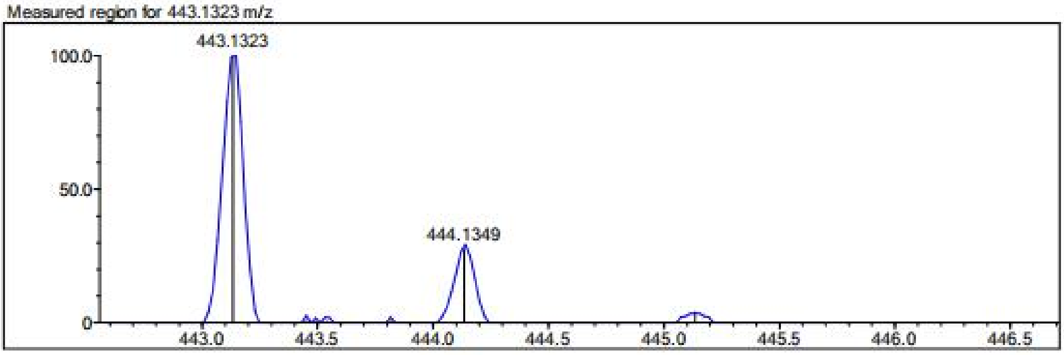

C22 H17 N4 O3 F3 [M+H]+ : Predicted region for 443.1326 m/z

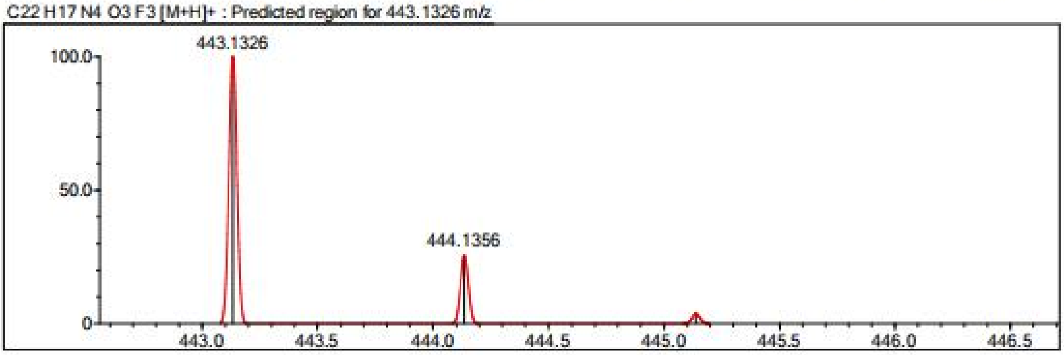

Rank	Score	Formula (M)	Ion	Meas. m/z	Pred. m/z	Df. (mDa)	Df. (ppm)	Iso	DBE
7	72.53	C22 H17 N4 O3 F3	[M+H]+	443.1323	443.1326	-0.3	-0.68	72.53	15.0

HRMS spectrum of **11s**

Elmt	Val.	Min	Max	Elmt	Val.	Min	Max	Elmt	Val.	Min	Max	Use Adduct
H	1	0	21	Al	3	0	0	Zn	2	0	0	K
2H	1	0	0	Si	4	0	0	Se	2	0	0	H
B	3	0	0	P	3	0	0	Br	1	0	0	Na
C	4	0	28	S	2	0	0	Ru	2	0	0	NH4
N	3	0	4	Cl	1	0	0	Sb	3	0	0	
O	2	0	4	Fe	2	0	0	I	3	0	0	
F	1	0	3	Ni	2	0	0	Ir	3	0	0	
Na	1	0	0	Cu	2	0	0	Pt	2	0	0	

Error Margin (ppm):	100	DBE Range:	-100.0 - 100.0	Electron Ions:	both
HC Ratio:	0.0 - 100.0	Apply N Rule:	no	Use MSn Info:	yes
Max Isotopes:	all	Isotope RI (%):	1.00	Isotope Res:	10000
MSn Iso RI (%):	75.00	MSn Logic Mode:	OR	Max Results:	100

Event#: 1 MS(E+) Ret. Time : 0.853 Scan# : 129

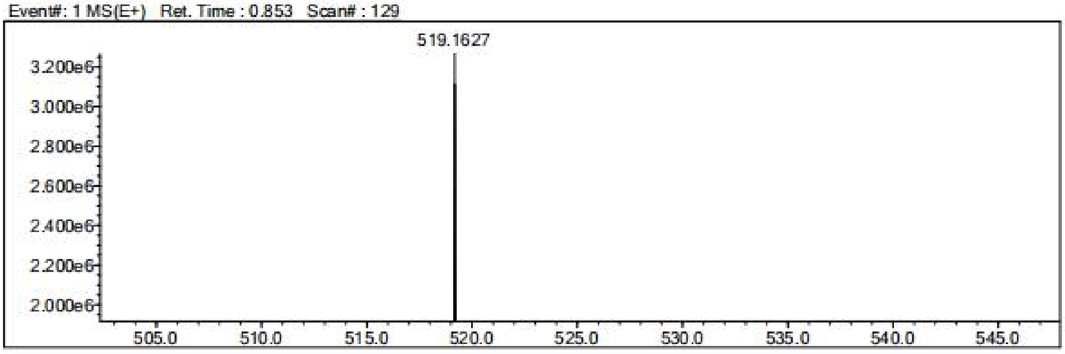

Measured region for 519.1627 m/z

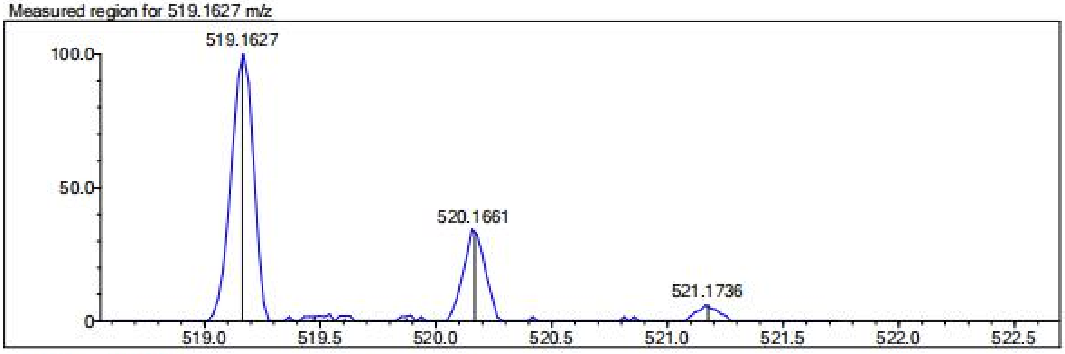

C28 H21 N4 O3 F3 [M+H]+ : Predicted region for 519.1639 m/z

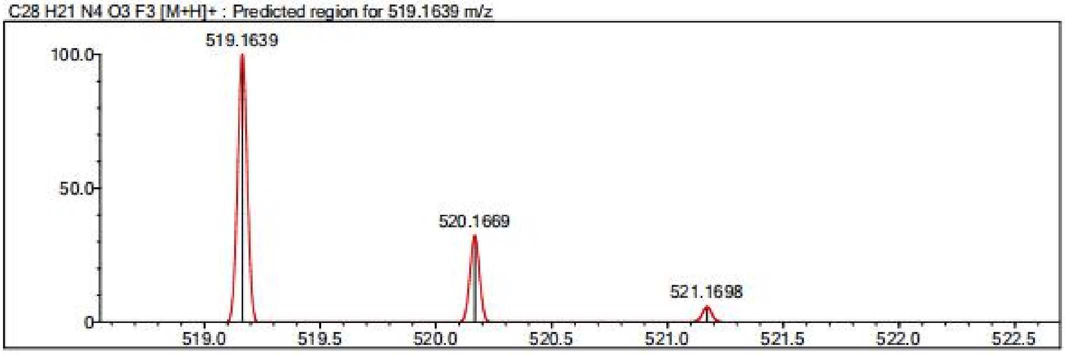

Rank	Score	Formula (M)	Ion	Meas. m/z	Pred. m/z	Df. (mDa)	Df. (ppm)	Iso	DBE
1	87.88	C28 H21 N4 O3 F3	[M+H]+	519.1627	519.1639	-1.2	-2.31	90.85	19.0

HRMS spectrum of **11t**

Elmt	Val.	Min	Max	Elmt	Val.	Min	Max	Elmt	Val.	Min	Max	Use Adduct
H	1	0	25	Al	3	0	0	Zn	2	0	0	K
2H	1	0	0	Si	4	0	0	Se	2	0	0	H
B	3	0	0	P	3	0	0	Br	1	0	0	Na
C	4	0	30	S	2	0	0	Ru	2	0	0	NH4
N	3	0	4	Cl	1	0	1	Sb	3	0	0	
O	2	0	4	Fe	2	0	0	I	3	0	0	
F	1	0	6	Ni	2	0	0	Ir	3	0	0	
Na	1	0	0	Cu	2	0	0	Pt	2	0	0	

Error Margin (ppm): 100
HC Ratio: 0.0 - 100.0
Max Isotopes: all
MSn Iso RI (%): 75.00

DBE Range: -100.0 - 100.0
Apply N Rule: no
Isotope RI (%): 1.00
MSn Logic Mode: OR

Electron Ions: both
Use MSn Info: yes
Isotope Res: 10000
Max Results: 100

Event#: 1 MS(E+) Ret. Time : 0.933 Scan# : 141

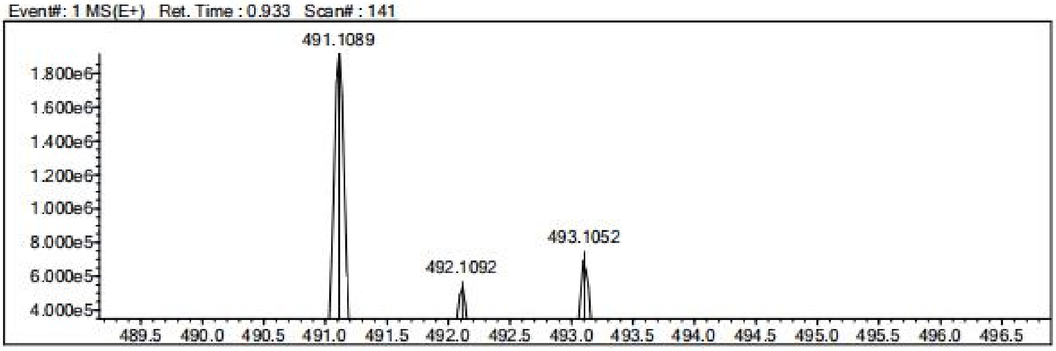

Measured region for 491.1089 m/z

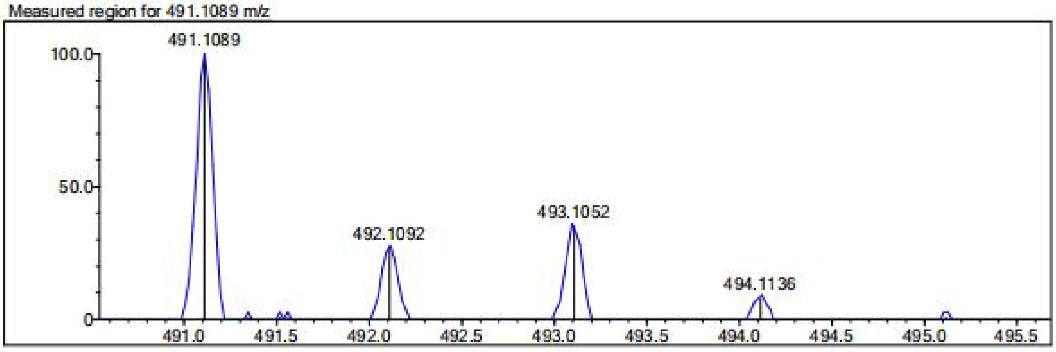

C23 H18 N4 O3 F3 Cl [M+H]+ : Predicted region for 491.1092 m/z

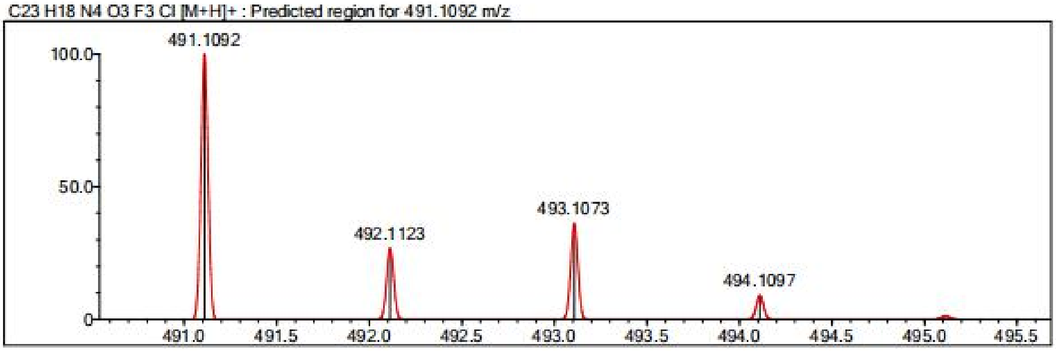

Rank	Score	Formula (M)	Ion	Meas. m/z	Pred. m/z	Df. (mDa)	Df. (ppm)	Iso	DBE
1	94.98	C23 H18 N4 O3 F3 Cl	[M+H]+	491.1089	491.1092	-0.3	-0.61	94.98	15.0

HRMS spectrum of **16**

Elmt	Val.	Min	Max	Elmt	Val.	Min	Max	Elmt	Val.	Min	Max	Use Adduct
H	1	0	23	Al	3	0	0	Zn	2	0	0	K
2H	1	0	0	Si	4	0	0	Se	2	0	0	H
B	3	0	0	P	3	0	0	Br	1	0	0	Na
C	4	0	25	S	2	0	0	Ru	2	0	0	NH4
N	3	0	5	Cl	1	0	0	Sb	3	0	0	
O	2	0	5	Fe	2	0	0	I	3	0	0	
F	1	0	3	Ni	2	0	0	Ir	3	0	0	
Na	1	0	0	Cu	2	0	0	Pt	2	0	0	

Error Margin (ppm):	100	DBE Range:	-100.0 - 100.0	Electron Ions:	both
HC Ratio:	0.0 - 100.0	Apply N Rule:	no	Use MSn Info:	yes
Max Isotopes:	all	Isotope RI (%):	1.00	Isotope Res:	10000
MSn Iso RI (%):	75.00	MSn Logic Mode:	OR	Max Results:	100

Event#: 1 MS(E+) Ret. Time : 0.880 Scan# : 133

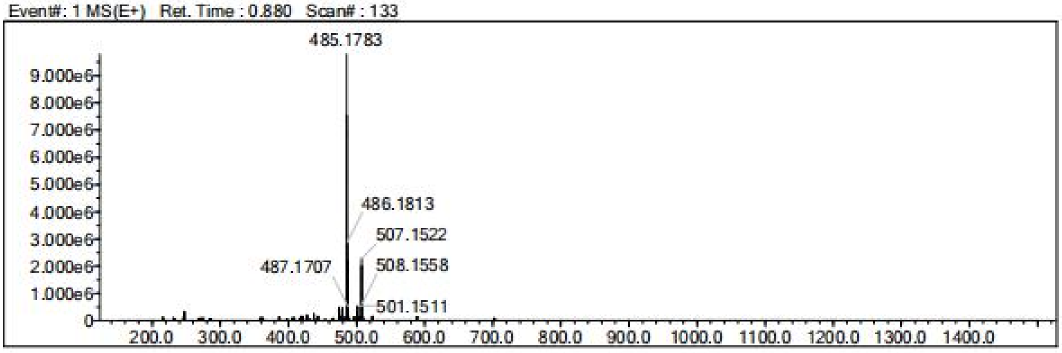

Measured region for 485.1783 m/z

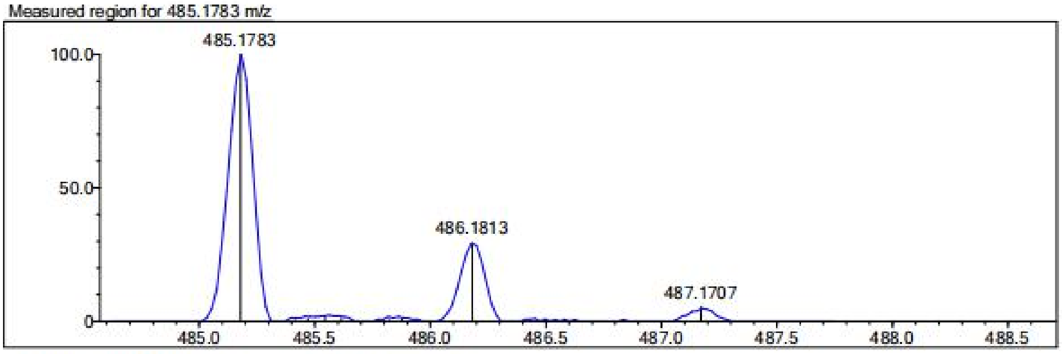

C25 H23 N4 O3 F3 [M+H]+ : Predicted region for 485.1795 m/z

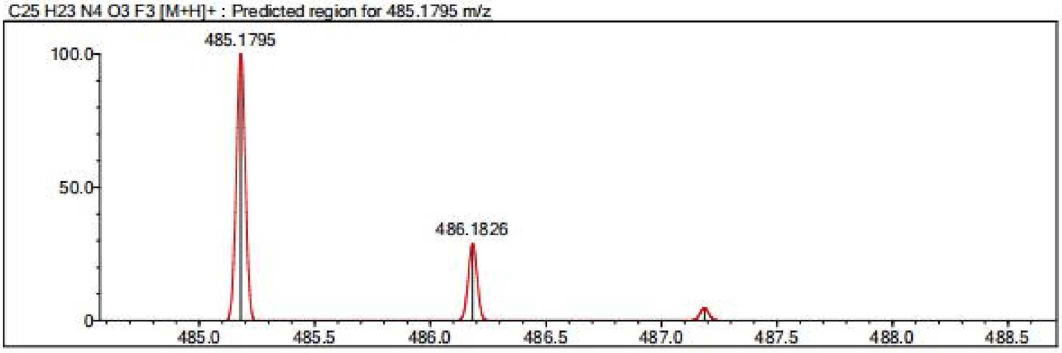

Rank	Score	Formula (M)	Ion	Meas. m/z	Pred. m/z	Df. (mDa)	Df. (ppm)	Iso	DBE
2	73.54	C25 H23 N4 O3 F3	[M+H]+	485.1783	485.1795	-1.2	-2.47	76.34	15.0

3. Chiral Resolution of Compound 11h

Considering the effect of chiral structure on the biological activity of compounds, the chiral isomers **(-)-11h** and **(+)-11h** were prepared by the separation of **11h** with chiral HPLC. The chiral resolution of compound **11h** was performed using Gilson instruments, and the chromatographic column was a CHIRALPAK®IF column (250 mm × 4.6 mm, 5 μm). The compound was filtered with a microporous membrane with an injection volume of 5 μL, and the mobile phase was Hex/EtOH = 80:20. The eluent was ultrasonically degassed for 30 min before use. Flow rate: 1 mL/min; Cycle time: 40 min. the chiral isomers **(-)-11h** ($[\alpha]_D^{20}$ = - 46.7 (c 1.0, THF), t_R = 24.847 min, ee value, > 99%) and **(+)-11h** ($[\alpha]_D^{20}$ = + 46.5 (c 1.0, THF), t_R = 33.976 min, ee value, > 99%) were prepared by the separation of **(±)-11h** with chiral HPLC.

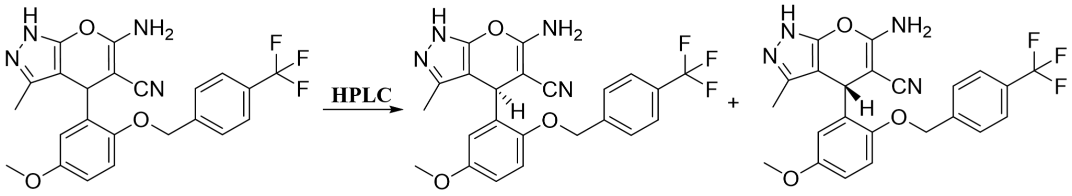

11h **(-)-11h** **(+)-11h**

Scheme 1. Reagents and conditions：AD-H column. Mobile phase: hex: EtOH=80:20, flow rate: 1 mL/min.

stereoisomers separation HPLC chromatogram.

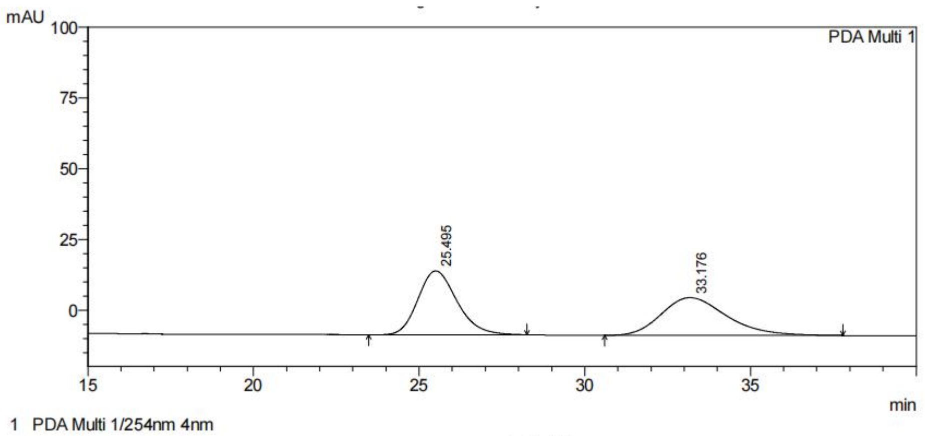

PeakTable

PDA Ch1 254nm 4nm

Peak#	Ret. Time	Area	Height	Area %
1	25.495	1852714	22589	50.282
2	33.176	1831953	13363	49.718
Total		3684666	35952	100.000

HPLC chromatogram of (±)-11h

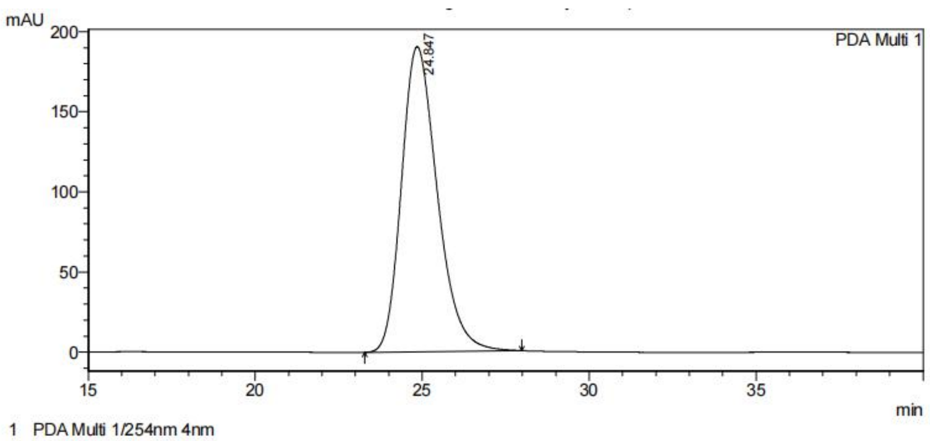

PeakTable

PDA Ch1 254nm 4nm

Peak#	Ret. Time	Area	Height	Area%
1	24.847	14198971	190395	100.000
Total		14198971	190395	100.000

HPLC chromatogram of (-)-11h

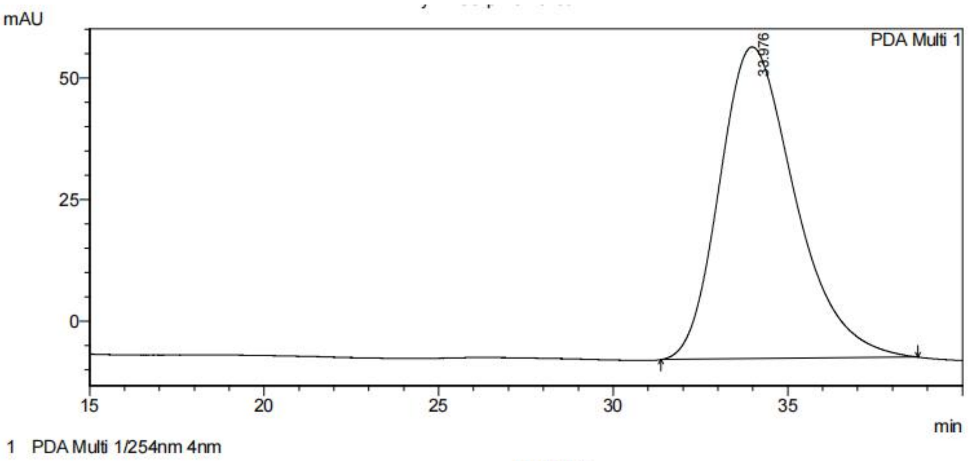

PeakTable

PDA Ch1 254nm 4nm

Peak#	Ret. Time	Area	Height	Area%
1	33.976	9647987	64117	100.000
Total		9647987	64117	100.000

HPLC chromatogram of (+)-11h
